# Evolution of Avian Influenza Virus (H3) with Spillover into Humans, China

**DOI:** 10.3201/eid2906.221786

**Published:** 2023-06

**Authors:** Jiaying Yang, Ye Zhang, Lei Yang, Xiyan Li, Hong Bo, Jia Liu, Min Tan, Wenfei Zhu, Yuelong Shu, Dayan Wang

**Affiliations:** National Institute for Viral Disease Control and Prevention, Chinese Center for Disease Control and Prevention, Key Laboratory for Medical Virology, Beijing, China (J. Yang, Y. Zhang, L. Yang, X. Li, H. Bo, J. Liu, M. Tan, W. Zhu, D. Wang);; School of Public Health (Shenzhen), Shenzhen campus of Sun Yat-sen University, Shenzhen, China (J. Yang, Y. Shu);; Institute of Pathogen Biology of Chinese Academy of Medical Science/Peking Union Medical College, Beijing (Y. Shu)

**Keywords:** Avian influenza virus, H3, reassortment, viruses, genotype, surveillance, humans, China, influenza

## Abstract

The continuous evolution of avian influenza viruses (AIVs) of subtype H3 in China and the emergence of human infection with AIV subtype H3N8 highlight their threat to public health. Through surveillance in poultry-associated environments during 2009–2022, we isolated and sequenced 188 H3 AIVs across China. Performing large-scale sequence analysis with publicly available data, we identified 4 sublineages of H3 AIVs established in domestic ducks in China via multiple introductions from wild birds from Eurasia. Using full-genome analysis, we identified 126 distinct genotypes, of which the H3N2 G23 genotype predominated recently. H3N8 G25 viruses, which spilled over from birds to humans, might have been generated by reassortment between H3N2 G23, wild bird H3N8, and poultry H9N2 before February 2021. Mammal-adapted and drug-resistance substitutions occasionally occurred in H3 AIVs. Ongoing surveillance for H3 AIVs and risk assessment are imperative for potential pandemic preparedness.

Avian influenza viruses (AIVs) of subtype H3 are highly prevalent among waterfowl globally, causing mild or no apparent signs of illness in birds (*1*–*5*). H3 AIV has shown the potential for cross-species transmission and was the origin of other animal influenza viruses, which caused epidemics in horses, dogs, seals, and pigs (*6*–*9*). In 1968, H3 AIV contributed its hemagglutinin (HA) gene to the human influenza (H3N2) pandemic viruses, and it is still unknown whether an intermediate host was involved (*10*). 

In April 2022, the first human infection with AIV (H3N8) was reported; the case was in a 4-year-old boy whose family reared chickens and silky fowls in Henan Province, China (*11*). After infection, the patient exhibited recurrent fever and severe pneumonia. In May 2022, a second case was identified in 5-year-old boy with mild influenza symptoms, who had visited the live poultry market (LPM) in Hunan Province, China (*12*). Those cases raised concern over whether H3N8 AIVs will cause a major public health threat (*13*).

In China, H3 AIVs have been dynamically circulating in poultry and wild birds across multiple regions (*14*). H3 combinations with multiple neuraminidase (NA) subtypes (N1–N8) were reported, among which H3N2 and H3N8 predominated (*14*–*16*). Phylogenetically, those viruses belonged to the Eurasian lineage, which is widespread in wild birds across Eurasia (*3*,*14*,*17*,*18*). Reassortment events often occurred at LPMs (*16*,*19*–*22*). During 2009–2022, we conducted country-level AIV surveillance in poultry-associated environments and performed a large-scale genetic analysis to provide a comprehensive picture of the evolution of H3 AIVs in China.

## Methods

During January 2009–June 2022, we collected environmental samples monthly from avian-linked environments across 31 provinces in the China mainland according to AIV surveillance guideline of Chinese Center for Disease Control and Prevention. We isolated and sequenced 188 H3 viruses (32 have been previously published [*15*]). The sequences were deposited in the GISAID EpiFlu database (https://www.gisaid.org; accession nos. EPI2210281–1516) (Appendix Table 1).

We performed sequence alignments with available sequences from the GISAID EpiFlu database as of June 25, 2022, by using MAFFT version 7.222 (*23*). We reconstructed maximum-likelihood phylogenies of all segments by using FastTree version 2.1.11 (*24*). The resulting trees were classified into divergent lineages or sublineages. Genotypes were assigned by the combination of lineages for each segment of full-genome viruses.

To estimate the time to the most recent common ancestor (tMRCA) of H3N8 viruses of humans, we used Bayesian Markov chain Monte Carlo analyses for each gene in BEAST version 1.10.4 (*25*). We then generated maximum clade credibility trees (Appendix).

## Results

### Isolation and Sequencing of H3 AIVs 

**Figure 1:**
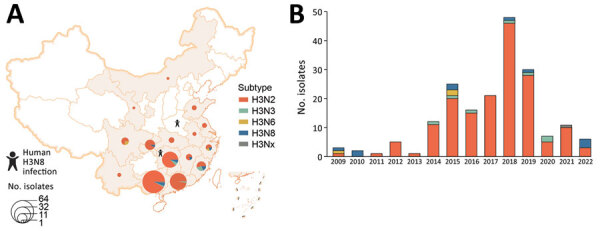
Spatial and temporal distribution of avian influenza virus subtype H3 isolated from poultry-associated environments, China, 2009–2022. A) Spatial distribution of environmental H3 subtype viruses. One H3 isolate without neuraminidase (NA) subtype was designated as H3Nx. Provinces where human infections with H3N8 were reported are noted. B) Number of environmental H3 subtype isolates per year. This figure includes all H3 isolates sequenced by the Chinese National Influenza Center. Additional metadata are available in Appendix Table 1.

During January 2009–June 2022, we isolated 188 H3 AIVs from the poultry-associated environmental samples: 167 H3N2, 7 H3N3, 3 H3N6, 10 H3N8, and 1 H3 with NA unknown (Appendix Table 1). The H3N2 AIVs were widely distributed across 15 provinces, mainly in southern China (Figure 1, panel A). We isolated H3 AIVs with other NA subtypes (N3, N6, and N8) in 2–8 provinces. More than three quarters of the H3 viruses (149/188, 79.3%) were isolated from the samples collected from LPMs (Appendix Table 2). Before 2014, we isolated and sequenced <6 strains of H3 AIVs per year (Figure 1, panel B). Since 2014, we obtained more isolates, most (48) in 2018. All H3 isolates were sequenced, and we recovered the full genomes from 185 of the isolates (Appendix Table 1).

### Evolution of H3 Genes in China

**Figure 2:**
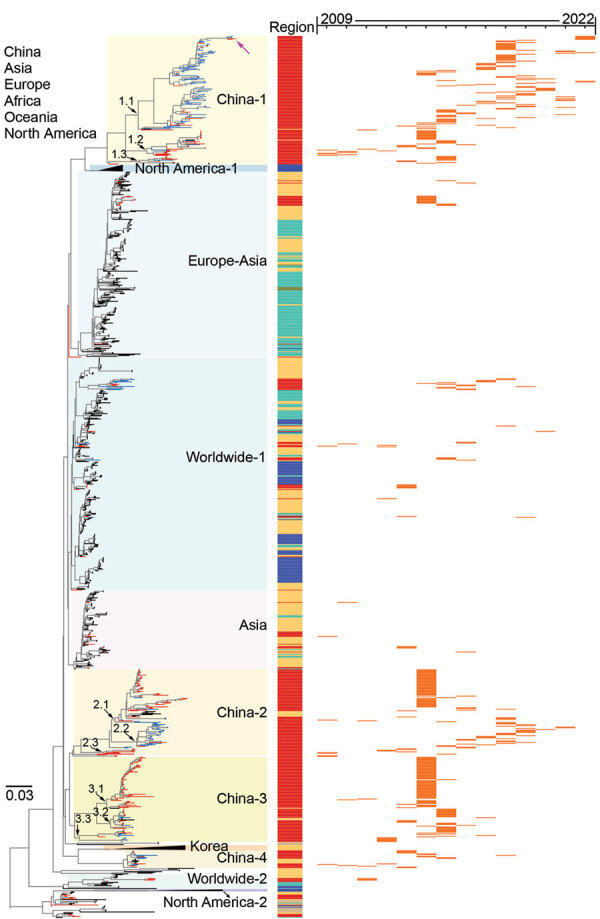
Maximum-likelihood phylogenetic tree of hemagglutinin genes of avian influenza viruses subtype H3 from China (n = 1,291) and reference sequences from GISAID (https://www.gisaid.org). Blue tree sections indicate sequences reported in this study; red tree sections indicate other H3 sequences from China; violet arrow at top of tree indicates human H3N8 virus. For clarity, some clades are collapsed. Sublineages are shown with different background colors on the phylogenetic tree. Subgroups in sublineages China-1, China-2, and China-3 are marked with black arrows at the nodes. The sampling locations are annotated with colored bars adjacent to the tree. For the H3 viruses sampled in China during 2009–2022, the sampling year of each of these viruses is shown on the right panel with orange horizontal bars. The phylogenetic tree of the H3 genes with more detailed information is shown in Appendix Figure 1. Scale bar indicates nucleotide substitutions per site.

To elucidate the evolution of H3 AIVs in China, we performed a phylogenetic analysis of HA genes of the H3 AIVs sequenced in this study, along with sequences available from the GISAID EpiFlu Database (Figure 2). The HA genes of all viruses in this study were grouped into the Eurasian lineage, sharing a nucleotide homology of 79.2%–100.0%. In brief, the major branch of Eurasian avian H3 lineage containing viruses in recent decades could be further classified into 10 sublineages (named by the geographic distributions: China-1, China-2, China-3, China-4, Asia, Europe-Asia, worldwide-1, worldwide-2, North America-1, and Korea); other minor branches at the bottom of the phylogenetic tree included the North America-2 sublineage and early strains sampled during 1972–1992 (Figure 2; Appendix Figure 1). H3 AIVs collected from wild birds, poultry, or poultry-associated environments in China in recent decades were distributed in 8 sublineages, except sublineages North America-1, North America-2, and Korea, which were only identified in North America and South Korea.

Sublineages China-1, China-2, China-3, and China-4 consisted of AIVs almost all collected from poultry or poultry-associated environments in China in addition to a few viruses from Vietnam (18) and Cambodia (1) (Appendix Figure 1). Domestic ducks acted as the main host for China-1 (48/166), China-2 (63/111), China-3 (80/110), and China-4 (15/23) (Appendix Table 3). Each sublineage comprised various NA subtypes (Appendix Figure 1). The most common subtype was H3N2 (270), followed by H3N8 (41), H3N6 (19), H3N3 (12), and H3N9 (1), except for 67 H3 AIVs with NA unknown. A high proportion (397/410, 96.8%) of these viruses have been sampled since 2009, whereas recent isolates were primarily consolidated in the China-1 and China-2 sublineages (Figure 2).

The China-1 sublineage had evolved into 3 distinct subgroups, with prevalence spanning different times. Most of our isolates (101/185, 54.6%) fell into the China-1.1 subgroup, which circulated during 2008–2022. Of note, 3 H3N8 strains sampled in Fujian (2) and Guangxi (1) Provinces in 2022 had a close relationship with 2 human H3N8 strains and together formed a miniature phylogenetic group (Appendix Figure 1). The China-1.2 subgroup was detected during 2009–2016 and the China-1.3 subgroup during 2013–2015 (Figure 2; Appendix Figure 1).

The China-2 and China-3 sublineages have evolved into 3 subgroups, and the China-2.2 subgroup mainly comprised environmental H3 viruses (29/31, 93.5%) sequenced in this study during 2015–2021 (Figure 2; Appendix Figure 1). H3 viruses of sublineages Asia, Europe-Asia, worldwide-1, and worldwide-2 were occasionally detected in poultry and wild birds in China, but no stable cluster was established (Appendix Figure 1).

### Reassortment with NA Genes

We detected multiple NA subtypes in each H3 sublineage. We performed phylogenetic analyses for 4 major NA subtypes: N2, N3, N6, and N8. Almost all NA genes of H3 AIVs in our study were clustered within the Eurasian lineage, and 8 H3N8 AIVs had NA genes derived from the North American lineage (Appendix Figure 2, panels A–D).

The N2 genes of AIVs in the Eurasian lineage could be further classified into sublineages, and most H3N2 viruses in this study were clustered in the Eurasian-2 sublineage (Appendix). We also found H3N3 strains closely related to the human-origin influenza (H10N3) virus and H3N6 closely related to highly pathogenic AIV (HPAIV) subtype H5N6 (Appendix).

**Figure 3:**
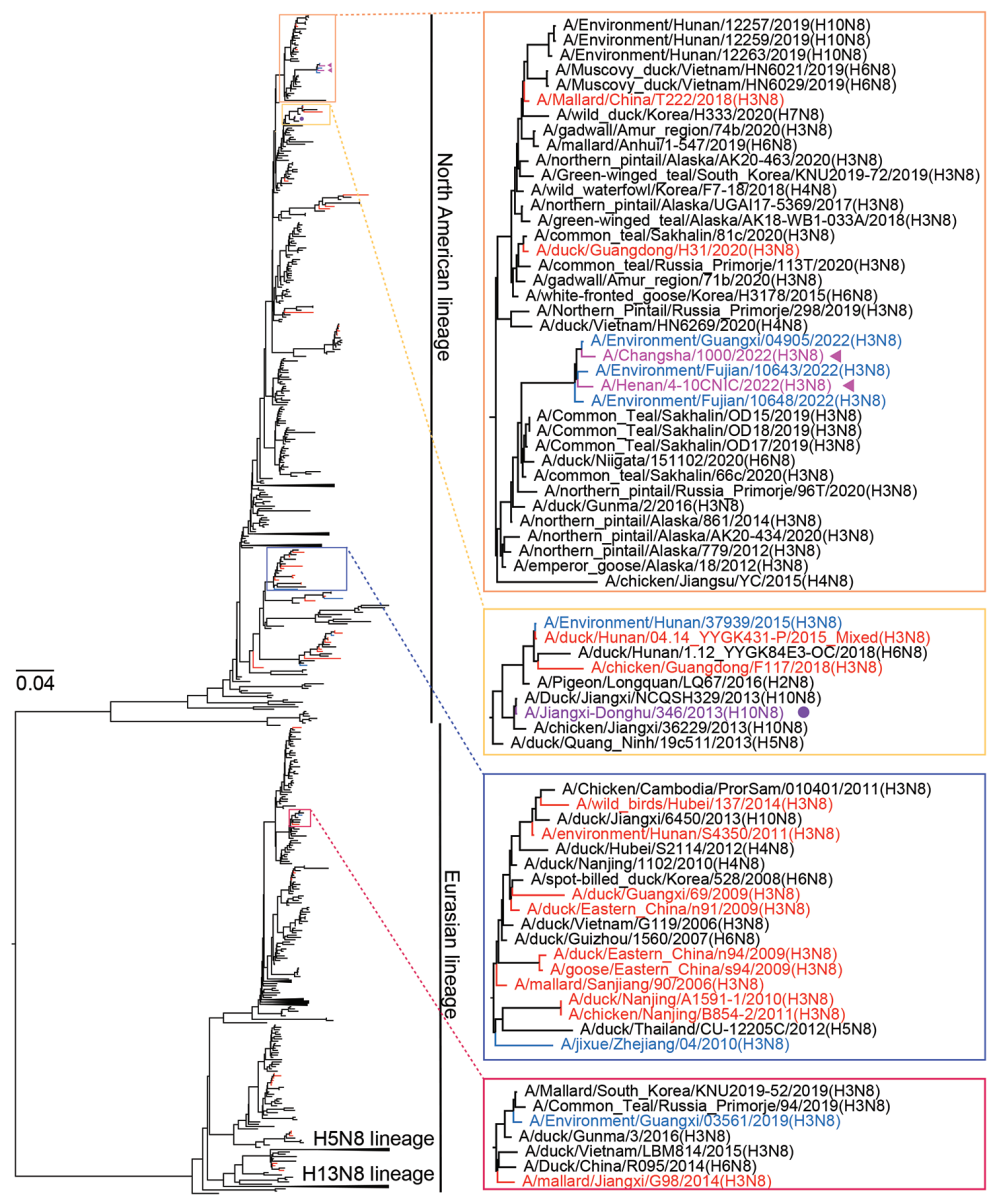
Maximum-likelihood phylogenetic tree of avian influenza virus subtype N8 genes from China (n = 1,106) and reference sequences from GISAID (https://www.gisaid.org). Blue tree sections indicate sequences of H3 subtype viruses reported in this study; red tree sections indicate other H3 subtype viruses from China. For of clarity, some clades are collapsed. Representative clusters are indicated in shaded boxes and magnified on the right. Violet arrows indicate human H3N8 viruses; purple solid circle indicates human H10N8 virus. The phylogenetic tree of N8 genes with more complete information is shown in Appendix Figure 2, panel D). Scale bar indicates nucleotide substitutions per site.

Most NA genes of H3N8 viruses (43/59) from China belonged to the North American lineage, closely related to AIVs from different regions (e.g., Russia, Vietnam, South Korea, and North America). Of note, the NA genes of human H3N8 and H10N8 viruses belonged to distinct groups (Figure 3), and 3 environmental strains sequenced in this study were highly homologous to the human H3N8 viruses. Few H3N8 strains from China fell into the Eurasian lineage (Figure 3).

### Reassortment with Internal Genes

In the phylogenetic tree of each internal gene, a large proportion of H3 AIVs in China belonged to the Eurasian wild bird reservoir (Appendix Figure 3). Some H3 AIVs had internal genes derived from ZJ-5 sublineage (of the wild bird viruses), poultry H5N1/H5N6 sublineage, poultry H9N2 ZJ-HJ/07 sublineage, or waterfowl H6 sublineage (Appendix). Each internal gene has only 1 or 2 virus sequences that belong to the H9N2 ZJ-HJ/07 sublineage. In 2022, a total of 3 environmental and 2 human H3N8 viruses contained all internal genes belonging to the H9N2 ZJ-HJ/07 sublineage.

### Emergence of Multiple Genotypes

Assessment of the diversity of genome constellations indicated that prolific reassortments of the H3 AIVs had occurred in China in past decades. On the basis of the sublineage classification of all 8 gene segments, we identified 126 genotypes among 284 full-genome H3 viruses sampled in China during 2009–2022 (Appendix Figure 4). We found evidence of dynamic emergence for 73 genotypes (G1–G73) from 212 H3N2 genomes, 11 genotypes (G1–G11) from 14 H3N3, 17 (G1–G17) from 25 H3N6, and 25 (G1–G25) from 33 H3N8 (Appendix). H3N2 G23 had been detected in multiple years and provinces during 2014–2022 (Appendix Figure 4, panel A, Figure 5, panel A). H3N8 G25, which had been detected in both environmental and human viruses in 2022, acquired HA genes from the China-1 H3 sublineage, NA genes from the North American N8 lineage, and all 6 internal gene from poultry H9N2 ZJ-HJ/07 sublineage viruses (Appendix Table 4).

### Emergence of H3N8 G25 Viruses

We further traced the origin of the H3N8 G25 viruses. When we compared the genetic diversity of G25 genotype viruses, the results showed that these viruses shared a higher similarity in HA (98.4%–99.1%) and NA genes (98.8%–99.3%) and a lower similarity in other internal genes (polymerase basic [PB] 2, 93.9%–100.0%; PB1, 91.6%–99.9%; polymerase acidic [PA], 93.4%–99.6%; nucleocapsid, 94.5%–99.9%; matrix (M), 95.3%–100.0%; and nonstructural, 97.0%–98.7%). This finding indicated that after the emergence of prior H3N8 G25 virus, dynamic reassortment might occur between H3N8 and poultry H9N2 viruses.

**Figure 4:**
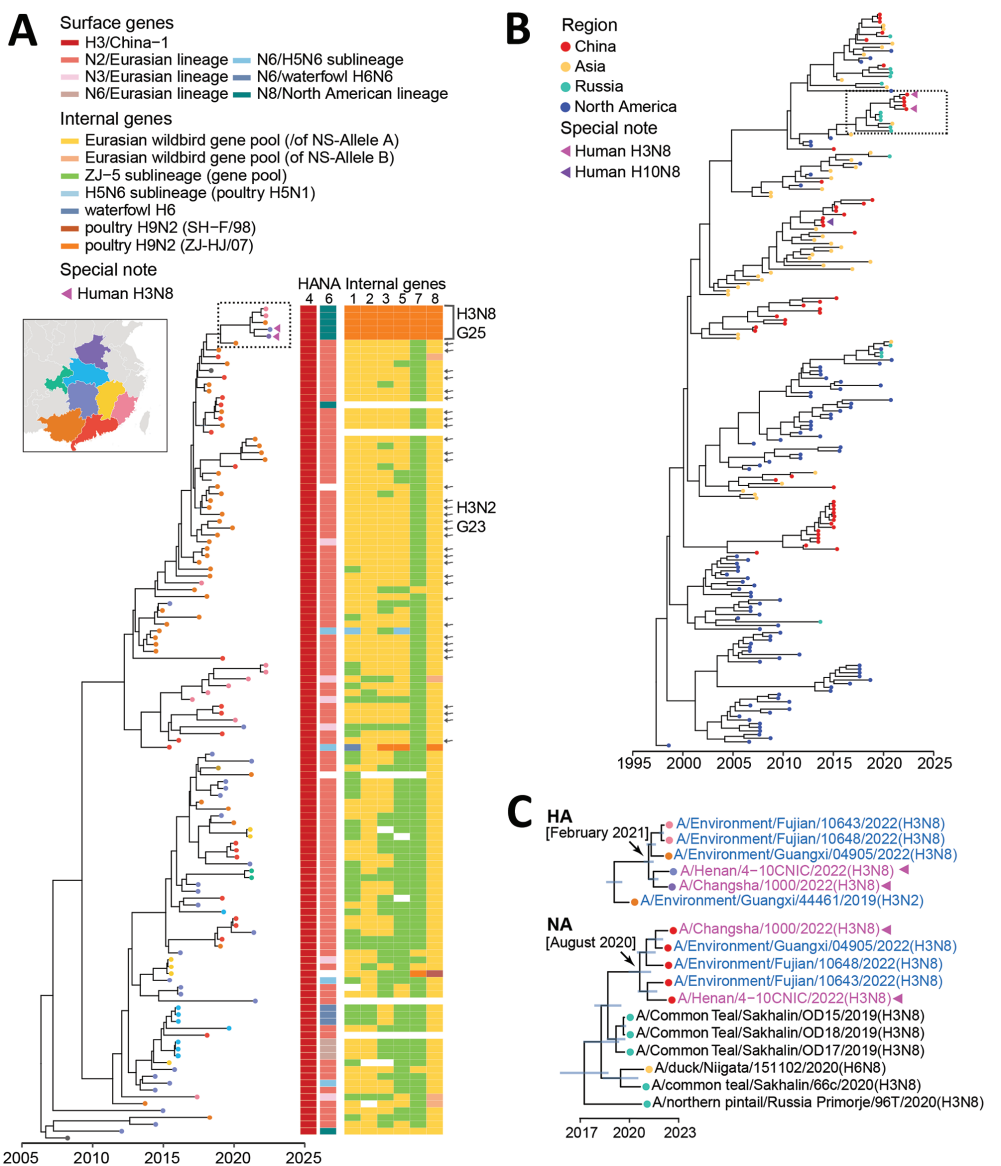
Bayesian time-resolved phylogenetic tree of hemagglutinin (HA) genes from avian influenza subtype H3 viruses and neuraminidase (NA) genes from subtype N8 viruses from China and reference sequences from GISAID (https://www.gisaid.org). A) Maximum clade credibility tree of HA genes of the China-1.1 H3 subgroup (n = 122). Tip points are colored by provinces (corresponding to the fill color in the map). Violet triangles indicate human H3N8 viruses. The lineage origins of each gene segment of H3 AIVs are represented by different colored tiles adjacent to the tree; the tile is blank if the sequence is unavailable. H3N2 G23 viruses are indicated with arrows. H3N8 G25 viruses are indicated within the bracket. The fully resolved tree with detailed information is depicted in Appendix Figure 6. B) Maximum clade credibility tree of N8 genes (n = 202). Tip points are colored by region. Violet triangles indicate human H3N8 viruses; purple triangle indicates human H10N8 virus. Virus names of the representative cluster (in the dashed box) are shown in panel C. The fully resolved tree with detailed information is depicted in Appendix Figure 7. C) Clades in the dashed box in panels A and B. Trees are drawn to the same scale. Blue indicates H3 avian influenza viruses sequenced in this study; violet indicates human H3N8 viruses. For HA (top) and NA (bottom) genes, branch tips are colored as in panels A and B. Blue node bars correspond to the 95% credible intervals of node heights. Arrows indicate the most recent common ancestors of HA and NA genes of H3N8 G25 viruses.

To elucidate the timing of H3N8 G25 virus emergence, we performed coalescent analyses and calculated the estimated tMRCA of all 8 segments (Appendix Figures 6–13). The median tMRCA among the HA genes was estimated to be February 2021 (95% highest posterior density [HPD] October 2020–May 2021). The HA genes closely related to those of H3N8 G25 viruses were from H3N2 G23 AIVs isolated from Guangxi and Guangdong Provinces, particularly A/environment/Guangxi/44461/2019 (H3N2), sampled in December 2019 (Figure 4, panels A, C). The median tMRCA among the NA genes of the H3N8 G25 viruses was estimated to be August 2020 (95% HPD November 2019–March 2021). H6N8 AIV isolated in Japan and H3N8 AIV isolated in the Russian Far East during 2019–2020 were closely related to H3N8 G25 viruses, specifically A/common teal/Sakhalin/OD17/2019 (H3N8) virus (Figure 4, panels B, C).

**Figure 5:**
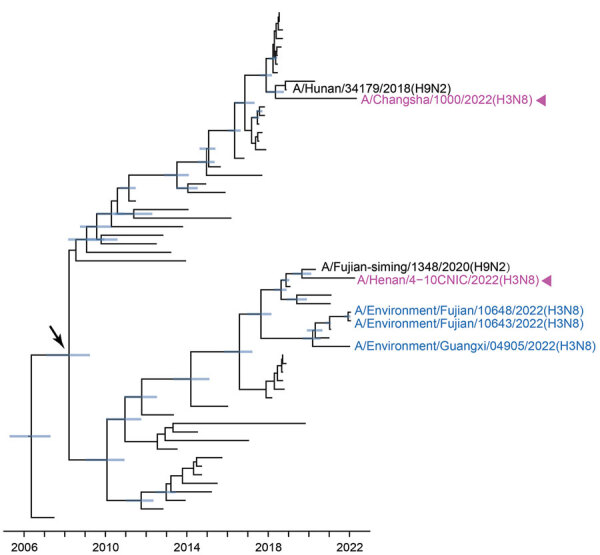
Bayesian time-resolved phylogenetic tree of polymerase basic 1 genes (n = 60) from avian influenza viruses subtype H3 from China and reference sequences from GISAID (https://www.gisaid.org). Violet indicates human H3N8 viruses; blue indicates H3N8 G25 AIVs sequenced in this study; black indicates the closest strains to human H3N8 viruses. Blue node bars correspond to the 95% credible intervals of node heights. Arrow indicates the most recent common ancestor of H3N8 G25 viruses. The fully resolved tree with detailed information is depicted in Appendix Figure 9.

The internal genes of the H3N8 G25 viruses showed earlier tMRCAs than that estimated for HA and NA genes (Appendix Figure 8–13). The internal genes of H3N8 G25 viruses scattered within different subclades without forming a cluster alone. The closest H9N2 viruses to the human H3N8 viruses also differed. For example, the common ancestry of PB1 genes of the H3N8 G25 viruses could be dated back to March 2008 (95% HPD March 2007–May 2009). A/Fujian-siming/1348/2020 (H9N2) was closely related to human H3N8 virus A/Henan/4–10CNIC/2022, and A/Hunan/34179/2018 (H9N2) was close to human H3N8 virus A/Changsha/1000/2022 (Figure 5). Other internal genes of the H3N8 G25 viruses had been estimated to have tMRCAs tracing back to 2010–2018 (Appendix Figure 8–13). Those results further indicate that H3N8 G25 viruses dynamically reassorted with H9N2 viruses.

### Molecular Characterization of the H3 AIVs

We investigated the molecular markers of H3 AIVs in China (Appendix Table 5). One human H3N8 isolate, A/Henan/4-10CNIC/2022, had 228G/S in the receptor binding site, which might alter the binding preference to human-type receptors (*26*). Three H3 AIVs previously sampled from poultry in 2014 had an aspartic acid at position 190, which might alter receptor specificity (*26*).

Key molecular markers associated with increased capacity for receptor binding, viral replication, and pathogenicity in mammals were found in the internal gene segments of avian H3 viruses in China (Appendix Table 5). E627K and E627V in PB2 genes were exclusively detected in human H3N8 viruses, suggesting adaptation of these viruses to mammals. Other mutations such as R389K, I292V, and A588V in PB2, which might be associated with increased polymerase activity and replication in mammalian and avian cells (*27*,*28*) and virulence in mice (*29*), were also found in 2 human isolates and several avian H3 viruses. All H3 AIVs contained N30D, T215A, and P41A in the M1 genes, which might alter the virulence in mice (*30*) and affect growth and transmission in the guinea pig model (*31*).

We identified host signature amino acids in PB2 and PA genes (PB2-702R, PA-356R, PA-409N) (*32*) in human H3N8 isolates and few H3 AIVs, except for A/Changsha/1000/2022, which had PB2-702K (Appendix Table 5, Figure 14). We also analyzed the substitutions related to antiviral drug resistance (Appendix Table 5). Two human H3N8 viruses contained an S31N mutation in the M2 gene, suggesting resistance to amantadine and rimantadine (*33*). In the M2 protein, 26 of 337 H3 AIVs contained drug-resistance mutation V27I/A and 15 contained S31N. Mutations, such as E119V/A/D and H274Y (N2 numbering) were not identified in NA gene, suggesting that all H3 viruses might be sensitive to NA inhibitors (e.g., oseltamivir) (*34*); however, 3 H3 AIVs possessed Q136L, E119G, or H274R, which might affect their drug sensitivity.

## Discussion

The natural reservoir for AIVs is waterfowl; the viruses are spread worldwide by wild bird migration and introduced to domestic poultry across the wild bird–poultry interface (*35*). H3 AIVs have continuously circulated in poultry and wild birds across China (*14*). In China, 4 sublineages (China-1, China-2, China-3, and China-4) of HA genes evolved from the Eurasian lineage and became established in poultry, especially in domestic ducks, after introduction in recent decades. Currently, H3 viruses in China-1 and China-2 sublineages are cocirculating in poultry, with the China-1 sublineage predominating. Although frequent introductions from wild birds to poultry have been observed in other sublineages (e.g., worldwide-1), it is inevitable that continuous introductions will result in new sublineages in poultry (*36*). Our surveillance results also showed that H3N2 predominated among H3 AIVs in poultry-associated environments during 2009–2022. Consistent results for birds were revealed by the available avian strains in GISAID (Appendix Figure 15), although most were collected during 2013–2015 because of strengthened surveillance during the influenza (H7N9) outbreak (*37*–*39*).

Phylogenetic analyses revealed intense reassortment of the H3 AIVs, generating multiple genotypes. On the basis of the sublineage classification, we identified 126 genotypes from 284 H3 AIVs during 2009–2022. Most were transient, and the H3N2 G23 genotype seems to have stabilized in recent years, predominating in southern China. The H3N8 G25 viruses, which had caused human infection, contained complete internal gene cassettes originating from poultry H9N2 ZJ-HJ/07 sublineage, which has persistently circulated in chickens in China and named G57 genotype H9N2 AIVs (*40*). Similar to the pattern of H7N9 AIVs (*41*), H3N8 G25 AIVs might be adapted in chickens rather than ducks.

The H3N8 G25 viruses exhibited distinct tMRCAs among 8 segments. Molecular dating of HA and NA genes of the H3N8 G25 viruses implied that the ancestral virus might have been generated through reassortment between the H3N2 G23 virus and wild bird H3N8 virus before February 2021 (95% HPD October 2020–May 2021). However, the internal genes of the H3N8 G25 viruses showed much earlier tMRCAs than those of HA and NA, indicating that sequential reassortments underlie the emerging of H3N8 G25 viruses.

H3 AIVs have existed for a long time, but to our knowledge, no human infection had been reported until 2022. After reassortment with 6 internal genes of H9N2, current H3N8 AIVs seem to have the advantage of infecting humans (*42*). Ongoing adaptation in mammals after continuous human infections may underlie emergence of pandemic strains. The H3N8 G25 viruses had acquired human-adapted mutations after infecting humans (Appendix Figure 14), such as 228G/S in the HA gene and E627K/V in the PB2 gene, which were also present in 1968 H3N2 pandemic strains (*43*). This finding indicates the pandemic potential of the newly emerged H3N8 AIVs.

For risk assessment of the pandemic potential, human population immunity to a newly emerged animal virus is a critical parameter. HA inhibition assays among poultry workers (*12*) and the general population (*44*) showed seropositivity for the human seasonal H3N2 virus but very low seroprevalence against the newly emerged H3N8 virus. Those results suggest little antigenic cross-reactivity between human seasonal H3N2 virus and the current H3N8 virus and that the human population has little or no preexisting immunity to emerging H3N8 viruses. No drug-resistance mutation to NA inhibitors was observed in H3N8 G25 viruses; therefore, vaccine and drug stockpiles are needed for the potential pandemic preparation.

H3 AIVs have been isolated from asymptomatic ducks (*45*). Recent studies indicate that the newly emerged H3N8 AIVs are pathogenic to chickens (*12*,*46*). Our samples were collected exclusively from avian-linked environments (including LPMs, poultry farms, backyards, and slaughterhouses), according to surveillance guidelines. Thus, we were unable to link the isolated H3 AIVs to specific host information. Poultry sampling might provide helpful information about H3 AIV activity in China. The species of poultry in the LPMs might be confounding factors for the spatiotemporal differences. In this study, the sampling sites were geographically dispersed, and the data were collected from a small number of LPMs. Considering the large number of LPMs in China, especially in rural areas, representativeness of the data might be biased.

AIV surveillance has greatly improved since HPAIV H5N1 infected humans in Hong Kong in 1997 (*47*). However, gaps still exist, and new virus is unpredictable. The AIVs circulating and evolving in poultry might have a preferential ability to transmit to humans directly across the poultry–human interface (*48*). The H3N8 G25 viruses, with increased human receptor binding and low population immunity (*12*), had raised concern for pandemic potential. Dual receptor-binding profiles (*49*,*50*) and mutations associated with enhanced virus replication and pathogenicity in mammals were also found in many H3 AIVs. Surveillance and research of H3 AIVs, as well as the drugs and vaccine capacity, should be strengthened for pandemic preparedness.

Appendix Supplemental information for study of the evolution of avian influenza virus (H3) with spillover into humans, China.
