# Supplementary material for: Evolution of Avian Influenza Virus (H3) with Spillover into Humans, China
Source: Emerg Infect Dis. 2023 Jun;29(6):1191–201. doi: 10.3201/eid2906.221786 (PMC10202863; doi:10.3201/eid2906.221786)
Supplement: Appendix — Supplemental information for study of the evolution of avian influenza virus (H3) with spillover into humans, China. [file 22-1786-Techapp-s1.pdf]

Article DOI: https://doi.org/10.3201/eid2906.221786

*EID cannot ensure accessibility for supplementary materials supplied by authors. Readers who have difficulty accessing supplementary content should contact the authors for assistance.*

# Evolution of Avian Influenza Virus (H3) with Spillover into Humans, China

## Appendix

## Method details

### Environmental sample collection

From January 2009 through June 2022, environmental samples were collected from avian-linked environments (including live poultry markets, poultry farms, backyards, slaughterhouses, and wild bird habitats) monthly across 31 provinces in Chinese mainland according to avian influenza virus (AIV) surveillance guideline of Chinese Center for Disease Control and Prevention (CDC). The environmental samples included poultry feces, sewage, poultry drinking water, surface swabs from poultry cages and chopping boards, etc. Feces or swab samples were maintained in viral transport medium; for liquid samples (sewage, poultry drinking water), 5-ml liquid was collected. The samples were sent to local Chinese National Influenza Surveillance Network laboratories within 48 h and stored at 4°C.

All samples were centrifuged at 3,000 rpm for 10 min, and the supernatant of each sample was harvested for further test. Influenza A viruses were identified by real-time reverse transcription-PCR (real-time RT-PCR). Of these, positive samples were sent to Chinese National Influenza Center (CNIC) for virus isolation.

**Virus isolation and identification**

Viruses were isolated in 9 to 11-day specific-pathogen-free (SPF) embryonated chicken eggs. After incubation for 48–72 hours at 37°C, the presence of the virus in the allantoic fluids of eggs was identified by a hemagglutination test using 1% turkey red blood cells (TRBC).

**RNA extraction and genome sequencing**

Virus RNA was extracted from the isolated viruses using the MagMAX CORE Nucleic Acid Purification Kit (Thermo Fisher, Waltham, MA). The extracted RNA was subjected to reverse transcription and amplification using the SuperScript® III One-Step RT-PCR system (Thermo Fisher, Waltham, MA) according to the described method (*1*).Whole genome sequencing of influenza A virus was implemented on the automatic Applied Biosystems 3730xl DNA Analyzer (Life Technologies, USA) or MiSeq high-throughput sequencing platform (Illumina, Inc., San Diego, CA, USA). The raw data from MiSeq platform were paired reads with length of 150 bp. Low-quality reads were trimmed, and the filtered reads were sampled and de novo-assembled using Velvet (version 1.2.10) (*2*) and Newbler (version 2.5). Contigs were blasted against a database containing all influenza A virus nucleotide sequences collected from the National Center for Biotechnology Information (www.ncbi.nlm.nih.gov/genomes/FLU) and the Global Initiative on Sharing All Influenza Data (GISAID) (http://www.gisaid.org). Sequences with the highest similarity were selected as references for mapping of reads using Bowtie 2 (version 2.1.0) (*3*). The influenza A virus genome sequences were obtained by extracting the consensus sequences from the mapping results, with a coverage depth of at least 30 times at each site on the eight segments.

**Sequence collection and alignment**

All the sequences of 188 environmental H3 isolates were initially obtained in our surveillance (Appendix Table 1) and 32 of them have been published in our previous study (*4*). H3, N2, N3, N6, N8 and internal gene sequences of avian-origin viruses were downloaded from the GISAID EpiFlu database as of June 25, 2022. Sequences of two human H3N8 viruses isolated in Henan and Hunan provinces were also included. The resulting sequences of each segment were aligned using MAFFT software v7.222 (*5*) and manually adjusted to correct frameshift errors in MEGA v7.0 (*6*). The coding region of each segment was retained and the signal peptide was removed from the HA segment. Sequences with ≥97% of the length of coding region and ≤3 degenerate bases were included for molecular characterization and phylogenetic analyses. To comprehensively elaborate the evolution of H3 AIVs in China, we extended the selection of Chinese sequences. The sequences collected in China were retained with less than 4 degenerate bases and no less than 90% of the length of coding region of the segment. For the HA gene, sequences with complete HA1 domain were also included.

**Phylogenetic analyses**

Maximum likelihood (ML) phylogenies of all segments were reconstructed using the FastTree software v2.1.11 (*7*) under the GTRGAMMA model with 1,000 bootstrap replicates. All H3-HA gene sequences were included in the phylogenetic analyses. The datasets of NA genes and internal genes were reduced using cd-hit to improve the computational efficiency. All H3 virus strains isolated from avian host and environment samples in China (Figure 1; Appendix Figure 15) as well as two human H3N8 virus strains were retained for reconstructing evolutionary relationships. We also retrieved the sequences that shared the highest similarity with Chinese H3 AIVs by BLAST. The resulting trees were classified into divergent lineages or sublineages according to the topology of phylogenetic trees with bootstrap support values ≥ 70%. Clusters from

Eurasian wild bird gene pool were then manually merged, if necessary, based on the bootstrap support values and reported classification (*8*). The ggtree package in R(*9*) and FigTree v1.4.4 (http://tree.bio.ed.ac.uk/software/figtree/) were used for visualization and annotation.

**Genotypic analysis**

There is remarkably frequent occurrence of reassortment in AIV, that formed the wild bird gene pool. Studies on large number of sequences provided little evidence for the elevated fitness of specific gene combinations in AIV isolates from wild birds (*10*). AIVs maintained in wild birds spill over into novel hosts including domestic gallinaceous poultry, horses, swine, and humans leading to the emergence of lineages/sublineages transmissible in the new host. Hence, we classified NA and internal genes into Eurasian wild bird gene pool, ZJ-5 sublineage (of the wild bird viruses) (*8*), poultry H5N1/H5N6 sublineage, poultry H9N2 ZJ-HJ/07 sublineage, and/or waterfowl H6 sublineage. For strains with all eight gene segments available, their genotypes were determined by the combination of lineage/sublineage assignments of each of eight segments. Virus genotypes were analyzed for H3 viruses isolated from human, avian and environmental samples in China during 2009-2022. For each NA subtype, genotypes were named G1 to Gn, according to the time of identification.

**Molecular dating**

To estimate the time to the most recent common ancestor (tMRCA) of human H3N8 viruses, smaller datasets for eight gene segments containing human H3N8 virus strains were selected to run time-measured Bayesian Markov chain Monte Carlo (MCMC) analysis implemented in BEAST v1.10.4 (*11*). Root-to-tip genetic divergence against sampling dates was analyzed using TempEst v1.5.3 (*12*) to investigate the temporal signal of our datasets. The best-fitting nucleotide substitution models (GTR+G

for the MP gene, and GTR+G+I for other gene segments) were identified according to the Corrected Akaike Information Criterion in MEGA v7.0 (*6*). A relaxed clock model with uncorrelated lognormal distribution was used. A Bayesian skyride model with time-aware smoothing was used (*13*). The default distribution was used for prior CP1 + 2, CP3, and ucld.mean. Bayesian MCMC analyses were run for 50 million steps, with sampling every 5,000 steps. Multiple independent MCMC trajectories were computed and combined. The first 10% states were removed. Final effective sample size as assessed in Tracer v1.7.2. were >200 for all parameters. Maximum clade credibility (MCC) trees with median node height were generated using TreeAnnotator v1.10.4. We visualised and annotated the MCC trees using ggtree (*9*).

## Evolution details of H3 AIVs

### Evolution of China-2 sublineage

After the isolation of H3N2 in Jiangsu in 2004, the China-2 sublineage evolved into 3 subgroups (Figure 2; Appendix Figure 1). The China-2.1 subgroup had a large proportion of viruses isolated from China during 2009-2018. The China-2.2 subgroup mainly comprised environmental H3 viruses (29/31,93.5%) sequenced in this study during 2015-2021. Poultry-origin H3N2 and H3N8 viruses found in Guangxi during 2009-2013 independently formed the China-2.3 subgroup.

### Evolution of China-3 and China-4 sublineages

A few isolates in this study belonged to the sublineages China-3 (15) and China-4 (7), detected during 2009-2018 (Figure 2; Appendix Figure 1). Most of the viruses in the China-3 sublineage were collected from poultry within the same sampling event and location.

### Evolution of other H3 sublineages

Chinese H3 AIVs were also found scattered in sublineages Asia, Europe-Asia, worldwide-1, and worldwide-2 (Figure 2; Appendix Figure 1). Both poultry and wild bird isolates were found in each sublineage. The worldwide-1 sublineage contained 38 H3 virus strains collected from 1999 through 2022 in China, as well as other H3 viruses covering Asia, Europe, and North America. H3 viruses combined with N1-N8 subtypes were detected, among which H3N8 was predominant. Most of the Chinese H3 strains (31/38) in this sublineage were sampled from poultry or poultry-related environments. The worldwide-2 sublineage contained a smaller cluster of viruses including H3N2, H3N3, H3N5, H3N6, and H3N8 subtypes, spreading across the Eurasian and North American continents. Three H3N2 AIVs in 2011 and 2 H3N3 AIVs in 2000 from China belonged to this sublineage. The viruses in the Europe-Asia sublineage were mainly distributed in Europe and Asia, with a minority (6/230, 2.6%) in Africa and North America. N1, N3, N5, N6, and N8 subtypes were identified. Sixteen H3 strains from poultry and wild birds in China fell into this sublineage, with a transient detection in 4 years. Viruses in the Asia sublineage were mainly sampled from Asian countries except for 7 viruses collected from Russia. H3 viruses with various NA subtypes including N1, N2, N3, N5, N6, N8, and N9 were identified. Fifteen H3 isolates from China belonged to the Asia sublineage.

## Dynamic reassortment of H3 viruses

### Reassortment with N2 genes

The N2 genes of AIVs in the Eurasian lineage could be further classified into 4 major sublineages: Eurasian-1, Eurasian-2, waterfowl H6N2, and poultry H9N2 sublineages (Appendix Figure 2A). The majority of H3N2 viruses in this study were clustered in the Eurasian-2. The N2 genes of this sublineage have circulated in the

poultry and poultry-related environment in China with the earliest isolate detected in 2009. The H3N2 AIVs in China were closely related to the viruses isolated from various species with multiple subtypes, such as H4, H5, H9, etc. Notably, NA genes of 6 H3N2 strains isolated from domestic ducks during 2013-2014 were clustered with chicken H9N2 viruses.

### Reassortment with N3 genes

The NA genes of the H3N3 AIVs in China were all derived from the Eurasian lineage (Appendix Figure 2B). Notably, one H3N3 strain sequenced in our study, A/Environment/Hunan/13561/2020, showed a close relationship with the NA genes of human-origin H10N3 virus and H10N3 viruses circulating in chicken during 2019-2021. Another H3N3 strain sampled in 2020 was grouped with duck H7N3 viruses circulating in China during 2010-2018.

### Reassortment with N6 genes

The NA genes of the H3N6 AIVs in China were scattered in the Eurasian lineage (Appendix Figure 2C). H3N6 viruses isolated from poultry and wild bird in Jiangxi, Guangdong, Guangxi, and Hunan provinces during 2014-2015 were clustered with highly pathogenic AIV (HPAIV) H5N6.

### Reassortment with internal genes

To explore the pattern of reassortment of H3 AIVs in China, we reconstructed the phylogenies for all six internal genes (Appendix Figure 3). The internal genes of all the H3 AIVs sequenced through our surveillance belonged to the Eurasian lineage, except for the M gene of A/Environment/Sichuan/32281/2016(H3N2), as previously described (*4*). Except for two H3N8 AIVs, almost all the H3 AIVs in China had internal genes derived from the Eurasian wild bird gene pool, ZJ-5 sublineage (of the wild bird viruses), poultry

H5N1/H5N6 sublineage, poultry H9N2 ZJ-HJ/07 sublineage, and/or waterfowl H6 sublineage.

A large proportion of H3 AIVs in China fell into the Eurasian wild bird reservoir (Appendix Figure 3). Many H3 AIVs contained internal genes (especially PA, NP and, M genes) from the ZJ-5 sublineage, of the wild bird viral gene pool, which consisted mainly of viruses isolated from domestic waterfowl in China. Very few H3 AIVs derived their internal genes (PB2 (5), PB1 (1), PA (2) and M (1)) from the waterfowl H6 sublineage. A small proportion of H3 AIVs were grouped into the poultry-established lineages. In the poultry H5N1/H5N6 sublineage, 2-6 H3 AIVs isolated in 2013-2015 were found, and one was isolated in 2004 with NP gene.

**Emerging of multiple genotypes**

Seventy-three genotypes (G1-G73) were identified from 212 H3N2 viruses, most of which were transient genotypes (52/73,71.2%) (Appendix Figure 4A). Thirteen genotypes were sporadically detected in 2-3 years while eight genotypes (G6, G23-G25, G39, G54, and G56) were detected in more than 3 years. Obviously, the number of viral genotypes went up and down with the number of viruses detected. The number of genotypes increased gradually from 2009 through 2013, and tend to stabilize during 2014-2019. After 2019, only a few genotypes were detected. Most of the genotypes were found in southern China, particularly in Guangdong and Guangxi provinces which detected twenty-five and twenty-three genotypes, respectively (Appendix Figure 5A). Of the eight common genotypes, G6, G23, and G25 have been circulating for many years and spread to more regions (Appendix Figure 4A and 5A). Notably, the G23, which had been detected as early as 2014, continued circulating and was monitored in 2022. The H3N2 G23 viruses contained HA genes from the China-1 sublineage, NA genes from the Eurasian lineage, M genes from the ZJ-5 sublineage, and other 5 internal genes from the Eurasian wild bird gene pool (Appendix Table 4).

Despite a low reported detection, considerable genomic diversity was identified in H3N3, H3N6, and H3N8 AIVs. Eleven genotypes (G1-G11) from 14 H3N3 genomes, seventeen (G1-G17) from 25 H3N6, and twenty-five (G1-G25) from 33 H3N8 were found (Appendix Figure 4B-D). Most of the strains with identical genotypes were from the same year and location (Appendix Table 4). Only four genotypes, including G7 of H3N3, G2, G4, and G6 of H3N8, have been detected in more than one year (Appendix Figure 4B and 4D) and the former three were found in different provinces (Appendix Figure 5B and 5D).

## References


1. Zhou B, Donnelly ME, Scholes DT, St George K, Hatta M, Kawaoka Y, et al. Single-reaction genomic amplification accelerates sequencing and vaccine production for classical and Swine origin human influenza a viruses. J Virol. 2009;83:10309–13. PubMed https://doi.org/10.1128/JVI.01109-09

2. Zerbino DR, Birney E. Velvet: algorithms for de novo short read assembly using de Bruijn graphs. Genome Res. 2008;18:821–9. PubMed https://doi.org/10.1101/gr.074492.107

3. Langmead B, Salzberg SL. Fast gapped-read alignment with Bowtie 2. Nat Methods. 2012;9:357–9. PubMed https://doi.org/10.1038/nmeth.1923

4. Zou S, Tang J, Zhang Y, Liu L, Li X, Meng Y, et al. Molecular characterization of H3 subtype avian influenza viruses based on poultry-related environmental surveillance in China between 2014 and 2017. Virology. 2020;542:8–19. PubMed https://doi.org/10.1016/j.virol.2020.01.003

5. Katoh K, Standley DM. MAFFT multiple sequence alignment software version 7: improvements in performance and usability. Mol Biol Evol. 2013;30:772–80. PubMed https://doi.org/10.1093/molbev/mst010

6. Kumar S, Stecher G, Tamura K. MEGA7: Molecular Evolutionary Genetics Analysis Version 7.0 for Bigger Datasets. Mol Biol Evol. 2016;33:1870–4. PubMed https://doi.org/10.1093/molbev/msw054

7. Price MN, Dehal PS, Arkin AP. FastTree 2—approximately maximum-likelihood trees for large alignments. PLoS One. 2010;5:e9490. PubMed https://doi.org/10.1371/journal.pone.0009490

8. Lam TT, Wang J, Shen Y, Zhou B, Duan L, Cheung CL, et al. The genesis and source of the H7N9 influenza viruses causing human infections in China. Nature. 2013;502:241–4. PubMed https://doi.org/10.1038/nature12515

9. Yu GC, Smith DK, Zhu HC, Guan Y, Lam TTY. GGTREE: an R package for visualization and annotation of phylogenetic trees with their covariates and other associated data. Methods Ecol Evol. 2017;8:28–36. https://doi.org/10.1111/2041-210X.12628

10. Dugan VG, Chen R, Spiro DJ, Sengamalay N, Zaborsky J, Ghedin E, et al. The evolutionary genetics and emergence of avian influenza viruses in wild birds. PLoS Pathog. 2008;4:e1000076. PubMed https://doi.org/10.1371/journal.ppat.1000076

11. Suchard MA, Lemey P, Baele G, Ayres DL, Drummond AJ, Rambaut A. Bayesian phylogenetic and phylodynamic data integration using BEAST 1.10. Virus Evol. 2018;4:vey016. PubMed https://doi.org/10.1093/ve/vey016

12. Rambaut A, Lam TT, Max Carvalho L, Pybus OG. Exploring the temporal structure of heterochronous sequences using TempEst (formerly Path-O-Gen). Virus Evol. 2016;2:vew007. PubMed https://doi.org/10.1093/ve/vew007

13. Minin VN, Bloomquist EW, Suchard MA. Smooth skyride through a rough skyline: Bayesian coalescent-based inference of population dynamics. Mol Biol Evol. 2008;25:1459–71. PubMed https://doi.org/10.1093/molbev/msn090

**Appendix Table 1.** Detailed information of 188 environmental H3 subtype viruses obtained in our surveillance.

| Subtype | Isolate Id | Isolate name* | Province | Collection Date | Sample Type | Sample Site† | Accession numbers‡ |
|---|---|---|---|---|---|---|---|
| H3N2 | EPI_ISL_15720733 | A/Environment/Guangdong/03/09 | Guangdong | 2009-04-03 | surface swab of cages | D | EPI2210289~EPI2210296 |
| | EPI_ISL_15720738 | A/Environment/Guangxi/03045/2012 | Guangxi | 2011-07-19 | sewage | A | EPI2210321~EPI2210328 |
| | EPI_ISL_15720739 | A/Environment/Guangxi/03056/2012 | Guangxi | 2012-06-22 | sewage | A | EPI2210329~EPI2210336 |
| | EPI_ISL_15720740 | A/Environment/Anhui/03012/2012 | Anhui | 2012-09-11 | sewage | A | EPI2210337~EPI2210344 |
| | EPI_ISL_15720741 | A/Environment/Guangxi/03047/2012 | Guangxi | 2012-09-28 | sewage | A | EPI2210345~EPI2210352 |
| | EPI_ISL_15720742 | A/Environment/Guangxi/03059/2012 | Guangxi | 2012-10-11 | feces | A | EPI2210353~EPI2210360 |
| | EPI_ISL_15720743 | A/Environment/Guangxi/03058/2012 | Guangxi | 2012-10-11 | sewage | A | EPI2210361~EPI2210368 |
| | EPI_ISL_15720744 | A/Environment/Guangxi/06232/2013 | Guangxi | 2013-07-12 | others | A | EPI2210369~EPI2210376 |
| | EPI_ISL_15720745 | A/Environment/Hunan/26068/2014 | Hunan | 2014-03-27 | feces | A | EPI2210377~EPI2210384 |
| | EPI_ISL_15720746 | A/Environment/Hunan/27451/2014 | Hunan | 2014-04-09 | sewage | D | EPI2210385~EPI2210392 |
| | EPI_ISL_15720747 | A/Environment/Guangxi/28750/2014 | Guangxi | 2014-04-14 | surface swab of chopping boards | A | EPI2210393~EPI2210400 |
| | EPI_ISL_15720748 | A/Environment/Guangdong/77214/2014 | Guangdong | 2014-08-18 | surface swab of cages | A | EPI2210401~EPI2210408 |
| | EPI_ISL_15720749 | A/Environment/Hunan/02458/2014 | Hunan | 2014-12-15 | drinking water | A | EPI2210409~EPI2210416 |
| | EPI_ISL_15720750 | A/Environment/Chongqing/45115/2015 | Chongqing | 2015-01-20 | surface swab of chopping boards | A | EPI2210417~EPI2210424 |
| | EPI_ISL_15720751 | A/Environment/Guangxi/32186/2015 | Guangxi | 2015-01-21 | feces | A | EPI2210425~EPI2210432 |
| | EPI_ISL_15720752 | A/Environment/Guangxi/41272/2015 | Guangxi | 2015-01-28 | drinking water | A | EPI2210433~EPI2210440 |
| | EPI_ISL_15720753 | A/Environment/Jiangxi/33299/2015 | Jiangxi | 2015-03-24 | surface swab of cages | A | EPI2210441~EPI2210448 |
| | EPI_ISL_15720754 | A/Environment/Hunan/37943/2015 | Hunan | 2015-04-09 | drinking water | A | EPI2210449~EPI2210456 |
| | EPI_ISL_15720755 | A/Environment/Hunan/34583/2015 | Hunan | 2015-04-10 | sewage | A | EPI2210457~EPI2210464 |
| | EPI_ISL_15720756 | A/Environment/Hunan/42907/2015 | Hunan | 2015-08-10 | drinking water | C | EPI2210465~EPI2210472 |
| | EPI_ISL_15723330 | A/EV/SD/226214/2015 | Shandong | 2015-10-15 | unknown | A | EPI2210473~EPI2210480 |
| | EPI_ISL_15723331 | A/EV/SD/226238/2015 | Shandong | 2015-10-21 | unknown | A | EPI2210481~EPI2210488 |
| | EPI_ISL_15723332 | A/EV/SD/226236/2015 | Shandong | 2015-10-21 | unknown | A | EPI2210489~EPI2210496 |
| | EPI_ISL_15723333 | A/Environment/Guangdong/15077/2015 | Guangdong | 2015-11-16 | feces | A | EPI2210497~EPI2210504 |
| | EPI_ISL_15723334 | A/Environment/Yunnan/01456/2015 | Yunnan | 2015-11-27 | feces | D | EPI2210505~EPI2210512 |
| | EPI_ISL_15723335 | A/Environment/Guangdong/15131/2015 | Guangdong | 2015-12-01 | feces | A | EPI2210513~EPI2210520 |
| | EPI_ISL_15723337 | A/Environment/Hunan/02861/2015 | Hunan | 2015-12-15 | sewage | A | EPI2210529~EPI2210536 |
| | EPI_ISL_15723338 | A/Environment/Hunan/10170/2016 | Hunan | 2016-01-06 | drinking water | A | EPI2210537~EPI2210544 |
| | EPI_ISL_15723339 | A/Environment/Hunan/11041/2016 | Hunan | 2016-01-18 | drinking water | A | EPI2210545~EPI2210552 |
| | EPI_ISL_15723340 | A/Environment/Sichuan/32289/2016 | Sichuan | 2016-02-04 | feces | A | EPI2210553~EPI2210560 |
| | EPI_ISL_15723341 | A/Environment/Hunan/31561/2016 | Hunan | 2016-03-22 | feces | A | EPI2210561~EPI2210568 |
| | EPI_ISL_15723342 | A/Environment/Sichuan/48404/2016 | Sichuan | 2016-06-27 | feces | A | EPI2210569~EPI2210576 |
| | EPI_ISL_15723343 | A/Environment/Jiangsu/06688/2016 | Jiangsu | 2016-12-21 | surface swab of chopping boards | A | *EPI2210577~EPI2210583* |
| | EPI_ISL_15723344 | A/Environment/Jiangsu/06689/2016 | Jiangsu | 2016-12-21 | sewage | A | EPI2210584~EPI2210591 |
| | EPI_ISL_15723345 | A/Environment/Hubei/33702/2017 | Hubei | 2017-02-09 | surface swab of cages | A | EPI2210592~EPI2210599 |
| | EPI_ISL_15723346 | A/Environment/Guangxi/23533/2017 | Guangxi | 2017-03-30 | surface swab of cages | A | EPI2210600~EPI2210607 |
| | EPI_ISL_15723347 | A/Environment/Guangxi/23558/2017 | Guangxi | 2017-04-05 | drinking water | A | EPI2210608~EPI2210615 |
| | EPI_ISL_15723348 | A/Environment/Hunan/34012/2017 | Hunan | 2017-04-07 | sewage | A | EPI2210616~EPI2210623 |
| | EPI_ISL_15723349 | A/Environment/Hunan/25648/2017 | Hunan | 2017-04-11 | surface swab of cages | A | EPI2210624~EPI2210631 |
| | EPI_ISL_15723350 | A/Environment/Hunan/25643/2017 | Hunan | 2017-04-11 | feces | A | EPI2210632~EPI2210639 |

| Subtype | Isolate Id | Isolate name* | Province | Collection Date | Sample Type | Sample Site† | Accession numbers‡ |
|---|---|---|---|---|---|---|---|
| | EPI_ISL_15723351 | A/Environment/Guangxi/32152/2017 | Guangxi | 2017-05-09 | sewage | A | EPI2210640~EPI2210647 |
| | EPI_ISL_15723352 | A/Environment/Guangxi/32060/2017 | Guangxi | 2017-05-22 | drinking water | A | EPI2210648~EPI2210655 |
| | EPI_ISL_15723353 | A/Environment/Guangdong/35458/2017 | Guangdong | 2017-05-31 | drinking water | A | EPI2210656~EPI2210663 |
| | EPI_ISL_15723354 | A/Environment/Fujian/36985/2017 | Fujian | 2017-07-07 | sewage | A | EPI2210664~EPI2210671 |
| | EPI_ISL_15723476 | A/Environment/Chongqing/36535/2017 | Chongqing | 2017-08-17 | surface swab of cages | A | EPI2210672~EPI2210679 |
| | EPI_ISL_15723477 | A/Environment/Guangxi/40947/2017 | Guangxi | 2017-11-22 | feces | A | EPI2210680~EPI2210687 |
| | EPI_ISL_15723478 | A/Environment/Guangxi/40934/2017 | Guangxi | 2017-11-22 | feces | A | EPI2210688~EPI2210695 |
| | EPI_ISL_15723479 | A/Environment/Guangxi/05048/2017 | Guangxi | 2017-11-28 | others | A | EPI2210696~EPI2210703 |
| | EPI_ISL_15723480 | A/Environment/Fujian/04958/2017 | Fujian | 2017-12-18 | feces | A | EPI2210704~EPI2210711 |
| | EPI_ISL_15723481 | A/Environment/Guangxi/24894/2018 | Guangxi | 2018-01-17 | surface swab of chopping boards | A | EPI2210712~EPI2210719 |
| | EPI_ISL_15723482 | A/Environment/Guangxi/24886/2018 | Guangxi | 2018-01-17 | surface swab of chopping boards | A | EPI2210720~EPI2210727 |
| | EPI_ISL_15723484 | A/Environment/Guangxi/24775/2018 | Guangxi | 2018-02-03 | surface swab of cages | A | EPI2210736~EPI2210743 |
| | EPI_ISL_15723485 | A/Environment/Guangdong/27940/2018 | Guangdong | 2018-02-06 | feces | A | EPI2210744~EPI2210751 |
| | EPI_ISL_15723486 | A/Environment/Guangxi/24790/2018 | Guangxi | 2018-02-08 | feces | A | EPI2210752~EPI2210759 |
| | EPI_ISL_15723487 | A/Environment/Guangxi/24805/2018 | Guangxi | 2018-02-09 | drinking water | A | EPI2210760~EPI2210767 |
| | EPI_ISL_15723488 | A/Environment/Guangxi/24803/2018 | Guangxi | 2018-02-09 | sewage | A | EPI2210768~EPI2210775 |
| | EPI_ISL_15723489 | A/Environment/Guangxi/28076/2018 | Guangxi | 2018-02-27 | feces | A | EPI2210776~EPI2210783 |
| | EPI_ISL_15723490 | A/Environment/Guangdong/30984/2018 | Guangdong | 2018-04-09 | feces | A | EPI2210784~EPI2210791 |
| | EPI_ISL_15723491 | A/Environment/Hunan/28854/2018 | Hunan | 2018-04-16 | feces | A | EPI2210792~EPI2210799 |
| | EPI_ISL_15723492 | A/Environment/Guangdong/30702/2018 | Guangdong | 2018-05-23 | surface swab of cages | A | EPI2210800~EPI2210807 |
| | EPI_ISL_15723493 | A/Environment/Zhejiang/31298/2018 | Zhejiang | 2018-05-23 | feces | A | EPI2210808~EPI2210815 |
| | EPI_ISL_15723494 | A/Environment/Zhejiang/31299/2018 | Zhejiang | 2018-05-23 | feces | A | EPI2210816~EPI2210823 |
| | EPI_ISL_15723495 | A/Environment/Guangxi/32515/2018 | Guangxi | 2018-08-21 | feces | A | EPI2210824~EPI2210831 |
| | EPI_ISL_15723496 | A/Environment/Guangxi/32510/2018 | Guangxi | 2018-08-21 | surface swab of cages | A | EPI2210832~EPI2210839 |
| | EPI_ISL_15723497 | A/Environment/Guangxi/32507/2018 | Guangxi | 2018-08-21 | feces | A | EPI2210840~EPI2210847 |
| | EPI_ISL_15723498 | A/Environment/Guangdong/32693/2018 | Guangdong | 2018-09-10 | others | A | EPI2210848~EPI2210855 |
| | EPI_ISL_15723499 | A/Environment/Chongqing/33939/2018 | Chongqing | 2018-09-13 | feces | A | EPI2210856~EPI2210863 |
| | EPI_ISL_15723500 | A/Environment/Chongqing/33926/2018 | Chongqing | 2018-09-17 | feces | A | EPI2210864~EPI2210871 |
| | EPI_ISL_15723501 | A/Environment/Guangxi/32937/2018 | Guangxi | 2018-09-25 | surface swab of chopping boards | A | EPI2210872~EPI2210879 |
| | EPI_ISL_15723502 | A/Environment/Guangxi/32906/2018 | Guangxi | 2018-09-27 | others | A | EPI2210880~EPI2210887 |
| | EPI_ISL_15723503 | A/Environment/Guangxi/32562/2018 | Guangxi | 2018-09-28 | feces | A | EPI2210888~EPI2210895 |
| | EPI_ISL_15723504 | A/Environment/Guangxi/33582/2018 | Guangxi | 2018-10-28 | surface swab of cages | A | EPI2210896~EPI2210903 |
| | EPI_ISL_15723506 | A/Environment/Guangdong/34268/2018 | Guangdong | 2018-11-06 | feces | A | EPI2210912~EPI2210919 |
| | EPI_ISL_15723507 | A/Environment/Guangxi/33095/2018 | Guangxi | 2018-11-07 | sewage | A | EPI2210920~EPI2210927 |
| | EPI_ISL_15723508 | A/Environment/Guangxi/33056/2018 | Guangxi | 2018-11-07 | sewage | A | EPI2210928~EPI2210935 |
| | EPI_ISL_15723509 | A/Environment/Hunan/04235/2018 | Hunan | 2018-11-12 | feces | A | EPI2210936~EPI2210943 |
| | EPI_ISL_15723510 | A/Environment/Hunan/05861/2018 | Hunan | 2018-11-29 | feces | A | EPI2210944~EPI2210951 |
| | EPI_ISL_15723511 | A/Environment/Guangdong/27250/2018 | Guangdong | 2018-12-03 | sewage | D | EPI2210952~EPI2210959 |
| | EPI_ISL_15723512 | A/Environment/Guangdong/27249/2018 | Guangdong | 2018-12-03 | surface swab of chopping boards | D | EPI2210960~EPI2210967 |
| | EPI_ISL_15723522 | A/Environment/Guangxi/34661/2018 | Guangxi | 2018-12-03 | drinking water | A | EPI2210968~EPI2210975 |

| Subtype | Isolate Id | Isolate name* | Province | Collection Date | Sample Type | Sample Site† | Accession numbers‡ |
|---|---|---|---|---|---|---|---|
| | EPI_ISL_15723523 | A/Environment/Guangxi/35164/2018 | Guangxi | 2018-12-07 | sewage | A | EPI2210976~EPI2210983 |
| | EPI_ISL_15723524 | A/Environment/Zhejiang/02021/2018 | Zhejiang | 2018-12-17 | surface swab of chopping boards | A | EPI2210984~EPI2210991 |
| | EPI_ISL_15723525 | A/Environment/Guangxi/01878/2018 | Guangxi | 2018-12-20 | others | A | EPI2210992~EPI2210999 |
| | EPI_ISL_15723526 | A/Environment/Guangxi/01869/2018 | Guangxi | 2018-12-20 | feces | A | EPI2211000~EPI2211007 |
| | EPI_ISL_15723527 | A/Environment/Guangdong/14452/2018 | Guangdong | 2018-12-24 | surface swab of cages | A | EPI2211008~EPI2211015 |
| | EPI_ISL_15723528 | A/Environment/Guangdong/14440/2018 | Guangdong | 2018-12-24 | surface swab of cages | A | EPI2211016~EPI2211023 |
| | EPI_ISL_15723529 | A/Environment/Guangdong/14438/2018 | Guangdong | 2018-12-24 | feces | A | EPI2211024~EPI2211031 |
| | EPI_ISL_15723530 | A/Environment/Guangdong/14430/2018 | Guangdong | 2018-12-24 | sewage | A | EPI2211032~EPI2211039 |
| | EPI_ISL_15723531 | A/Environment/Guangdong/14334/2018 | Guangdong | 2018-12-24 | surface swab of cages | A | EPI2211040~EPI2211047 |
| | EPI_ISL_15723532 | A/Environment/Guangdong/27641/2018 | Guangdong | 2018-12-26 | others | A | EPI2211048~EPI2211055 |
| | EPI_ISL_15723533 | A/Environment/Guangdong/27729/2018 | Guangdong | 2018-12-26 | feces | A | EPI2211056~EPI2211063 |
| | EPI_ISL_15723534 | A/Environment/Guangdong/27727/2018 | Guangdong | 2018-12-26 | feces | A | EPI2211064~EPI2211071 |
| | EPI_ISL_15723535 | A/Environment/Guangxi/05712/2018 | Guangxi | 2018-12-26 | feces | A | EPI2211072~EPI2211079 |
| | EPI_ISL_15723893 | A/Environment/Guangxi/01886/2018 | Guangxi | 2018-12-26 | others | A | EPI2211080~EPI2211087 |
| | EPI_ISL_15723894 | A/Environment/Guangxi/01885/2018 | Guangxi | 2018-12-26 | sewage | A | EPI2211088~EPI2211095 |
| | EPI_ISL_15723895 | A/Environment/Guangdong/27245/2019 | Guangdong | 2019-01-03 | sewage | A | EPI2211096~EPI2211103 |
| | EPI_ISL_15723896 | A/Environment/Guangdong/27246/2019 | Guangdong | 2019-01-08 | surface swab of chopping boards | A | EPI2211104~EPI2211111 |
| | EPI_ISL_15723897 | A/Environment/Guangxi/09150/2019 | Guangxi | 2019-01-17 | sewage | A | EPI2211112~EPI2211119 |
| | EPI_ISL_15723898 | A/Environment/Hubei/43318/2019 | Hubei | 2019-01-29 | inner wall and outer surface of defeathering machine | C | EPI2211120~EPI2211127 |
| | EPI_ISL_15723899 | A/Environment/Guangdong/34622/2019 | Guangdong | 2019-02-03 | drinking water | A | EPI2211128~EPI2211135 |
| | EPI_ISL_15723900 | A/Environment/Guangdong/34240/2019 | Guangdong | 2019-02-13 | feces | A | EPI2211136~EPI2211143 |
| | EPI_ISL_15723902 | A/Environment/Hunan/40453/2019 | Hunan | 2019-03-18 | sewage | A | EPI2211152~EPI2211159 |
| | EPI_ISL_15723903 | A/Environment/Hunan/40442/2019 | Hunan | 2019-03-18 | feces | A | EPI2211160~EPI2211167 |
| | EPI_ISL_15723904 | A/Environment/Guangxi/25038/2019 | Guangxi | 2019-03-25 | drinking water | A | EPI2211168~EPI2211175 |
| | EPI_ISL_15723905 | A/Environment/Gansu/01469/2019 | Gansu | 2019-03-27 | surface swab of cages | A | EPI2211176~EPI2211183 |
| | EPI_ISL_15723906 | A/Environment/Guangxi/32383/2019 | Guangxi | 2019-04-30 | sewage | A | EPI2211184~EPI2211191 |
| | EPI_ISL_15723907 | A/Environment/Guangxi/39142/2019 | Guangxi | 2019-05-31 | others | D | EPI2211192~EPI2211199 |
| | EPI_ISL_15723908 | A/Environment/Fujian/39244/2019 | Fujian | 2019-06-05 | feces | A | EPI2211200~EPI2211207 |
| | EPI_ISL_15723909 | A/Environment/Guangxi/45278/2019 | Guangxi | 2019-06-12 | surface swab of cages | D | EPI2211208~EPI2211215 |
| | EPI_ISL_15723910 | A/Environment/Hubei/43399/2019 | Hubei | 2019-06-20 | surface swab of cages | D | EPI2211216~EPI2211223 |
| | EPI_ISL_15723911 | A/Environment/Guangxi/39550/2019 | Guangxi | 2019-07-18 | drinking water | D | EPI2211224~EPI2211231 |
| | EPI_ISL_15723912 | A/Environment/Guangxi/39500/2019 | Guangxi | 2019-08-12 | feces | D | EPI2211232~EPI2211239 |
| | EPI_ISL_15723913 | A/Environment/Guangxi/41094/2019 | Guangxi | 2019-09-18 | sewage | D | EPI2211240~EPI2211247 |
| | EPI_ISL_15723914 | A/Environment/Chongqing/41153/2019 | Chongqing | 2019-10-18 | feces | D | EPI2211248~EPI2211255 |
| | EPI_ISL_15723915 | A/Environment/Guangxi/45299/2019 | Guangxi | 2019-10-23 | surface swab of cages | D | EPI2211256~EPI2211263 |
| | EPI_ISL_15723916 | A/Environment/Guangdong/09210/2019 | Guangdong | 2019-11-13 | cloacal swab | A | EPI2211264~EPI2211271 |
| | EPI_ISL_15723917 | A/Environment/Guangdong/08844/2019 | Guangdong | 2019-11-25 | others | D | EPI2211272~EPI2211279 |
| | EPI_ISL_15723918 | A/Environment/Fujian/44488/2019 | Fujian | 2019-11-26 | sewage | A | EPI2211280~EPI2211287 |
| | EPI_ISL_15723919 | A/Environment/Guangxi/44461/2019 | Guangxi | 2019-12-09 | sewage | D | EPI2211288~EPI2211295 |
| | EPI_ISL_15723920 | A/Environment/Guangdong/09270/2019 | Guangdong | 2019-12-10 | feces | A | EPI2211296~EPI2211303 |
| | EPI_ISL_15723921 | A/Environment/Guangdong/09263/2019 | Guangdong | 2019-12-10 | cloacal swab | A | EPI2211304~EPI2211311 |

| Subtype | Isolate Id | Isolate name* | Province | Collection Date | Sample Type | Sample Site† | Accession numbers‡ |
|---|---|---|---|---|---|---|---|
| | EPI_ISL_15723922 | A/Environment/Guangdong/09230/2019 | Guangdong | 2019-12-11 | oropharyngeal swab | A | EPI2211312~EPI2211319 |
| | EPI_ISL_15723923 | A/Environment/Guangxi/03562/2019 | Guangxi | 2019-12-23 | surface swab of cages | D | EPI2211320~EPI2211327 |
| | EPI_ISL_15723925 | A/Environment/Guangdong/14024/2020 | Guangdong | 2020-01-06 | others | D | EPI2211336~EPI2211343 |
| | EPI_ISL_15723926 | A/Environment/Guangxi/08779/2020 | Guangxi | 2020-01-06 | sewage | D | EPI2211344~EPI2211351 |
| | EPI_ISL_15723929 | A/Environment/Hunan/05143/2020 | Hunan | 2020-12-08 | surface swab of cages | D | EPI2211368~EPI2211375 |
| | EPI_ISL_15723930 | A/Environment/Jiangxi/13781/2020 | Jiangxi | 2020-12-08 | surface swab of cages | D | EPI2211376~EPI2211383 |
| | EPI_ISL_15723931 | A/Environment/Jiangxi/13780/2020 | Jiangxi | 2020-12-08 | feces | D | EPI2211384~EPI2211391 |
| | EPI_ISL_15723932 | A/Environment/Chongqing/01452/2021 | Chongqing | 2021-01-20 | surface swab of cages | B | EPI2211392~EPI2211399 |
| | EPI_ISL_15723962 | A/Environment/Chongqing/01458/2021 | Chongqing | 2021-01-20 | surface swab of cages | B | EPI2211400~EPI2211407 |
| | EPI_ISL_15723963 | A/Environment/Hunan/00548/2021 | Hunan | 2021-01-21 | feces | D | EPI2211408~EPI2211415 |
| | EPI_ISL_15723965 | A/Environment/Hunan/05581/2021 | Hunan | 2021-03-18 | feces | D | EPI2211419~EPI2211426 |
| | EPI_ISL_15723966 | A/Environment/Guangxi/12090/2021 | Guangxi | 2021-04-22 | others | A | EPI2211427~EPI2211434 |
| | EPI_ISL_15723967 | A/Environment/Hunan/05683/2021 | Hunan | 2021-04-26 | feces | D | *EPI2211435~EPI2211436* |
| | EPI_ISL_15723968 | A/Environment/Sichuan/03175/2021 | Sichuan | 2021-08-30 | feces | C | EPI2211437~EPI2211444 |
| | EPI_ISL_15723969 | A/Environment/Sichuan/03131/2021 | Sichuan | 2021-08-30 | feces | C | EPI2211445~EPI2211452 |
| | EPI_ISL_15723970 | A/Environment/Guangxi/10369/2021 | Guangxi | 2021-09-07 | sewage | A | EPI2211453~EPI2211460 |
| | EPI_ISL_15723971 | A/Environment/Guangxi/04926/2021 | Guangxi | 2021-10-14 | others | A | EPI2211461~EPI2211468 |
| | EPI_ISL_15723973 | A/Environment/Guangxi/04909/2022 | Guangxi | 2022-01-11 | sewage | A | EPI2211477~EPI2211484 |
| | EPI_ISL_15723976 | A/Environment/Fujian/10650/2022 | Fujian | 2022-01-25 | feces | A | EPI2211501~EPI2211508 |
| | EPI_ISL_15723977 | A/Environment/Fujian/10644/2022 | Fujian | 2022-01-25 | feces | A | EPI2211509~EPI2211516 |
| | EPI_ISL_390760 | ***A/Environment/Guangdong/40751/2017*** | Guangdong | 2017-10-16 | feces | A | EPI1590009~EPI1590016 |
| | EPI_ISL_390761 | ***A/Environment/Chongqing/36536/2017*** | Chongqing | 2017-08-17 | surface swab of cages | A | EPI1590017~EPI1590024 |
| | EPI_ISL_390762 | ***A/Environment/Guangxi/32828/2017*** | Guangxi | 2017-07-04 | sewage | A | EPI1590025~EPI1590032 |
| | EPI_ISL_390763 | ***A/Environment/Hunan/34019/2017*** | Hunan | 2017-04-07 | sewage | A | EPI1590033~EPI1590040 |
| | EPI_ISL_390764 | ***A/Environment/Hubei/34289/2017*** | Hubei | 2017-01-23 | sewage | C | EPI1590041~EPI1590048 |
| | EPI_ISL_390765 | ***A/Environment/Guangxi/14185/2017*** | Guangxi | 2017-01-13 | surface swab of chopping boards | A | EPI1590049~EPI1590056 |
| | EPI_ISL_390766 | ***A/Environment/Guangdong/60350/2016*** | Guangdong | 2016-11-23 | feces | A | EPI1590057~EPI1590064 |
| | EPI_ISL_390767 | ***A/Environment/Anhui/33167/2016*** | Anhui | 2016-04-30 | sewage | A | EPI1590065~EPI1590072 |
| | EPI_ISL_390768 | ***A/Environment/Chongqing/38160/2016*** | Chongqing | 2016-03-18 | feces | A | EPI1590073~EPI1590080 |
| | EPI_ISL_390769 | ***A/Environment/Jiangsu/44006/2016*** | Jiangsu | 2016-03-09 | feces | A | EPI1590081~EPI1590088 |
| | EPI_ISL_390770 | ***A/Environment/Chongqing/22909/2016*** | Chongqing | 2016-01-19 | feces | B | EPI1590089~EPI1590096 |
| | EPI_ISL_390771 | ***A/Environment/Chongqing/22907/2016*** | Chongqing | 2016-01-14 | surface swab of cages | A | EPI1590097~EPI1590104 |
| | EPI_ISL_390772 | ***A/Environment/Hunan/11043/2016*** | Hunan | 2016-01-18 | sewage | A | EPI1590105~EPI1590112 |
| | EPI_ISL_390773 | ***A/Environment/Sichuan/32281/2016*** | Sichuan | 2016-01-08 | sewage | A | EPI1590113~EPI1590120 |
| | EPI_ISL_390774 | ***A/Environment/Inner Mongolia/02114/2015*** | Inner Mongolia | 2015-12-04 | others | C | EPI1590121-EPI1590128 |
| | EPI_ISL_390775 | ***A/Environment/Yunnan/01457/2015*** | Yunnan | 2015-11-27 | surface swab of cages | D | EPI1590129~EPI1590136 |
| | EPI_ISL_390776 | ***A/Environment/Guangdong/15241/2015*** | Guangdong | 2015-09-15 | surface swab of cages | A | EPI1590137~EPI1590144 |
| | EPI_ISL_390777 | ***A/Environment/Hunan/39684/2015*** | Hunan | 2015-04-01 | sewage | A | EPI1590145~EPI1590152 |
| | EPI_ISL_390778 | ***A/Environment/Jiangxi/27771/2015*** | Jiangxi | 2015-03-05 | feces | A | EPI1590153~EPI1590160 |
| | EPI_ISL_390779 | ***A/Environment/Guangdong/21134/2015*** | Guangdong | 2015-01-20 | surface swab of cages | A | EPI1590161~EPI1590168 |
| | EPI_ISL_390780 | ***A/Environment/Hunan/98839/2014*** | Hunan | 2014-10-14 | feces | A | EPI1590169~EPI1590176 |
| | EPI_ISL_390781 | ***A/Environment/Guangxi/79509/2014*** | Guangxi | 2014-09-16 | sewage | A | EPI1590177~EPI1590184 |

| Subtype | Isolate Id | Isolate name* | Province | Collection Date | Sample Type | Sample Site† | Accession numbers‡ |
|---|---|---|---|---|---|---|---|
| | EPI_ISL_390782 | ***A/Environment/Chongqing/79459/2014*** | Chongqing | 2014-09-15 | surface swab of cages | A | EPI1590185~EPI1590192 |
| | EPI_ISL_390783 | ***A/Environment/Guangxi/28753/2014*** | Guangxi | 2014-04-14 | surface swab of chopping boards | A | EPI1590193~EPI1590200 |
| | EPI_ISL_390784 | ***A/Environment/Hunan/26067/2014*** | Hunan | 2014-03-27 | surface swab of cages | A | EPI1590201~EPI1590208 |
| | EPI_ISL_390785 | ***A/Environment/Hunan/18323/2014*** | Hunan | 2014-02-27 | drinking water | A | EPI1590209-EPI1590216 |
| H3N3 | EPI_ISL_15723483 | A/Environment/Guangxi/29409/2018 | Guangxi | 2018-01-23 | surface swab of chopping boards | A | EPI2210728~EPI2210735 |
| | EPI_ISL_15723901 | A/Environment/Guangdong/34076/2019 | Guangdong | 2019-03-13 | feces | A | EPI2211144~EPI2211151 |
| | EPI_ISL_15723927 | A/Environment/Hunan/13561/2020 | Hunan | 2020-06-30 | surface swab of cages | D | EPI2211352~EPI2211359 |
| | EPI_ISL_15723928 | A/Environment/Fujian/14131/2020 | Fujian | 2020-10-28 | feces | D | EPI2211360~EPI2211367 |
| | EPI_ISL_390786 | ***A/Environment/Fujian/02754/2016*** | Fujian | 2016-11-11 | drinking water | A | EPI1590217~EPI1590224 |
| | EPI_ISL_390787 | ***A/Environment/Hunan/03259/2015*** | Hunan | 2015-12-22 | feces | A | EPI1590225~EPI1590232 |
| | EPI_ISL_390788 | ***A/Environment/Fujian/85141/2014*** | Fujian | 2014-10-20 | feces | A | EPI1590233~EPI1590240 |
| H3N6 | EPI_ISL_15720732 | A/Environment/Zhejiang/7/09 | Zhejiang | 2009-01-01 | others | D | EPI2210281~EPI2210288 |
| | EPI_ISL_15723336 | A/Environment/Sichuan/03397/2015 | Sichuan | 2015-12-09 | surface swab of chopping boards | A | EPI2210521~EPI2210528 |
| | EPI_ISL_390789 | ***A/Environment/Sichuan/03404/2015*** | Sichuan | 2015-12-24 | feces | B | EPI1590241~EPI1590248 |
| H3N8 | EPI_ISL_15720734 | A/jilongtumo/Jiangxi/29/09 | Jiangxi | 2009-10-22 | surface swab of cages | A | EPI2210297~EPI2210304 |
| | EPI_ISL_15720735 | A/wushui/Chongqing/2/2010 | Chongqing | 2010-01-27 | sewage | A | EPI2210305~EPI2210312 |
| | EPI_ISL_15720736 | A/jixue/Zhejiang/04/2010 | Zhejiang | 2010-04-01 | chicken blood | A | EPI2210313~EPI2210320 |
| | EPI_ISL_15723505 | A/Environment/Guangxi/32891/2018 | Guangxi | 2018-10-29 | drinking water | A | EPI2210904~EPI2210911 |
| | EPI_ISL_15723924 | A/Environment/Guangxi/03561/2019 | Guangxi | 2019-12-23 | sewage | D | EPI2211328~EPI2211335 |
| | EPI_ISL_15723972 | A/Environment/Guangxi/04905/2022 | Guangxi | 2022-01-11 | others | A | EPI2211469~EPI2211476 |
| | EPI_ISL_15723974 | A/Environment/Fujian/10643/2022 | Fujian | 2022-01-25 | surface swab of cages | A | EPI2211485~EPI2211492 |
| | EPI_ISL_15723975 | A/Environment/Fujian/10648/2022 | Fujian | 2022-01-25 | surface swab of cages | A | EPI2211493~EPI2211500 |
| | EPI_ISL_390790 | ***A/Environment/Hunan/46780/2015*** | Hunan | 2015-11-18 | drinking water | A | EPI1590249~EPI1590256 |
| | EPI_ISL_390791 | ***A/Environment/Hunan/37939/2015*** | Hunan | 2015-04-09 | sewage | A | EPI1590257~EPI1590264 |
| H3N0 | EPI_ISL_15723964 | A/Environment/Guangxi/01993/2021 | Guangxi | 2021-01-27 | sewage | A | *EPI2211416~EPI2211418* |

*Isolate names of strains published in previous study were in italic bold.
†A, live poultry market; B, poultry farm; C, slaughterhouse; D, unknown, referred to sampling sites without specific information (live poultry market, poultry farm, backyard or slaughterhouse).
‡The accession numbers of strains without complete genomes are in italics.

**Appendix Table 2.** H3 subtype avian influenza viruses sampled from the avian-linked environments

| | Sample site, n (%) | | | | |
|---|---|---|---|---|---|
| Subtype | Live poultry markets | Poultry farms/backyards | Slaughterhouses | Unknown* | Total |
| H3N2 | 133(79.6) | 3(1.8) | 6(3.6) | 25(15.0) | 167(88.8) |
| H3N3 | 5(71.4) | 0 | 0 | 2(28.6) | 7(3.7) |
| H3N6 | 1(33.3) | 1(33.3) | 0 | 0 | 3(1.6) |
| H3N8 | 9(0.9) | 0 | 0 | 1(0.1) | 10(5.3) |
| H3Nx | 1(100.0) | 0 | 0 | 0 | 1(0.5) |
| Total | 149(79.3) | 4(2.1) | 6(3.2) | 30(16.0) | 188(100.0) |

*Unknown referred to sampling sites without specific information (live poultry market, poultry farm, backyard, or slaughterhouse).

**Appendix Table 3.** Host distribution in the sublineages China-1 to China-4

| | Sublineage, n(%) | | | | |
|---|---|---|---|---|---|
| Host | China-1 | China-2 | China-3 | China-4 | Total |
| Domestic chicken | 7(4.2) | 4(3.6) | 6(5.5) | 1(4.3) | 18(4.4) |
| Domestic duck | 48(28.9) | 63(56.8) | 80(72.7) | 15(65.2) | 206(50.2) |
| Domestic goose | 0 | 2(1.8) | 0 | 0 | 2(0.5) |
| Other poultry | 0 | 1(0.9) | 0 | 0 | 1(0.2) |
| Wild bird | 1(0.6) | 0 | 4(3.6) | 0 | 5(1.2) |
| Environment | 108(65.1) | 41(36.9) | 20(18.2) | 7(30.4) | 176(42.9) |
| Human | 2(1.2) | 0 | 0 | 0 | 2(0.5) |
| Total | 166 | 111 | 110 | 23 | 410(100.0) |

**Appendix Table 4.** Genotypes of all H3 subtype AIVs with the whole genome and two human H3N8 viruses sampled in China during 2009-2022*.

| Subtype | Isolate name | Genotype | HA | NA | PB2 | PB1 | PA | NP | M | NS |
|---|---|---|---|---|---|---|---|---|---|---|
| H3N2 | A/Environment/Guangdong/03/09 | G1 | China-1 | N2-Eurasian lineage | EA | EA | EA | EA | EA | EA(A) |
| | A/Duck/Guangxi/015D2/2009 | G1 | China-1 | N2-Eurasian lineage | EA | EA | EA | EA | EA | EA(A) |
| | A/Duck/Guangxi/057D6/2010 | G1 | China-1 | N2-Eurasian lineage | EA | EA | EA | EA | EA | EA(A) |
| | A/Chicken/Guangxi/073C2/2010 | G1 | China-1 | N2-Eurasian lineage | EA | EA | EA | EA | EA | EA(A) |
| | A/Duck/Shanghai/27030/2009 | G2 | Worldwide-1 | N2-Eurasian lineage | ZJ-5 | EA | ZJ-5 | ZJ-5 | EA | EA(A) |
| | A/Duck/Guangdong/W12/2011 | G3 | China-1 | N2-Eurasian lineage | EA | EA | EA | ZJ-5 | EA | EA(A) |
| | A/Environment/Hunan/42907/2015 | G3 | China-1 | N2-Eurasian lineage | EA | EA | EA | ZJ-5 | EA | EA(A) |
| | A/Duck/Hebei/B1645-2/2011 | G4 | Worldwide-2 | N2-Eurasian lineage | EA | EA | EA | EA | EA | EA(A) |
| | A/Duck/Hebei/B1647-1/2011 | G4 | Worldwide-2 | N2-Eurasian lineage | EA | EA | EA | EA | EA | EA(A) |
| | A/Duck/Hebei/B1646-2/2011 | G4 | Worldwide-2 | N2-Eurasian lineage | EA | EA | EA | EA | EA | EA(A) |
| | A/Duck/Guangxi/112D4/2012 | G5 | China-1 | waterfowl H6N2 | EA | EA | EA | EA | EA | EA(A) |
| | A/Pigeon/Guangxi/128P9/2012 | G6 | China-2 | N2-Eurasian lineage | EA | EA | EA | EA | ZJ-5 | EA(A) |
| | A/Goose/Guangxi/139G20/2013 | G6 | China-2 | N2-Eurasian lineage | EA | EA | EA | EA | ZJ-5 | EA(A) |
| | A/Environment/Sichuan/32289/2016 | G6 | China-2 | N2-Eurasian lineage | EA | EA | EA | EA | ZJ-5 | EA(A) |
| | A/Environment/Chongqing/22909/2016 | G6 | China-2 | N2-Eurasian lineage | EA | EA | EA | EA | ZJ-5 | EA(A) |
| | A/Environment/Guangdong/14440/2018 | G6 | China-2 | N2-Eurasian lineage | EA | EA | EA | EA | ZJ-5 | EA(A) |
| | A/Environment/Guangdong/14438/2018 | G6 | China-2 | N2-Eurasian lineage | EA | EA | EA | EA | ZJ-5 | EA(A) |
| | A/Environment/Guangdong/14430/2018 | G6 | China-2 | N2-Eurasian lineage | EA | EA | EA | EA | ZJ-5 | EA(A) |
| | A/Environment/Guangdong/14452/2018 | G6 | China-2 | N2-Eurasian lineage | EA | EA | EA | EA | ZJ-5 | EA(A) |
| | A/Environment/Guangxi/32510/2018 | G6 | China-2 | N2-Eurasian lineage | EA | EA | EA | EA | ZJ-5 | EA(A) |
| | A/Environment/Guangxi/34661/2018 | G6 | China-2 | N2-Eurasian lineage | EA | EA | EA | EA | ZJ-5 | EA(A) |
| | A/Environment/Guangxi/39550/2019 | G6 | China-2 | N2-Eurasian lineage | EA | EA | EA | EA | ZJ-5 | EA(A) |
| | A/Chicken/Guangxi/125C8/2012 | G7 | China-2 | N2-Eurasian lineage | EA | EA | ZJ-5 | EA | EA | EA(B) |
| | A/Environment/Guangxi/03059/2012 | G8 | China-3 | N2-Eurasian lineage | ZJ-5 | EA | ZJ-5 | EA | EA | EA(A) |
| | A/Environment/Guangxi/03058/2012 | G8 | China-3 | N2-Eurasian lineage | ZJ-5 | EA | ZJ-5 | EA | EA | EA(A) |
| | A/Environment/Guangxi/03047/2012 | G8 | China-3 | N2-Eurasian lineage | ZJ-5 | EA | ZJ-5 | EA | EA | EA(A) |

| Subtype | Isolate name | Genotype | HA | NA | PB2 | PB1 | PA | NP | M | NS |
|---|---|---|---|---|---|---|---|---|---|---|
| | A/Environment/Anhui/03012/2012 | G9 | China-3 | N2-Eurasian lineage | EA | EA | ZJ-5 | ZJ-5 | ZJ-5 | EA(A) |
| | A/duck/Guangdong/04.16_SZLGWL009/2015_Mixed | G9 | China-3 | N2-Eurasian lineage | EA | EA | ZJ-5 | ZJ-5 | ZJ-5 | EA(A) |
| | A/Environment/Guangxi/41272/2015 | G9 | China-3 | N2-Eurasian lineage | EA | EA | ZJ-5 | ZJ-5 | ZJ-5 | EA(A) |
| | A/Environment/Guangxi/03056/2012 | G10 | China-3 | N2-Eurasian lineage | EA | EA | ZJ-5 | EA | ZJ-5 | EA(A) |
| | A/Duck/Zhejiang/4637/2013 | G11 | China-1 | N2-Eurasian lineage | EA | ZJ-5 | EA | EA | EA | EA(A) |
| | A/Duck/Zhejiang/4613/2013 | G11 | China-1 | N2-Eurasian lineage | EA | ZJ-5 | EA | EA | EA | EA(A) |
| | A/Duck/Zhejiang/4625/2013 | G11 | China-1 | N2-Eurasian lineage | EA | ZJ-5 | EA | EA | EA | EA(A) |
| | A/Duck/Zhejiang/6D7/2013 | G12 | China-1 | N2-Eurasian lineage | EA | EA | ZJ-5 | EA | EA | EA(A) |
| | A/Duck/Zhejiang/D11/2013 | G13 | Worldwide-1 | poultry H9N2 | EA | ZJ-5 | EA | EA | EA | EA(A) |
| | A/Duck/Zhejiang/D13/2013 | G14 | Worldwide-1 | poultry H9N2 | EA | EA | EA | EA | EA | EA(A) |
| | A/environment/Shanghai/LPM1/2013 | G15 | China-2 | N2-Eurasian lineage | EA | EA | EA | EA | EA | EA(A) |
| | A/Environment/Jiangsu/44006/2016 | G15 | China-2 | N2-Eurasian lineage | EA | EA | EA | EA | EA | EA(A) |
| | A/Environment/Chongqing/36536/2017 | G15 | China-2 | N2-Eurasian lineage | EA | EA | EA | EA | EA | EA(A) |
| | A/Environment/Chongqing/36535/2017 | G15 | China-2 | N2-Eurasian lineage | EA | EA | EA | EA | EA | EA(A) |
| | A/Chicken/Guangxi/135C10/2013 | G16 | China-2 | N2-Eurasian lineage | EA | EA | H6 | EA | ZJ-5 | EA(A) |
| | A/Duck/Guangxi/135D20/2013 | G17 | China-2 | N2-Eurasian lineage | EA | H6 | ZJ-5 | EA | ZJ-5 | EA(A) |
| | A/Chicken/Shanghai/LPM2/2013 | G18 | China-3 | N2-Eurasian lineage | EA | EA | EA | EA | ZJ-5 | EA(A) |
| | A/Duck/Shanghai/SH3/2013 | G19 | China-3 | N2-Eurasian lineage | ZJ-5 | EA | ZJ-5 | ZJ-5 | ZJ-5 | ZJ-5 |
| | A/Teal/Shanghai/SH-101/2013 | G20 | China-3 | N2-Eurasian lineage | EA | EA | EA | EA | ZJ-5 | EA(B) |
| | A/Teal/Shanghai/SH-104/2013 | G20 | China-3 | N2-Eurasian lineage | EA | EA | EA | EA | ZJ-5 | EA(B) |
| | A/Teal/Shanghai/SH-89/2013 | G21 | China-3 | N2-Eurasian lineage | EA | EA | EA | ZJ-5 | EA | EA(A) |
| | A/Duck/Shanghai/SH1/2013 | G22 | China-4 | N2-Eurasian lineage | ZJ-5 | ZJ-5 | ZJ-5 | ZJ-5 | ZJ-5 | EA(A) |
| | A/Duck/Shanghai/SH2/2013 | G22 | China-4 | N2-Eurasian lineage | ZJ-5 | ZJ-5 | ZJ-5 | ZJ-5 | ZJ-5 | EA(A) |
| | A/Environment/Guangdong/77214/2014 | G22 | China-4 | N2-Eurasian lineage | ZJ-5 | ZJ-5 | ZJ-5 | ZJ-5 | ZJ-5 | EA(A) |
| | A/Duck/Anhui/D293/2014 | G22 | China-4 | N2-Eurasian lineage | ZJ-5 | ZJ-5 | ZJ-5 | ZJ-5 | ZJ-5 | EA(A) |
| | A/Environment/Guangxi/28750/2014 | G23 | China-1 | N2-Eurasian lineage | EA | EA | EA | EA | ZJ-5 | EA(A) |
| | A/Environment/Guangxi/28753/2014 | G23 | China-1 | N2-Eurasian lineage | EA | EA | EA | EA | ZJ-5 | EA(A) |
| | A/Chicken/Guangxi/165C7/2014 | G23 | China-1 | N2-Eurasian lineage | EA | EA | EA | EA | ZJ-5 | EA(A) |
| | A/Environment/Guangxi/32186/2015 | G23 | China-1 | N2-Eurasian lineage | EA | EA | EA | EA | ZJ-5 | EA(A) |
| | A/Environment/Guangdong/15131/2015 | G23 | China-1 | N2-Eurasian lineage | EA | EA | EA | EA | ZJ-5 | EA(A) |
| | A/Environment/Guangxi/40947/2017 | G23 | China-1 | N2-Eurasian lineage | EA | EA | EA | EA | ZJ-5 | EA(A) |
| | A/Environment/Guangxi/40934/2017 | G23 | China-1 | N2-Eurasian lineage | EA | EA | EA | EA | ZJ-5 | EA(A) |
| | A/Environment/Guangxi/23533/2017 | G23 | China-1 | N2-Eurasian lineage | EA | EA | EA | EA | ZJ-5 | EA(A) |
| | A/Environment/Fujian/36985/2017 | G23 | China-1 | N2-Eurasian lineage | EA | EA | EA | EA | ZJ-5 | EA(A) |
| | A/Environment/Guangxi/05048/2017 | G23 | China-1 | N2-Eurasian lineage | EA | EA | EA | EA | ZJ-5 | EA(A) |
| | A/Environment/Guangxi/32937/2018 | G23 | China-1 | N2-Eurasian lineage | EA | EA | EA | EA | ZJ-5 | EA(A) |
| | A/duck/China/322D22/2018 | G23 | China-1 | N2-Eurasian lineage | EA | EA | EA | EA | ZJ-5 | EA(A) |
| | A/Environment/Guangdong/34268/2018 | G23 | China-1 | N2-Eurasian lineage | EA | EA | EA | EA | ZJ-5 | EA(A) |
| | A/Environment/Guangxi/35164/2018 | G23 | China-1 | N2-Eurasian lineage | EA | EA | EA | EA | ZJ-5 | EA(A) |
| | A/Environment/Guangdong/27641/2018 | G23 | China-1 | N2-Eurasian lineage | EA | EA | EA | EA | ZJ-5 | EA(A) |
| | A/Environment/Guangxi/01886/2018 | G23 | China-1 | N2-Eurasian lineage | EA | EA | EA | EA | ZJ-5 | EA(A) |
| | A/Environment/Guangxi/24886/2018 | G23 | China-1 | N2-Eurasian lineage | EA | EA | EA | EA | ZJ-5 | EA(A) |
| | A/Environment/Guangxi/24803/2018 | G23 | China-1 | N2-Eurasian lineage | EA | EA | EA | EA | ZJ-5 | EA(A) |
| | A/Environment/Guangxi/24775/2018 | G23 | China-1 | N2-Eurasian lineage | EA | EA | EA | EA | ZJ-5 | EA(A) |
| | A/Environment/Guangxi/05712/2018 | G23 | China-1 | N2-Eurasian lineage | EA | EA | EA | EA | ZJ-5 | EA(A) |

| Subtype | Isolate name | Genotype | HA | NA | PB2 | PB1 | PA | NP | M | NS |
|---|---|---|---|---|---|---|---|---|---|---|
| | A/Environment/Guangxi/33582/2018 | G23 | China-1 | N2-Eurasian lineage | EA | EA | EA | EA | ZJ-5 | EA(A) |
| | A/Environment/Guangxi/32515/2018 | G23 | China-1 | N2-Eurasian lineage | EA | EA | EA | EA | ZJ-5 | EA(A) |
| | A/Environment/Guangxi/24805/2018 | G23 | China-1 | N2-Eurasian lineage | EA | EA | EA | EA | ZJ-5 | EA(A) |
| | A/Environment/Guangdong/27250/2018 | G23 | China-1 | N2-Eurasian lineage | EA | EA | EA | EA | ZJ-5 | EA(A) |
| | A/Environment/Guangdong/27249/2018 | G23 | China-1 | N2-Eurasian lineage | EA | EA | EA | EA | ZJ-5 | EA(A) |
| | A/Environment/Guangxi/44461/2019 | G23 | China-1 | N2-Eurasian lineage | EA | EA | EA | EA | ZJ-5 | EA(A) |
| | A/Environment/Guangdong/34240/2019 | G23 | China-1 | N2-Eurasian lineage | EA | EA | EA | EA | ZJ-5 | EA(A) |
| | A/Environment/Guangxi/41094/2019 | G23 | China-1 | N2-Eurasian lineage | EA | EA | EA | EA | ZJ-5 | EA(A) |
| | A/Environment/Guangdong/27245/2019 | G23 | China-1 | N2-Eurasian lineage | EA | EA | EA | EA | ZJ-5 | EA(A) |
| | A/Environment/Fujian/44488/2019 | G23 | China-1 | N2-Eurasian lineage | EA | EA | EA | EA | ZJ-5 | EA(A) |
| | A/Environment/Guangxi/12090/2021 | G23 | China-1 | N2-Eurasian lineage | EA | EA | EA | EA | ZJ-5 | EA(A) |
| | A/Environment/Guangxi/04926/2021 | G23 | China-1 | N2-Eurasian lineage | EA | EA | EA | EA | ZJ-5 | EA(A) |
| | A/Environment/Guangxi/04909/2022 | G23 | China-1 | N2-Eurasian lineage | EA | EA | EA | EA | ZJ-5 | EA(A) |
| | A/Environment/Guangxi/79509/2014 | G24 | China-1 | N2-Eurasian lineage | EA | EA | ZJ-5 | EA | ZJ-5 | EA(A) |
| | A/Environment/Hunan/27451/2014 | G24 | China-1 | N2-Eurasian lineage | EA | EA | ZJ-5 | EA | ZJ-5 | EA(A) |
| | A/Environment/Hunan/11043/2016 | G24 | China-1 | N2-Eurasian lineage | EA | EA | ZJ-5 | EA | ZJ-5 | EA(A) |
| | A/Environment/Guangxi/24894/2018 | G24 | China-1 | N2-Eurasian lineage | EA | EA | ZJ-5 | EA | ZJ-5 | EA(A) |
| | A/Environment/Guangxi/01869/2018 | G24 | China-1 | N2-Eurasian lineage | EA | EA | ZJ-5 | EA | ZJ-5 | EA(A) |
| | A/Environment/Guangdong/08844/2019 | G24 | China-1 | N2-Eurasian lineage | EA | EA | ZJ-5 | EA | ZJ-5 | EA(A) |
| | A/Environment/Guangxi/10369/2021 | G24 | China-1 | N2-Eurasian lineage | EA | EA | ZJ-5 | EA | ZJ-5 | EA(A) |
| | A/Environment/Hunan/26068/2014 | G25 | China-1 | N2-Eurasian lineage | EA | EA | ZJ-5 | ZJ-5 | ZJ-5 | EA(A) |
| | A/Environment/Hunan/37943/2015 | G25 | China-1 | N2-Eurasian lineage | EA | EA | ZJ-5 | ZJ-5 | ZJ-5 | EA(A) |
| | A/duck/Guangdong/04.22_DGCP068-P/2015_Mixed | G25 | China-1 | N2-Eurasian lineage | EA | EA | ZJ-5 | ZJ-5 | ZJ-5 | EA(A) |
| | A/duck/Guangdong/04.22_DGCP075-P/2015_Mixed | G25 | China-1 | N2-Eurasian lineage | EA | EA | ZJ-5 | ZJ-5 | ZJ-5 | EA(A) |
| | A/Environment/Guangdong/60350/2016 | G25 | China-1 | N2-Eurasian lineage | EA | EA | ZJ-5 | ZJ-5 | ZJ-5 | EA(A) |
| | A/Environment/Hunan/25648/2017 | G25 | China-1 | N2-Eurasian lineage | EA | EA | ZJ-5 | ZJ-5 | ZJ-5 | EA(A) |
| | A/Environment/Hunan/05861/2018 | G25 | China-1 | N2-Eurasian lineage | EA | EA | ZJ-5 | ZJ-5 | ZJ-5 | EA(A) |
| | A/Environment/Chongqing/33939/2018 | G25 | China-1 | N2-Eurasian lineage | EA | EA | ZJ-5 | ZJ-5 | ZJ-5 | EA(A) |
| | A/Environment/Guangdong/09230/2019 | G25 | China-1 | N2-Eurasian lineage | EA | EA | ZJ-5 | ZJ-5 | ZJ-5 | EA(A) |
| | A/Environment/Guangdong/09210/2019 | G25 | China-1 | N2-Eurasian lineage | EA | EA | ZJ-5 | ZJ-5 | ZJ-5 | EA(A) |
| | A/Environment/Hubei/43399/2019 | G25 | China-1 | N2-Eurasian lineage | EA | EA | ZJ-5 | ZJ-5 | ZJ-5 | EA(A) |
| | A/Environment/Hunan/26067/2014 | G26 | China-1 | N2-Eurasian lineage | EA | EA | EA | ZJ-5 | ZJ-5 | EA(A) |
| | A/Environment/Hunan/98839/2014 | G26 | China-1 | N2-Eurasian lineage | EA | EA | EA | ZJ-5 | ZJ-5 | EA(A) |
| | A/Environment/Guangxi/32828/2017 | G26 | China-1 | N2-Eurasian lineage | EA | EA | EA | ZJ-5 | ZJ-5 | EA(A) |
| | A/Environment/Guangxi/32906/2018 | G26 | China-1 | N2-Eurasian lineage | EA | EA | EA | ZJ-5 | ZJ-5 | EA(A) |
| | A/Environment/Guangdong/30702/2018 | G26 | China-1 | N2-Eurasian lineage | EA | EA | EA | ZJ-5 | ZJ-5 | EA(A) |
| | A/Environment/Guangxi/32383/2019 | G26 | China-1 | N2-Eurasian lineage | EA | EA | EA | ZJ-5 | ZJ-5 | EA(A) |
| | A/Environment/Guangxi/39142/2019 | G26 | China-1 | N2-Eurasian lineage | EA | EA | EA | ZJ-5 | ZJ-5 | EA(A) |
| | A/Environment/Guangdong/09270/2019 | G26 | China-1 | N2-Eurasian lineage | EA | EA | EA | ZJ-5 | ZJ-5 | EA(A) |
| | A/Environment/Guangdong/09263/2019 | G26 | China-1 | N2-Eurasian lineage | EA | EA | EA | ZJ-5 | ZJ-5 | EA(A) |
| | A/Environment/Guangdong/14024/2020 | G26 | China-1 | N2-Eurasian lineage | EA | EA | EA | ZJ-5 | ZJ-5 | EA(A) |
| | A/Environment/Hunan/02458/2014 | G27 | Worldwide-1 | N2-Eurasian lineage | EA | EA | ZJ-5 | ZJ-5 | ZJ-5 | EA(A) |
| | A/duck/Guangxi/04.10_JX050/2015_Mixed | G27 | Worldwide-1 | N2-Eurasian lineage | EA | EA | ZJ-5 | ZJ-5 | ZJ-5 | EA(A) |
| | A/Environment/Guangdong/40751/2017 | G27 | Worldwide-1 | N2-Eurasian lineage | EA | EA | ZJ-5 | ZJ-5 | ZJ-5 | EA(A) |
| | A/chicken/Jiangsu/12.30_WZNHQ013-P/2014_Mixed | G28 | China-3 | N2-Eurasian lineage | H9N2 | ZJ-5 | ZJ-5 | H9N2 | ZJ-5 | H9N2 |

| Subtype | Isolate name | Genotype | HA | NA | PB2 | PB1 | PA | NP | M | NS |
|---|---|---|---|---|---|---|---|---|---|---|
| | A/duck/Jiangsu/12.18_NJLH1266-P/2014_Mixed | G29 | China-3 | N2-Eurasian lineage | EA | ZJ-5 | ZJ-5 | EA | EA | ZJ-5 |
| | A/water/Hunan/140/2014 | G30 | China-3 | N2-Eurasian lineage | ZJ-5 | EA | ZJ-5 | ZJ-5 | ZJ-5 | EA(A) |
| | A/Environment/Hunan/31561/2016 | G30 | China-3 | N2-Eurasian lineage | ZJ-5 | EA | ZJ-5 | ZJ-5 | ZJ-5 | EA(A) |
| | A/Environment/Guangdong/35458/2017 | G30 | China-3 | N2-Eurasian lineage | ZJ-5 | EA | ZJ-5 | ZJ-5 | ZJ-5 | EA(A) |
| | A/Environment/Chongqing/79459/2014 | G31 | China-3 | N2-Eurasian lineage | EA | EA | EA | ZJ-5 | ZJ-5 | EA(A) |
| | A/Environment/Yunnan/01456/2015 | G31 | China-3 | N2-Eurasian lineage | EA | EA | EA | ZJ-5 | ZJ-5 | EA(A) |
| | A/Environment/Yunnan/01457/2015 | G31 | China-3 | N2-Eurasian lineage | EA | EA | EA | ZJ-5 | ZJ-5 | EA(A) |
| | A/Environment/Guangdong/21134/2015 | G31 | China-3 | N2-Eurasian lineage | EA | EA | EA | ZJ-5 | ZJ-5 | EA(A) |
| | A/Environment/Hunan/18323/2014 | G32 | China-4 | N2-Eurasian lineage | ZJ-5 | ZJ-5 | ZJ-5 | ZJ-5 | ZJ-5 | ZJ-5 |
| | A/environment/Jiangxi/05.07_NCJD0096C/2015_Mixed | G33 | China-1 | N2-Eurasian lineage | EA | ZJ-5 | EA | ZJ-5 | EA | EA(A) |
| | A/duck/Guangdong/04.22_DGCP070-O/2015_Mixed | G34 | China-1 | N2-Eurasian lineage | H6 | EA | ZJ-5 | ZJ-5 | EA | EA(A) |
| | A/duck/Guangdong/04.22_DGCP064-P/2015_Mixed | G35 | China-1 | N2-Eurasian lineage | EA | EA | ZJ-5 | ZJ-5 | EA | EA(A) |
| | A/duck/Guangdong/04.22_DGCP078-P/2015_Mixed | G35 | China-1 | N2-Eurasian lineage | EA | EA | ZJ-5 | ZJ-5 | EA | EA(A) |
| | A/Duck/Guangdong/04.22_DGCP074-P/2015 | G35 | China-1 | N2-Eurasian lineage | EA | EA | ZJ-5 | ZJ-5 | EA | EA(A) |
| | A/Environment/Hunan/10170/2016 | G35 | China-1 | N2-Eurasian lineage | EA | EA | ZJ-5 | ZJ-5 | EA | EA(A) |
| | A/Environment/Guangxi/14185/2017 | G35 | China-1 | N2-Eurasian lineage | EA | EA | ZJ-5 | ZJ-5 | EA | EA(A) |
| | A/environment/Jiangxi/02.05_YGYXG010/2015 | G36 | Europe-Asia | N2-Eurasian lineage | EA | EA | ZJ-5 | EA | EA | EA(A) |
| | A/EV/SD/226238/2015 | G37 | Worldwide-1 | N2-Eurasian lineage | EA | EA | EA | EA | EA | EA(A) |
| | A/EV/SD/226214/2015 | G37 | Worldwide-1 | N2-Eurasian lineage | EA | EA | EA | EA | EA | EA(A) |
| | A/Environment/Sichuan/48404/2016 | G37 | Worldwide-1 | N2-Eurasian lineage | EA | EA | EA | EA | EA | EA(A) |
| | A/Environment/Chongqing/45115/2015 | G38 | China-2 | N2-Eurasian lineage | ZJ-5 | ZJ-5 | ZJ-5 | ZJ-5 | ZJ-5 | ZJ-5 |
| | A/Environment/Zhejiang/31298/2018 | G38 | China-2 | N2-Eurasian lineage | ZJ-5 | ZJ-5 | ZJ-5 | ZJ-5 | ZJ-5 | ZJ-5 |
| | A/Environment/Hunan/39684/2015 | G39 | China-2 | N2-Eurasian lineage | EA | EA | ZJ-5 | ZJ-5 | ZJ-5 | EA(A) |
| | A/Environment/Hunan/34019/2017 | G39 | China-2 | N2-Eurasian lineage | EA | EA | ZJ-5 | ZJ-5 | ZJ-5 | EA(A) |
| | A/Environment/Guangxi/01885/2018 | G39 | China-2 | N2-Eurasian lineage | EA | EA | ZJ-5 | ZJ-5 | ZJ-5 | EA(A) |
| | A/Environment/Guangxi/01878/2018 | G39 | China-2 | N2-Eurasian lineage | EA | EA | ZJ-5 | ZJ-5 | ZJ-5 | EA(A) |
| | A/Environment/Guangxi/03562/2019 | G39 | China-2 | N2-Eurasian lineage | EA | EA | ZJ-5 | ZJ-5 | ZJ-5 | EA(A) |
| | A/Environment/Gansu/01469/2019 | G39 | China-2 | N2-Eurasian lineage | EA | EA | ZJ-5 | ZJ-5 | ZJ-5 | EA(A) |
| | A/Environment/Guangxi/08779/2020 | G39 | China-2 | N2-Eurasian lineage | EA | EA | ZJ-5 | ZJ-5 | ZJ-5 | EA(A) |
| | A/Environment/Hunan/34583/2015 | G40 | China-2 | N2-Eurasian lineage | ZJ-5 | EA | EA | EA | ZJ-5 | EA(A) |
| | A/duck/Hunan/04.14_YYGK0451-Z-O/2015_Mixed | G41 | China-2 | N2-Eurasian lineage | H6 | EA | ZJ-5 | ZJ-5 | ZJ-5 | EA(A) |
| | A/environment/Jiangxi/01.08_YGCB009/2015_Mixed | G42 | China-3 | N2-Eurasian lineage | EA | EA | ZJ-5 | EA | EA | EA(A) |
| | A/Chicken/Jiangxi/01.08_YGCB018-P/2015 | G42 | China-3 | N2-Eurasian lineage | EA | EA | ZJ-5 | EA | EA | EA(A) |
| | A/Chicken/Jiangxi/02.05_YGYXG001-P/2015 | G42 | China-3 | N2-Eurasian lineage | EA | EA | ZJ-5 | EA | EA | EA(A) |
| | A/duck/Jiangxi/01.08_YGCB023-P/2015_Mixed | G42 | China-3 | N2-Eurasian lineage | EA | EA | ZJ-5 | EA | EA | EA(A) |
| | A/Environment/Jiangxi/27771/2015 | G42 | China-3 | N2-Eurasian lineage | EA | EA | ZJ-5 | EA | EA | EA(A) |
| | A/duck/Jiangxi/01.14_NCJD033-P/2015_Mixed | G43 | China-3 | N2-Eurasian lineage | EA | ZJ-5 | ZJ-5 | ZJ-5 | ZJ-5 | EA(A) |
| | A/Duck/Jiangxi/01.14_NCJD038-P/2015 | G43 | China-3 | N2-Eurasian lineage | EA | ZJ-5 | ZJ-5 | ZJ-5 | ZJ-5 | EA(A) |
| | A/Duck/Jiangxi/01.14_NCJD035-P/2015 | G43 | China-3 | N2-Eurasian lineage | EA | ZJ-5 | ZJ-5 | ZJ-5 | ZJ-5 | EA(A) |
| | A/duck/Guangdong/04.23_DGQTSJ126-P/2015_Mixed | G44 | China-3 | N2-Eurasian lineage | ZJ-5 | ZJ-5 | ZJ-5 | ZJ-5 | ZJ-5 | EA(A) |
| | A/duck/Guangdong/04.23_DGQTSJ123-P/2015_Mixed | G44 | China-3 | N2-Eurasian lineage | ZJ-5 | ZJ-5 | ZJ-5 | ZJ-5 | ZJ-5 | EA(A) |
| | A/duck/Guangdong/04.23_DGQTSJ122-P/2015_Mixed | G44 | China-3 | N2-Eurasian lineage | ZJ-5 | ZJ-5 | ZJ-5 | ZJ-5 | ZJ-5 | EA(A) |
| | A/environment/Guangdong/04.23_DGQTSJ037/2015_Mixed | G44 | China-3 | N2-Eurasian lineage | ZJ-5 | ZJ-5 | ZJ-5 | ZJ-5 | ZJ-5 | EA(A) |
| | A/duck/Guangdong/04.23_DGQTSJ126-O/2015_Mixed | G45 | China-3 | N2-Eurasian lineage | H6 | ZJ-5 | ZJ-5 | ZJ-5 | ZJ-5 | EA(A) |

| Subtype | Isolate name | Genotype | HA | NA | PB2 | PB1 | PA | NP | M | NS |
|---|---|---|---|---|---|---|---|---|---|---|
| | A/Environment/Guangdong/15241/2015 | G46 | China-4 | N2-Eurasian lineage | EA | EA | EA | ZJ-5 | ZJ-5 | EA(A) |
| | A/Environment/Guangdong/15077/2015 | G47 | China-4 | N2-Eurasian lineage | ZJ-5 | EA | ZJ-5 | ZJ-5 | ZJ-5 | EA(A) |
| | A/Bean_Goose/Hubei/chenhu_XVI35-1/2016 | G48 | Europe-Asia | North America-1 | EA | EA | EA | EA | EA | EA(A) |
| | A/Chicken/Ganzhou/GZ157/2016 | G49 | Worldwide-1 | N2-Eurasian lineage | ZJ-5 | EA | ZJ-5 | ZJ-5 | ZJ-5 | EA(A) |
| | A/Chicken/Ganzhou/GZ43/2016 | G49 | Worldwide-1 | N2-Eurasian lineage | ZJ-5 | EA | ZJ-5 | ZJ-5 | ZJ-5 | EA(A) |
| | A/Environment/Anhui/33167/2016 | G49 | Worldwide-1 | N2-Eurasian lineage | ZJ-5 | EA | ZJ-5 | ZJ-5 | ZJ-5 | EA(A) |
| | A/Environment/Sichuan/32281/2016 | G50 | Worldwide-1 | N2-Eurasian lineage | EA | EA | EA | EA | NA | EA(A) |
| | A/Environment/Chongqing/38160/2016 | G51 | Worldwide-1 | N2-Eurasian lineage | EA | EA | EA | EA | EA | EA(B) |
| | A/Environment/Chongqing/22907/2016 | G51 | Worldwide-1 | N2-Eurasian lineage | EA | EA | EA | EA | EA | EA(B) |
| | A/Duck/Jiangsu/YZ916/2016 | G52 | China-2 | N2-Eurasian lineage | ZJ-5 | ZJ-5 | EA | ZJ-5 | ZJ-5 | EA(A) |
| | A/Environment/Jiangsu/06689/2016 | G53 | China-2 | N2-Eurasian lineage | EA | EA | EA | ZJ-5 | ZJ-5 | EA(A) |
| | A/Environment/Chongqing/41153/2019 | G53 | China-2 | N2-Eurasian lineage | EA | EA | EA | ZJ-5 | ZJ-5 | EA(A) |
| | A/Environment/Guangxi/39500/2019 | G53 | China-2 | N2-Eurasian lineage | EA | EA | EA | ZJ-5 | ZJ-5 | EA(A) |
| | A/Environment/Guangxi/32152/2017 | G54 | China-1 | N2-Eurasian lineage | ZJ-5 | EA | EA | EA | ZJ-5 | EA(A) |
| | A/Environment/Guangxi/28076/2018 | G54 | China-1 | N2-Eurasian lineage | ZJ-5 | EA | EA | EA | ZJ-5 | EA(A) |
| | A/Environment/Fujian/39244/2019 | G54 | China-1 | N2-Eurasian lineage | ZJ-5 | EA | EA | EA | ZJ-5 | EA(A) |
| | A/Environment/Guangdong/27246/2019 | G54 | China-1 | N2-Eurasian lineage | ZJ-5 | EA | EA | EA | ZJ-5 | EA(A) |
| | A/Environment/Fujian/10644/2022 | G54 | China-1 | N2-Eurasian lineage | ZJ-5 | EA | EA | EA | ZJ-5 | EA(A) |
| | A/Environment/Fujian/10650/2022 | G54 | China-1 | N2-Eurasian lineage | ZJ-5 | EA | EA | EA | ZJ-5 | EA(A) |
| | A/Environment/Fujian/04958/2017 | G55 | China-1 | N2-Eurasian lineage | EA | ZJ-5 | EA | EA | ZJ-5 | EA(A) |
| | A/Environment/Hunan/25643/2017 | G56 | China-1 | N2-Eurasian lineage | ZJ-5 | EA | EA | ZJ-5 | ZJ-5 | EA(A) |
| | A/Environment/Hunan/34012/2017 | G56 | China-1 | N2-Eurasian lineage | ZJ-5 | EA | EA | ZJ-5 | ZJ-5 | EA(A) |
| | A/Environment/Hunan/04235/2018 | G56 | China-1 | N2-Eurasian lineage | ZJ-5 | EA | EA | ZJ-5 | ZJ-5 | EA(A) |
| | A/Environment/Hunan/28854/2018 | G56 | China-1 | N2-Eurasian lineage | ZJ-5 | EA | EA | ZJ-5 | ZJ-5 | EA(A) |
| | A/Environment/Hunan/40453/2019 | G56 | China-1 | N2-Eurasian lineage | ZJ-5 | EA | EA | ZJ-5 | ZJ-5 | EA(A) |
| | A/Environment/Hunan/40442/2019 | G56 | China-1 | N2-Eurasian lineage | ZJ-5 | EA | EA | ZJ-5 | ZJ-5 | EA(A) |
| | A/Environment/Hunan/05143/2020 | G56 | China-1 | N2-Eurasian lineage | ZJ-5 | EA | EA | ZJ-5 | ZJ-5 | EA(A) |
| | A/Environment/Hunan/00548/2021 | G56 | China-1 | N2-Eurasian lineage | ZJ-5 | EA | EA | ZJ-5 | ZJ-5 | EA(A) |
| | A/Environment/Hunan/05581/2021 | G56 | China-1 | N2-Eurasian lineage | ZJ-5 | EA | EA | ZJ-5 | ZJ-5 | EA(A) |
| | A/Environment/Guangxi/23558/2017 | G57 | Worldwide-1 | N2-Eurasian lineage | ZJ-5 | EA | EA | ZJ-5 | ZJ-5 | EA(A) |
| | A/Environment/Guangxi/32060/2017 | G58 | Worldwide-1 | N2-Eurasian lineage | EA | EA | EA | ZJ-5 | EA | EA(B) |
| | A/Environment/Hubei/33702/2017 | G59 | China-2 | N2-Eurasian lineage | EA | EA | ZJ-5 | EA | ZJ-5 | EA(A) |
| | A/Environment/Hubei/34289/2017 | G60 | China-3 | N2-Eurasian lineage | EA | EA | ZJ-5 | ZJ-5 | EA | EA(A) |
| | A/Environment/Guangdong/32693/2018 | G61 | China-1 | N2-Eurasian lineage | EA | EA | EA | EA | ZJ-5 | EA(B) |
| | A/Environment/Guangdong/27727/2018 | G62 | China-1 | N2-Eurasian lineage | ZJ-5 | ZJ-5 | ZJ-5 | ZJ-5 | ZJ-5 | EA(A) |
| | A/Environment/Guangxi/33095/2018 | G62 | China-1 | N2-Eurasian lineage | ZJ-5 | ZJ-5 | ZJ-5 | ZJ-5 | ZJ-5 | EA(A) |
| | A/Environment/Chongqing/01458/2021 | G62 | China-1 | N2-Eurasian lineage | ZJ-5 | ZJ-5 | ZJ-5 | ZJ-5 | ZJ-5 | EA(A) |
| | A/Environment/Guangxi/24790/2018 | G63 | China-1 | N2-Eurasian lineage | ZJ-5 | EA | ZJ-5 | ZJ-5 | ZJ-5 | EA(A) |
| | A/Environment/Guangxi/45299/2019 | G63 | China-1 | N2-Eurasian lineage | ZJ-5 | EA | ZJ-5 | ZJ-5 | ZJ-5 | EA(A) |
| | A/Environment/Hubei/43318/2019 | G63 | China-1 | N2-Eurasian lineage | ZJ-5 | EA | ZJ-5 | ZJ-5 | ZJ-5 | EA(A) |
| | A/Environment/Chongqing/01452/2021 | G63 | China-1 | N2-Eurasian lineage | ZJ-5 | EA | ZJ-5 | ZJ-5 | ZJ-5 | EA(A) |
| | A/Environment/Chongqing/33926/2018 | G64 | Worldwide-1 | N2-Eurasian lineage | EA | EA | ZJ-5 | EA | ZJ-5 | EA(A) |
| | A/Environment/Guangdong/27940/2018 | G65 | Worldwide-1 | N2-Eurasian lineage | EA | EA | EA | EA | ZJ-5 | EA(A) |
| | A/Environment/Guangdong/30984/2018 | G66 | China-2 | N2-Eurasian lineage | EA | ZJ-5 | ZJ-5 | EA | ZJ-5 | EA(A) |
| | A/Environment/Guangxi/33056/2018 | G67 | China-2 | N2-Eurasian lineage | ZJ-5 | EA | ZJ-5 | EA | EA | EA(A) |

| Subtype | Isolate name | Genotype | HA | NA | PB2 | PB1 | PA | NP | M | NS |
|---|---|---|---|---|---|---|---|---|---|---|
| | A/Environment/Zhejiang/02021/2018 | G68 | China-2 | N2-Eurasian lineage | ZJ-5 | ZJ-5 | ZJ-5 | ZJ-5 | ZJ-5 | EA(A) |
| | A/Environment/Guangdong/27729/2018 | G69 | China-4 | N2-Eurasian lineage | ZJ-5 | EA | ZJ-5 | EA | ZJ-5 | EA(A) |
| | A/Environment/Guangxi/45278/2019 | G70 | Worldwide-1 | N2-Eurasian lineage | EA | EA | EA | ZJ-5 | ZJ-5 | EA(A) |
| | A/Environment/Guangdong/34622/2019 | G71 | China-2 | N2-Eurasian lineage | ZJ-5 | EA | ZJ-5 | ZJ-5 | ZJ-5 | EA(A) |
| | A/duck/China/Influenza_A_virus/2019 | G72 | China-2 | N2-Eurasian lineage | EA | EA | ZJ-5 | ZJ-5 | EA | EA(A) |
| | A/Environment/Sichuan/03131/2021 | G73 | China-2 | N2-Eurasian lineage | ZJ-5 | EA | EA | ZJ-5 | ZJ-5 | EA(A) |
| | A/Environment/Sichuan/03175/2021 | G73 | China-2 | N2-Eurasian lineage | ZJ-5 | EA | EA | ZJ-5 | ZJ-5 | EA(A) |
| H3N3 | A/Duck/Zhejiang/5/2011 | G1 | China-4 | N3-Eurasian lineage | ZJ-5 | ZJ-5 | ZJ-5 | ZJ-5 | ZJ-5 | ZJ-5 |
| | A/Duck/Zhejiang/D16/2013 | G2 | Worldwide-1 | N3-Eurasian lineage | EA | EA | EA | EA | EA | EA(A) |
| | A/Duck/Zhejiang/D17/2013 | G2 | Worldwide-1 | N3-Eurasian lineage | EA | EA | EA | EA | EA | EA(A) |
| | A/Duck/Zhejiang/D18/2013 | G2 | Worldwide-1 | N3-Eurasian lineage | EA | EA | EA | EA | EA | EA(A) |
| | A/Curlew/Zhuanghe/ZH-47/2013 | G3 | Asia | N3-Eurasian lineage | EA | EA | EA | EA | EA | EA(A) |
| | A/environment/Jiangxi/05.07_NCJD0042C/2015_Mixed | G4 | China-1 | N3-Eurasian lineage | EA | EA | ZJ-5 | ZJ-5 | ZJ-5 | EA(A) |
| | A/Environment/Hunan/03259/2015 | G5 | China-1 | N3-Eurasian lineage | EA | EA | EA | EA | EA | EA(A) |
| | A/duck/Jiangxi/01.14_NCJD059-P/2015_Mixed | G6 | China-3 | N3-Eurasian lineage | ZJ-5 | ZJ-5 | ZJ-5 | EA | ZJ-5 | EA(A) |
| | A/Environment/Fujian/02754/2016 | G7 | China-1 | N3-Eurasian lineage | ZJ-5 | ZJ-5 | ZJ-5 | ZJ-5 | ZJ-5 | EA(A) |
| | A/Environment/Hunan/13561/2020 | G7 | China-1 | N3-Eurasian lineage | ZJ-5 | ZJ-5 | ZJ-5 | ZJ-5 | ZJ-5 | EA(A) |
| | A/Duck/Fujian/SD063/2017 | G8 | China-1 | N3-Eurasian lineage | ZJ-5 | ZJ-5 | ZJ-5 | EA | ZJ-5 | EA(B) |
| | A/Environment/Guangxi/29409/2018 | G9 | China-1 | N3-Eurasian lineage | EA | EA | EA | EA | ZJ-5 | EA(A) |
| | A/Environment/Guangdong/34076/2019 | G10 | China-2 | N3-Eurasian lineage | EA | EA | EA | ZJ-5 | ZJ-5 | EA(A) |
| | A/Environment/Fujian/14131/2020 | G11 | China-1 | N3-Eurasian lineage | EA | ZJ-5 | ZJ-5 | EA | ZJ-5 | EA(B) |
| H3N6 | A/Pigeon/Guangxi/020P/2009 | G1 | Eurasian lineage | N6-Eurasian lineage | EA | EA | EA | EA | EA | EA(A) |
| | A/Environment/Zhejiang/7/09 | G2 | China-4 | N6-Eurasian lineage | EA | EA | ZJ-5 | ZJ-5 | EA | EA(A) |
| | A/Duck/Jiangsu/4/2010 | G3 | Asia | N6-Eurasian lineage | EA | EA | EA | EA | H6 | EA(A) |
| | A/Curlew/Zhuanghe/ZH-64/2013 | G4 | Asia | N6-Eurasian lineage | EA | EA | EA | EA | EA | EA(A) |
| | A/Curlew/Zhuanghe/ZH-65/2013 | G4 | Asia | N6-Eurasian lineage | EA | EA | EA | EA | EA | EA(A) |
| | A/Duck/Zhejiang/D1-1/2013 | G5 | China-4 | N6-Eurasian lineage | ZJ-5 | ZJ-5 | ZJ-5 | ZJ-5 | ZJ-5 | ZJ-5 |
| | A/Duck/Zhejiang/D1-2/2013 | G5 | China-4 | N6-Eurasian lineage | ZJ-5 | ZJ-5 | ZJ-5 | ZJ-5 | ZJ-5 | ZJ-5 |
| | A/Duck/Zhejiang/D1-3/2013 | G5 | China-4 | N6-Eurasian lineage | ZJ-5 | ZJ-5 | ZJ-5 | ZJ-5 | ZJ-5 | ZJ-5 |
| | A/Duck/Guangxi/175D12/2014 | G6 | China-1 | H5N6 sublineage | H5N6 | EA | ZJ-5 | H5N6 | ZJ-5 | EA(A) |
| | A/Duck/Hunan/146/2014 | G7 | China-2 | H5N6 sublineage | H5N6 | H5N6 | H5N6 | H5N6 | H5N6 | H5N6 |
| | A/duck/Guangdong/04.16_SZLGWL012/2015_Mixed | G8 | China-1 | H5N6 sublineage | H6 | EA | H9N2 | H9N2 | ZJ-5 | H9N2 |
| | A/Duck/Hunan/161/2015 | G9 | China-1 | H5N6 sublineage | ZJ-5 | EA | ZJ-5 | ZJ-5 | ZJ-5 | EA(A) |
| | A/Duck/Hubei/ZYSYF9/2015 | G10 | China-1 | waterfowl H6N6 | ZJ-5 | ZJ-5 | EA | ZJ-5 | ZJ-5 | EA(A) |
| | A/Duck/Hubei/ZYSYF2/2015 | G10 | China-1 | waterfowl H6N6 | ZJ-5 | ZJ-5 | EA | ZJ-5 | ZJ-5 | EA(A) |
| | A/Duck/Hubei/ZYSYF21/2015 | G10 | China-1 | waterfowl H6N6 | ZJ-5 | ZJ-5 | EA | ZJ-5 | ZJ-5 | EA(A) |
| | A/Duck/Hubei/ZYSYF4/2015 | G11 | China-1 | N6-Eurasian lineage | EA | EA | ZJ-5 | ZJ-5 | ZJ-5 | EA(A) |
| | A/Duck/Hubei/ZYSYF18/2015 | G11 | China-1 | N6-Eurasian lineage | EA | EA | ZJ-5 | ZJ-5 | ZJ-5 | EA(A) |
| | A/Duck/Hubei/ZYSYF12/2015 | G11 | China-1 | N6-Eurasian lineage | EA | EA | ZJ-5 | ZJ-5 | ZJ-5 | EA(A) |
| | A/Duck/Hunan/7/2015 | G12 | China-1 | H5N6 sublineage | EA | EA | EA | ZJ-5 | ZJ-5 | EA(A) |
| | A/environment/Jiangxi/01.14_NCJD024/2015_Mixed | G13 | China-1 | waterfowl H6N6 | H6 | EA | H6 | EA | EA | EA(A) |
| | A/environment/Jiangxi/01.08_YGCB016/2015_Mixed | G14 | Europe-Asia | H5N6 sublineage | H6 | EA | ZJ-5 | EA | EA | H5N6 |
| | A/duck/Jiangxi/01.14_NCJD060-P/2015_Mixed | G15 | Europe-Asia | H5N6 sublineage | H5N6 | EA | ZJ-5 | EA | EA | EA(A) |
| | A/Environment/Sichuan/03404/2015 | G16 | Worldwide-1 | N6-Eurasian lineage | EA | EA | EA | EA | EA | EA(B) |

| Subtype | Isolate name | Genotype | HA | NA | PB2 | PB1 | PA | NP | M | NS |
|---|---|---|---|---|---|---|---|---|---|---|
| | A/Environment/Sichuan/03397/2015 | G16 | Worldwide-1 | N6-Eurasian lineage | EA | EA | EA | EA | EA | EA(B) |
| | A/duck/Jiangxi/01.14_NCJD057-P/2015_Mixed | G17 | China-3 | H5N6 sublineage | ZJ-5 | ZJ-5 | ZJ-5 | ZJ-5 | ZJ-5 | EA(A) |
| H3N8 | A/Duck/Shanghai/120-1/2009 | G1 | Asia | N8-Eurasian lineage | EA | EA | EA | EA | ZJ-5 | EA(A) |
| | A/jilongtumo/Jiangxi/29/09 | G2 | China-2 | N8-North American lineage | EA | EA | EA | EA | EA | EA(A) |
| | A/Duck/Wuxi/7275/2016 | G2 | China-2 | N8-North American lineage | EA | EA | EA | EA | EA | EA(A) |
| | A/Goose/Guangxi/020G/2009 | G3 | Eurasian lineage | N8-North American lineage | EA | EA | EA | EA | EA | EA(A) |
| | A/Duck/Nanjing/A1591-1/2010 | G4 | China-3 | N8-North American lineage | EA | EA | ZJ-5 | ZJ-5 | ZJ-5 | EA(A) |
| | A/Chicken/Nanjing/B854-2/2011 | G4 | China-3 | N8-North American lineage | EA | EA | ZJ-5 | ZJ-5 | ZJ-5 | EA(A) |
| | A/Duck/Hunan/199/2014 | G4 | China-3 | N8-North American lineage | EA | EA | ZJ-5 | ZJ-5 | ZJ-5 | EA(A) |
| | A/jixue/Zhejiang/04/2010 | G5 | China-4 | N8-North American lineage | ZJ-5 | EA | ZJ-5 | ZJ-5 | EA | EA(A) |
| | A/environment/Hunan/S4350/2011 | G6 | China-1 | N8-North American lineage | EA | EA | EA | EA | EA | EA(A) |
| | A/Environment/Hunan/37939/2015 | G6 | China-1 | N8-North American lineage | EA | EA | EA | EA | EA | EA(A) |
| | A/Duck/Hunan/S1256/2012 | G7 | Worldwide-1 | N8-Eurasian lineage | EA | EA | EA | EA | EA | EA(B) |
| | A/Duck/Hunan/S1824/2012 | G8 | China-4 | N8-North American lineage | EA | EA | EA | EA | EA | EA(A) |
| | A/Teal/Shanghai/SH-90/2013 | G9 | Worldwide-1 | N8-North American lineage | EA | EA | EA | EA | EA | NA |
| | A/Duck/Zhejiang/4812/2013 | G10 | China-2 | N8-North American lineage | EA | ZJ-5 | ZJ-5 | EA | EA | ZJ-5 |
| | A/Duck/Zhejiang/D1-6/2013 | G11 | China-4 | N8-Eurasian lineage | ZJ-5 | ZJ-5 | ZJ-5 | ZJ-5 | ZJ-5 | ZJ-5 |
| | A/Mallard/Jiangxi/G98/2014 | G12 | Europe-Asia | N8-Eurasian lineage | EA | H5N6 | EA | EA | H5N6 | EA(A) |
| | A/Duck/Guangdong/04.22_DGCP083-P/2015 | G13 | China-1 | N8-North American lineage | EA | EA | EA | ZJ-5 | ZJ-5 | EA(A) |
| | A/Duck/Guangdong/03.26_DGCP072-P/2015 | G14 | China-1 | N8-North American lineage | EA | EA | ZJ-5 | ZJ-5 | EA | EA(A) |
| | A/duck/Hunan/04.14_YYGK431-P/2015_Mixed | G15 | Europe-Asia | N8-North American lineage | EA | EA | EA | EA | EA | EA(A) |
| | A/Environment/Hunan/46780/2015 | G16 | Worldwide-1 | N8-North American lineage | EA | EA | ZJ-5 | ZJ-5 | ZJ-5 | EA(A) |
| | A/Chicken/Wuxi/4859/2015 | G17 | China-2 | N8-North American lineage | EA | EA | ZJ-5 | H5N1 | EA | EA(A) |
| | A/Mallard/Xuyi/14/2015 | G18 | China-3 | N8-North American lineage | EA | EA | ZJ-5 | EA | ZJ-5 | EA(A) |
| | A/duck/Jiangxi/01.14_NCJD031-P/2015_Mixed | G19 | China-3 | N8-North American lineage | EA | EA | ZJ-5 | EA | EA | EA(B) |
| | A/duck/Jiangxi/01.14_NCJD058-P/2015_Mixed | G20 | China-3 | N8-North American lineage | ZJ-5 | ZJ-5 | ZJ-5 | ZJ-5 | ZJ-5 | EA(A) |
| | A/Duck/Jiangxi/01.14_NCJD028-P/2015 | G21 | China-3 | N8-North American lineage | EA | ZJ-5 | ZJ-5 | ZJ-5 | ZJ-5 | EA(A) |
| | A/duck/Jiangxi/01.14_NCJD027-P/2015_Mixed | G22 | China-3 | N8-North American lineage | EA | ZJ-5 | ZJ-5 | ZJ-5 | ZJ-5 | EA(B) |
| | A/Environment/Guangxi/32891/2018 | G23 | China-2 | N8-North American lineage | ZJ-5 | ZJ-5 | ZJ-5 | ZJ-5 | ZJ-5 | EA(A) |
| | A/Environment/Guangxi/03561/2019 | G24 | Worldwide-1 | N8-Eurasian lineage | EA | EA | EA | EA | EA | EA(A) |
| | A/Environment/Fujian/10643/2022 | G25 | China-1 | N8-North American lineage | H9N2 | H9N2 | H9N2 | H9N2 | H9N2 | H9N2 |
| | A/Environment/Fujian/10648/2022 | G25 | China-1 | N8-North American lineage | H9N2 | H9N2 | H9N2 | H9N2 | H9N2 | H9N2 |
| | A/Environment/Guangxi/04905/2022 | G25 | China-1 | N8-North American lineage | H9N2 | H9N2 | H9N2 | H9N2 | H9N2 | H9N2 |
| | A/Henan/4-10CNIC/2022 | G25 | China-1 | N8-North American lineage | H9N2 | H9N2 | H9N2 | H9N2 | H9N2 | H9N2 |
| | A/Changsha/1000/2022 | G25 | China-1 | N8-North American lineage | H9N2 | H9N2 | H9N2 | H9N2 | H9N2 | H9N2 |

*Abbreviation of sublineages in internal genes: ZJ-5, ZJ-5 sublineage (gene pool); H6, waterfowl H6; H9N2, poultry H9N2(ZJ-HJ/07); H5N6, H5N6 sublineage; H5N1, poultry H5N1; EA, Eurasian wild bird gene pool; EA(A), Eurasian wild bird gene pool (allele A); EA(B), Eurasian wild bird gene pool (allele B); NA, North American wild bird gene pool.

**Appendix Table 5.** List of key molecular markers of the avian H3 subtype viruses and human H3N8 viruses in China

| Protein | Biological Effect | Mutations | Amino Acids | Avian H3N2 | Avian H3N3 | Avian H3N6 | Avian H3N8 | Avian H3N0/N1/N9# | Human H3N8 |
|---|---|---|---|---|---|---|---|---|---|
| HA | Altered receptor specificity | E190D | E | 289 | 19 | 32 | 54 | 80 | 2 |
| | | | D | 2 | 0 | 0 | 0 | 1 | 0 |
| | | Q226L | Q | 291 | 19 | 32 | 54 | 81 | 2 |
| | | G228S | G | 291 | 19 | 32 | 54 | 81 | 1 |
| | | | S/G | 0 | 0 | 0 | 0 | 0 | 1 |
| NA | Reduced susceptibility to neuraminidase inhibitors | E119V/A/D* | E | 309 | 18 | 27 | 49 | - | 2 |
| | | | G | 1 | 0 | 0 | 0 | - | 0 |
| | | Q136L* | Q | 310 | 18 | 27 | 48 | - | 2 |
| | | | L | 0 | 0 | 0 | 1 | - | 0 |
| | | H274Y* | H | 309 | 18 | 27 | 49 | - | 2 |
| | | | R | 1 | 0 | 0 | 0 | - | 0 |
| PB2 | Increase polymerase activity in avian and mammalian cell line | I292V | I | 214 | 14 | 29 | 36 | 2 | 0 |
| | | | V | 19 | 2 | 3 | 7 | 0 | 2 |
| | | | M | 0 | 0 | 0 | 1 | 0 | 0 |
| | | | T | 0 | 0 | 0 | 0 | 1 | 0 |
| | | R389K | R | 221 | 16 | 32 | 39 | 3 | 0 |
| | | | K | 11 | 0 | 0 | 5 | 0 | 2 |
| | Efficient replication in mammalian and avian cells, and higher virulence in mice | A588V | A | 225 | 16 | 25 | 43 | 2 | 0 |
| | | | T | 4 | 0 | 5 | 0 | 1 | 0 |
| | | | V | 3 | 0 | 2 | 1 | 0 | 2 |
| | Increase the virulence in mammals | V598T/I | T | 231 | 16 | 32 | 41 | 3 | 0 |
| | | | V | 1 | 0 | 0 | 3 | 0 | 2 |
| | | E627K | E | 232 | 16 | 32 | 44 | 3 | 0 |
| | | | K | 0 | 0 | 0 | 0 | 0 | 1† |
| | | | V | 0 | 0 | 0 | 0 | 0 | 1‡ |
| | | D701N | D | 232 | 16 | 32 | 44 | 3 | 2 |
| | Host signature amino acids (avian to human) | K702R | K | 232 | 16 | 32 | 41 | 3 | 1 |
| | | | R | 0 | 0 | 0 | 3 | 0 | 1 |
| PB1 | Increase replication or virulence in mammals | I368V | I | 226 | 10 | 29 | 38 | 2 | 0 |
| | | | V | 7 | 6 | 3 | 5 | 0 | 2 |
| | | | M | 1 | 0 | 0 | 0 | 0 | 0 |
| PA | Reduced susceptibility to endonuclease inhibitors | I38M/T/S/L | I | 291 | 17 | 32 | 54 | 81 | 2 |
| | | | V | 0 | 2 | 0 | 0 | 0 | 0 |
| | Host signature amino acids (avian to human) | K356R | K | 0 | 15 | 31 | 41 | 2 | 0 |
| | | | R | 6 | 0 | 1 | 3 | 0 | 2 |
| | | S409N | S | 225 | 15 | 28 | 40 | 2 | 0 |
| | | | N | 5 | 0 | 4 | 4 | 0 | 2 |
| M1 | Altered virulence in mice | N30D | D | 237 | 17 | 33 | 49 | 2 | 2 |
| | Impacts growth and transmission in the guinea pig model | P41A | A | 237 | 17 | 33 | 49 | 2 | 2 |
| | Altered virulence in mice | T215A | A | 237 | 17 | 33 | 49 | 2 | 2 |
| | Enhanced infectivity | T37A | T | 237 | 17 | 31 | 44 | 2 | 0 |
| | | | A | 0 | 0 | 2 | 5 | 0 | 2 |
| | | R95K | R | 237 | 16 | 33 | 43 | 2 | 0 |
| | | | K | 0 | 1 | 0 | 6 | 0 | 2 |
| | | S224N | S | 237 | 16 | 31 | 42 | 2 | 0 |
| | | | N | 0 | 1 | 2 | 7 | 0 | 2 |
| | | K242N | K | 237 | 16 | 33 | 46 | 2 | 0 |
| | | | N | 0 | 1 | 0 | 3 | 0 | 2 |
| M2 | Enhanced infectivity | D21G | D | 76 | 11 | 27 | 35 | 2 | 0 |
| | | | G | 157 | 6 | 6 | 14 | 0 | 2 |
| | | | V | 3 | 0 | 0 | 0 | 0 | 0 |
| | | | D/G | 1 | 0 | 0 | 0 | 0 | 0 |
| | Reduced susceptibility to amantadine and rimantadine | V27A | V | 222 | 17 | 30 | 41 | 2 | 2 |
| | | | I | 15 | 0 | 2 | 8 | 0 | 0 |
| | | | A | 0 | 0 | 1 | 0 | 0 | 0 |
| | | S31N/G | S | 226 | 16 | 33 | 46 | 2 | 0 |

| Protein | Biological Effect | Mutations | Amino Acids | Avian H3N2 | Avian H3N3 | Avian H3N6 | Avian H3N8 | Avian H3N0/N1/N9# | Human H3N8 |
|---|---|---|---|---|---|---|---|---|---|
| | | | N | 11 | 1 | 0 | 3 | 0 | 2 |
| NS1 | Altered virulence in mice | P42S | S | 237 | 13 | 31 | 35 | 5 | 2 |
| | | | A | 10 | 3 | 2 | 11 | 1 | 0 |
| | | L103F | F | 234 | 12 | 30 | 32 | 5 | 0 |
| | | | Y | 10 | 3 | 2 | 11 | 1 | 0 |
| | | | L | 3 | 1 | 1 | 3 | 0 | 2 |
| | | I106M | M | 246 | 15 | 32 | 43 | 6 | 0 |
| | | | I | 1 | 1 | 1 | 3 | 0 | 2 |
| | | E227R | E | 246 | 15 | 32 | 43 | 6 | 0 |
| | | | K | 1 | 1 | 1 | 3 | 0 | 2 |
| | | S228P | S | 246 | 15 | 32 | 43 | 6 | 0 |
| | | | P | 1 | 1 | 1 | 3 | 0 | 2 |

*N2 numbering system was used.
#NA genes of H3N1 and H3N9 avian influenza viruses were not included in this analysis. Key molecular markers of each of H3N8 G25 viruses are shown in Appendix Figure 14.
†A/Henan/4-10CNIC/2022(H3N8).
‡A/Changsha/1000/2022(H3N8).

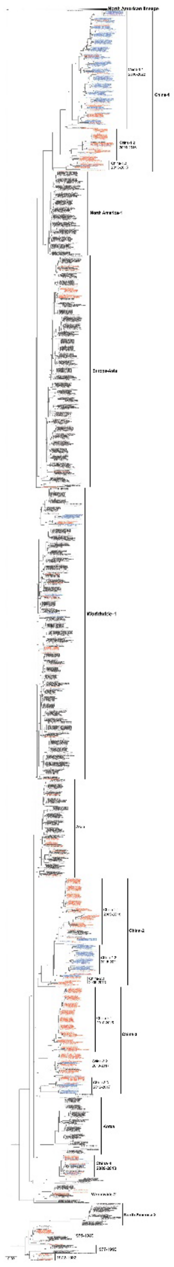

**Appendix Figure 1.** Phylogenetic tree of H3 hemagglutinin (HA) genes. Maximum likelihood tree (n=2871). H3 strains sequenced in this study are in steel blue. Other H3 AIVs in China are in red. Two human viruses are in violet and marked with triangles. A mini group containing two human and three environmental H3N8 viruses is denoted in the dashed box. Branch lengths are scaled according to the numbers of substitutions per site. Branch support values of selected nodes are shown. Sublineages and subgroups are labelled with vertical lines on the right.

A

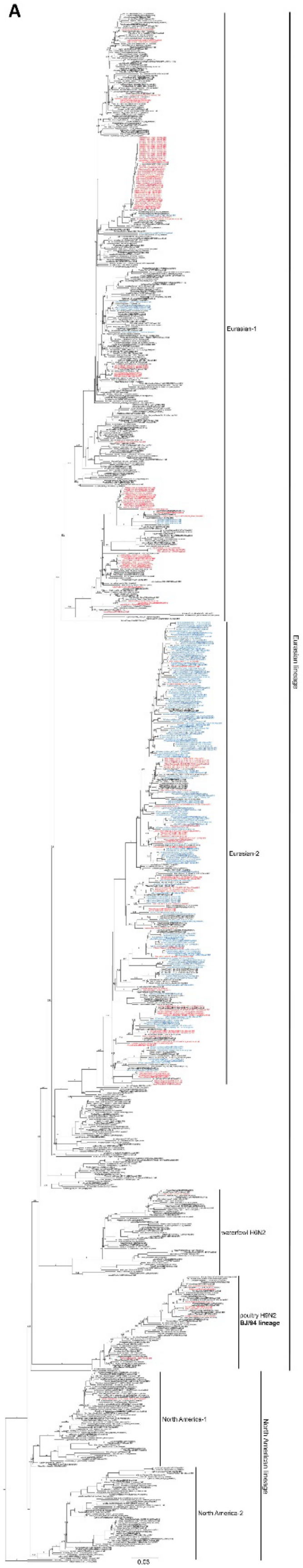
Eurasian-1
Eurasian-2
waterfowl H6N2
poultry H6N2
BJ/94 lineage
North America-1
North America-2
Eurasian lineage
North American lineage
0.03

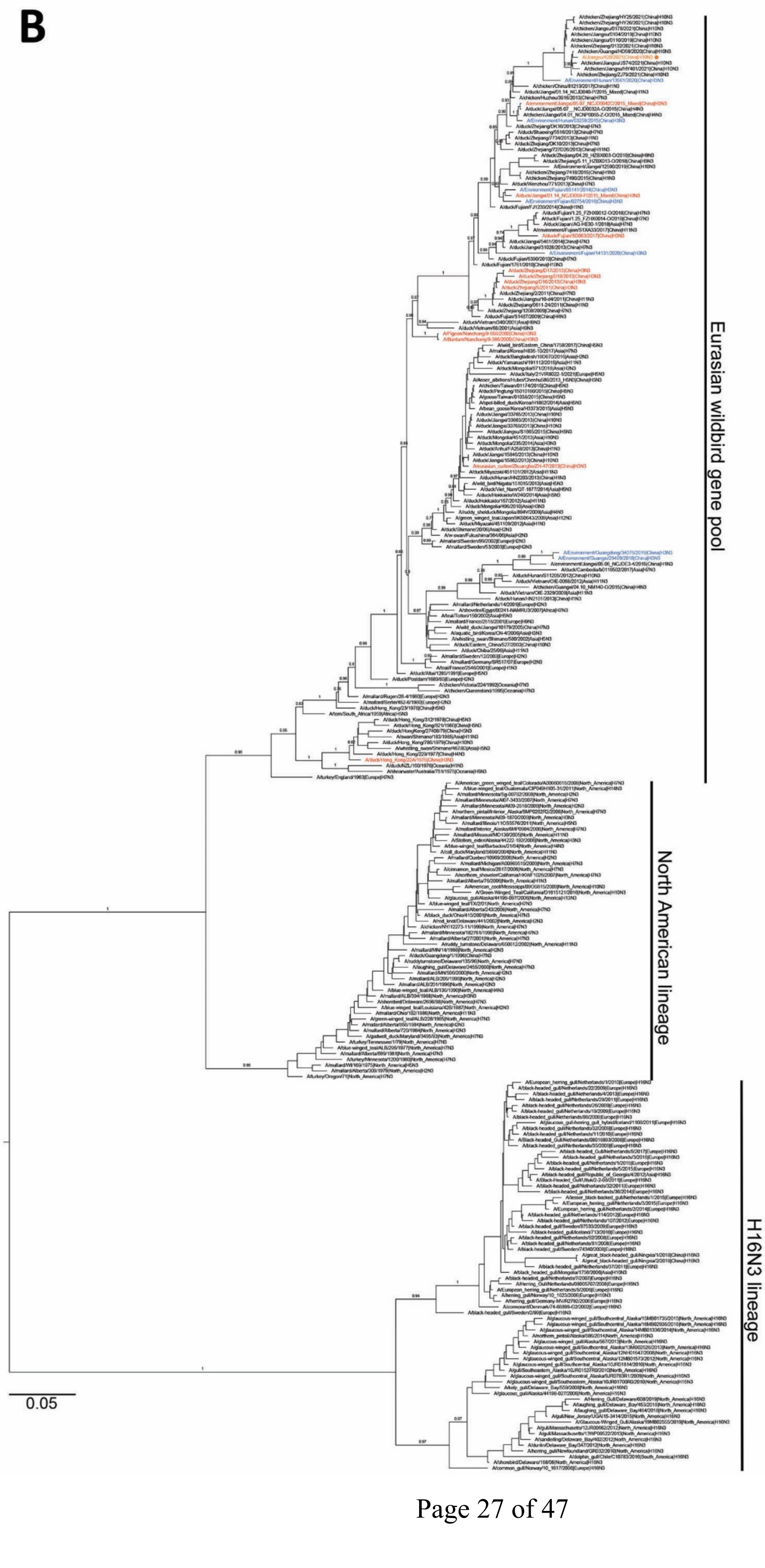
B
Eurasian wildbird gene pool
North American lineage
H16N3 lineage
0.05

**C**

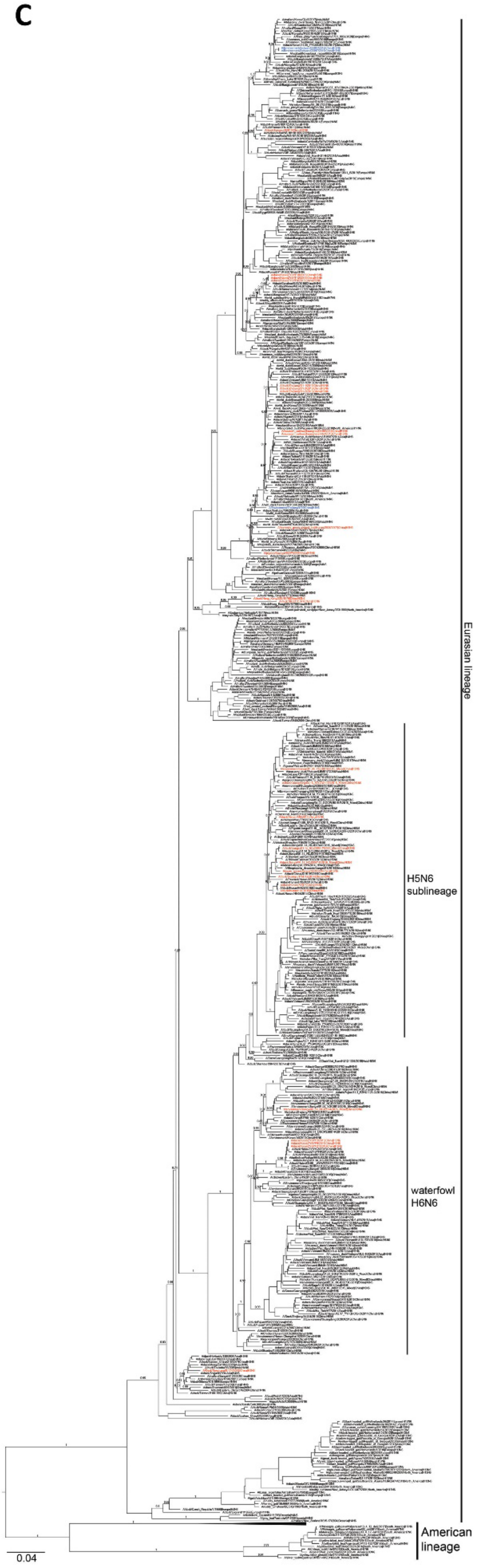
Eurasian lineage
H5N6 sublineage
waterfowl H6N6
American lineage
0.04

**D**

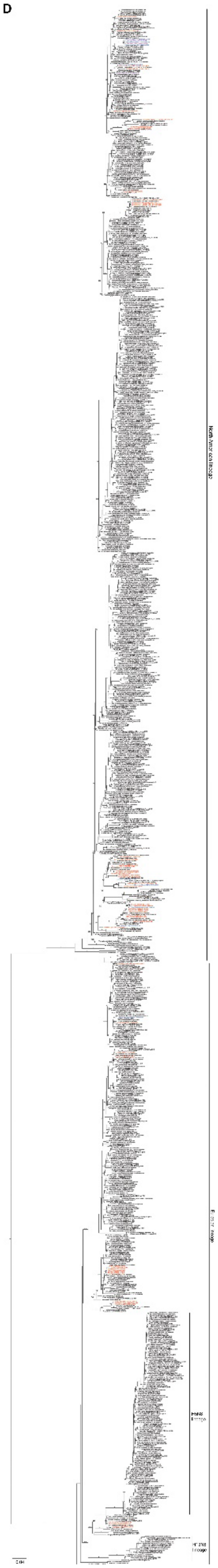

**Appendix Figure 2.** Phylogenetic trees of neuraminidase (NA) genes. Maximum likelihood tree of N2 (n=876, A), N3 (n=253, B), N6 (n=941, C), and N8 (n=1106, D) genes. H3 strains sequenced in this study are in steel blue. Other H3 AIVs in China are in red. For N3 gene (B), human H10N3 virus is indicated by orange and marked with a dot. For N8 gene (D), human H3N8 and H10N8 viruses are indicated by violet (triangles) and purple (dot), respectively. Branch lengths are scaled according to the numbers of substitutions per site. Branch support values of selected nodes are shown. Sublineages and subgroups are labelled with vertical lines on the right.

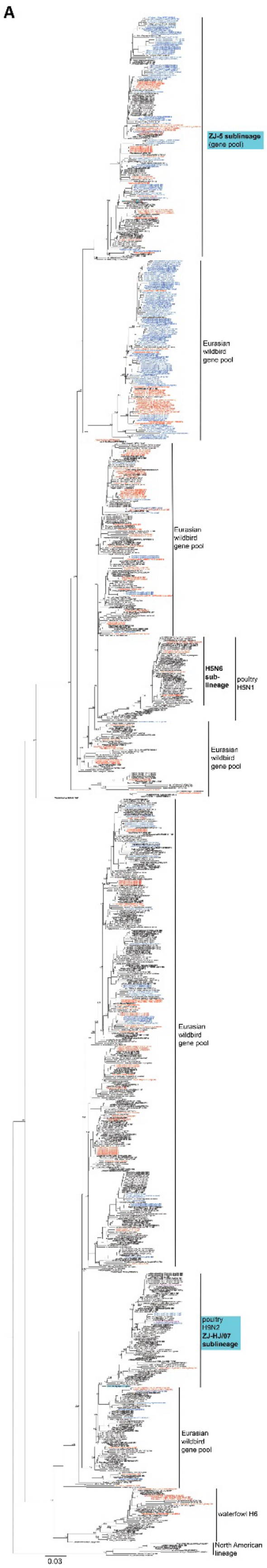
A
ZJ-5 sublineage
(gene pool)
Eurasian
wildbird
gene pool
Eurasian
wildbird
gene pool
H5N6
sub-
lineage
poultry
H5N1
Eurasian
wildbird
gene pool
Eurasian
wildbird
gene pool
poultry
H9N2
ZJ-HJ/07
sublineage
Eurasian
wildbird
gene pool
waterfowl H6
North American
lineage
0.03

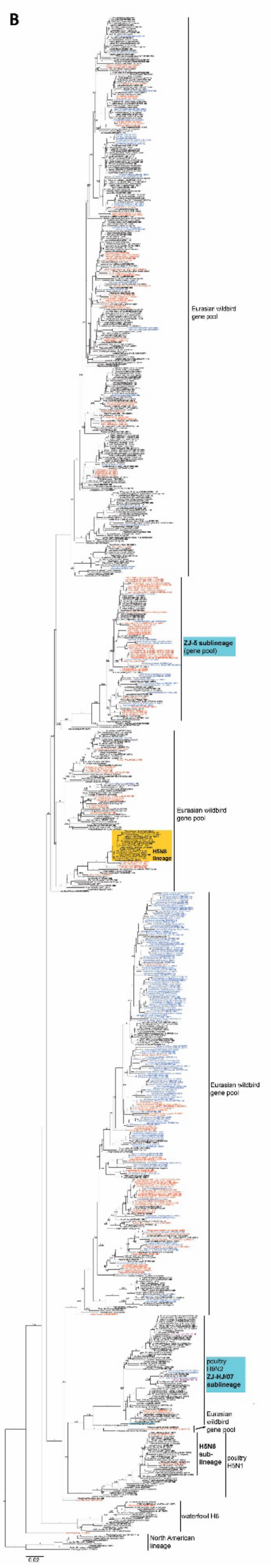
B
Eurasian wildbird gene pool
ZJ-5 sublineage (gene pool)
Eurasian wildbird gene pool
H5N8 lineage
Eurasian wildbird gene pool
poultry H9N2 ZJ-HJ07 sublineage
Eurasian wildbird gene pool
H5N8 sub-lineage
poultry H5N1
waterfowl H6
North American lineage
0.02

**C**

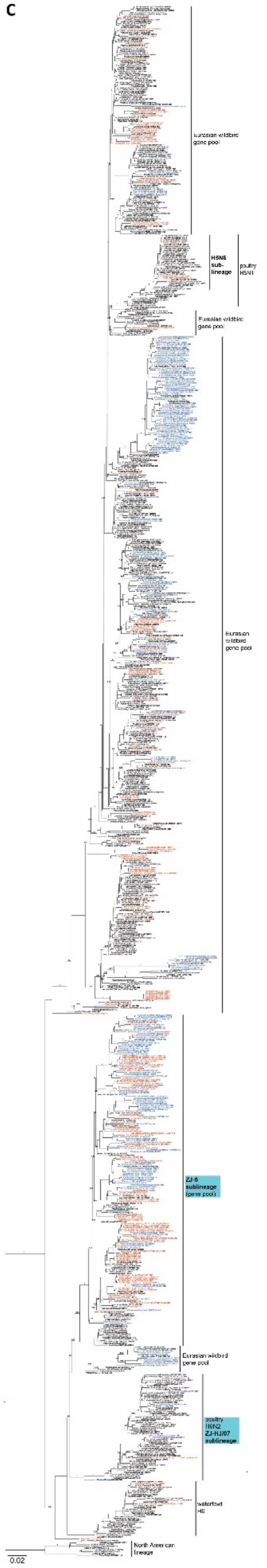
Eurasian wildbird gene pool
H5N6 sub-lineage
poultry H5N1
Eurasian wildbird gene pool
Eurasian wildbird gene pool
ZJ-8 sublineage (gene pool)
Eurasian wildbird gene pool
poultry H6N2 ZJ-HJ/07 sublineage
waterfowl H6
North American lineage
0.02

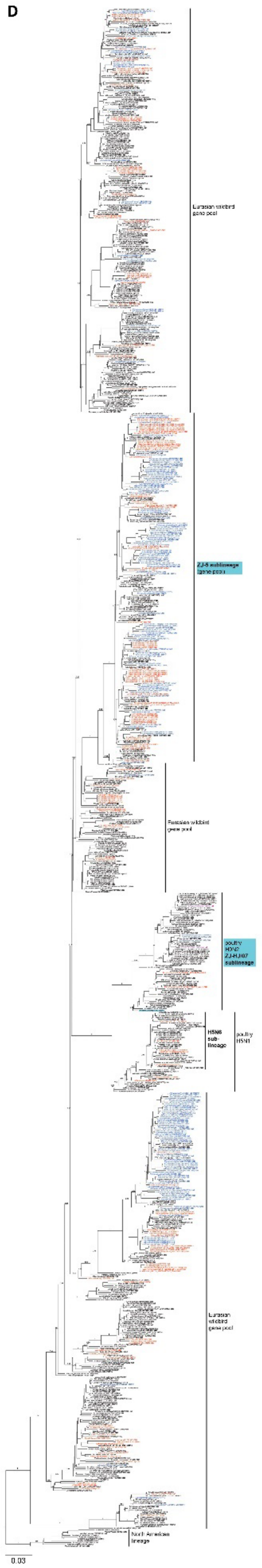
D
Eurasian wildbird gene pool
ZJ-5 sublineage gene pool
Eurasian wildbird gene pool
poultry H9N2 ZJ-HJ/07 sublineage
H5N6 sub-lineage
poultry H5N1
Eurasian wildbird gene pool
North American lineage
0.03

E

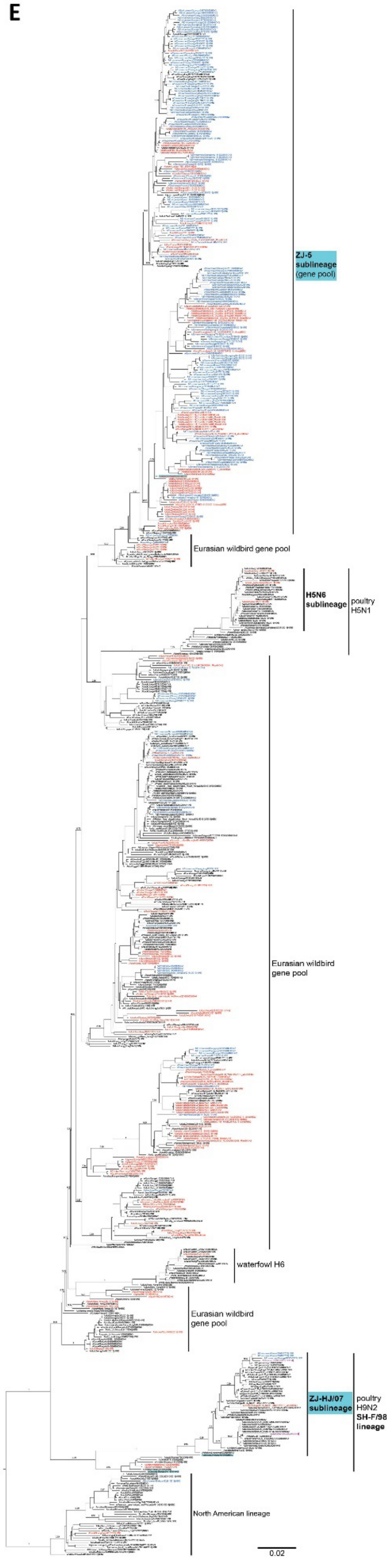
ZJ-5
sublineage
(gene pool)
Eurasian wildbird gene pool
H5N6
sublineage
poultry
H5N1
Eurasian wildbird
gene pool
waterfowl H6
Eurasian wildbird
gene pool
ZJ-HJ/07
sublineage
poultry
H9N2
SH-F/98
lineage
North American lineage
0.02

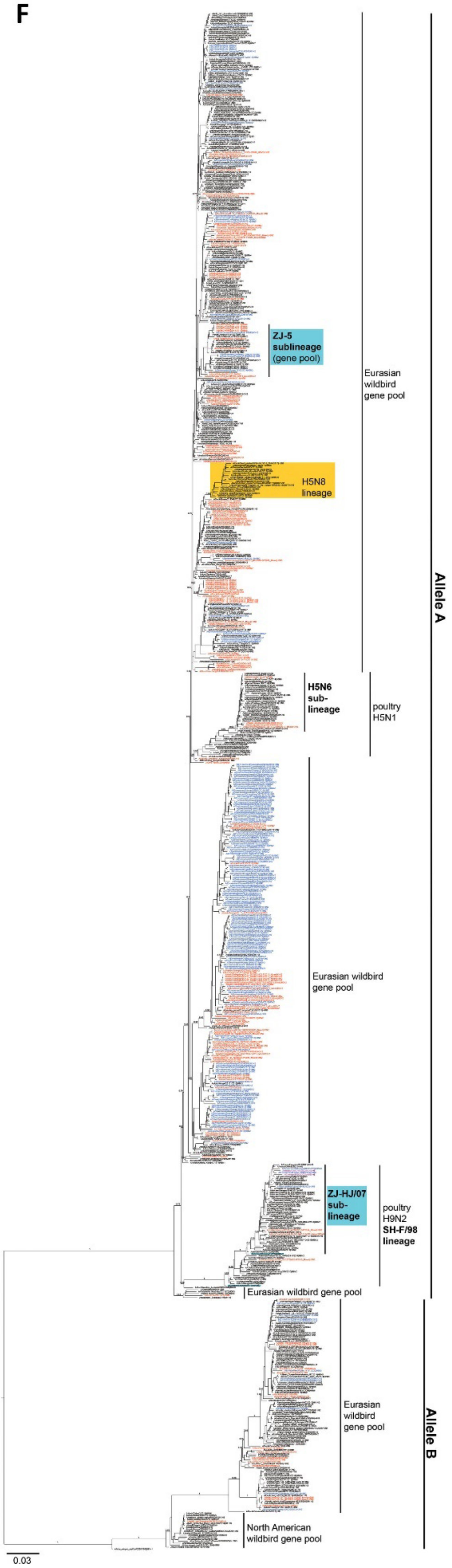

F
ZJ-5
sublineage
(gene pool)
Eurasian
wildbird
gene pool
H5N8
lineage
Allele A
H5N6
sub-
lineage
poultry
H5N1
Eurasian wildbird
gene pool
ZJ-HJ/07
sub-
lineage
poultry
H9N2
SH-F/98
lineage
Eurasian wildbird gene pool
Eurasian
wildbird
gene pool
Allele B
North American
wildbird gene pool
0.03

**Appendix Figure 3.** Phylogenetic trees of internal genes. Maximum likelihood trees of PB2 (n=929, A), PB1 (n=841, B), PA (n=841, C), NP (n=856, D), M (n=654, E), and NS (n=776, F) genes. H3 strains sequenced in this study are in steel blue. Other H3 AIVs in China are in red. The human H3N8 viruses are indicated by violet and triangles. The ZJ-5 sublineage and the poultry H9N2 ZJ-HJ/07 sublineage as well as their representative virus strains are highlighted in light-blue blocks. Branch lengths are scaled according to the numbers of substitutions per site. Branch support values of selected nodes are shown. Sublineages and subgroups are labelled with vertical lines on the right.

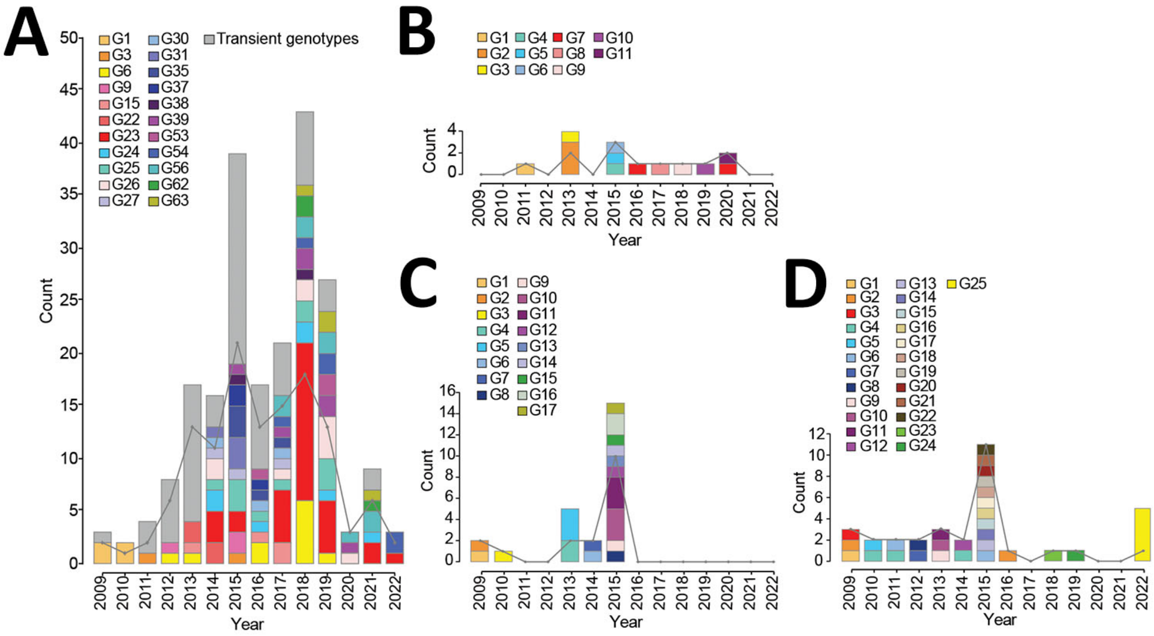


**Appendix Figure 4**. Diversity of genotypes of H3 subtype viruses sampled in China during 2009–2022. (A-D) Temporal distribution of genotypes of H3N2(A), H3N3(B), H3N6 (C), and H3N8 (D) AIVs. The counts of genotypes (line) and isolates of each genotype (column) are shown. For the H3N2 AIVs (A), grey column indicates the sum of isolates of transient genotypes. Detailed information for all genotypes can be found in Appendix Table 4.

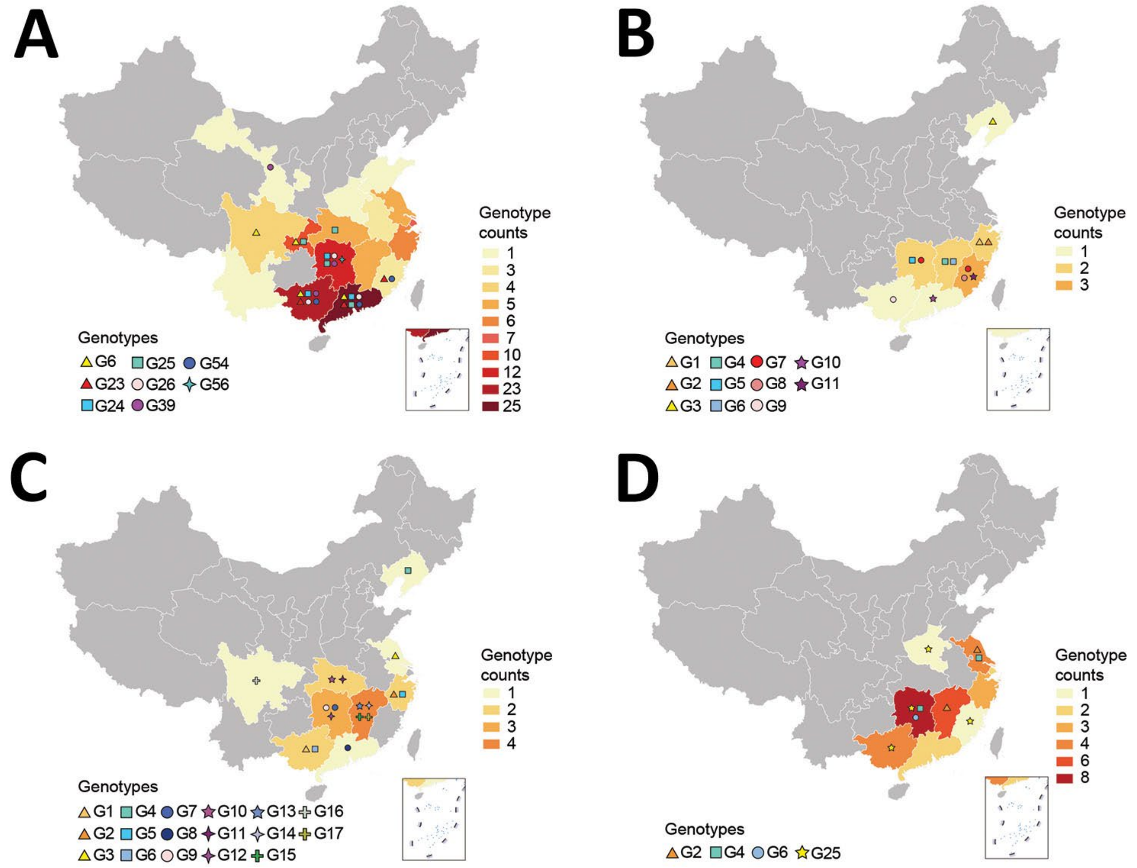


**Appendix Figure 5.** Geographical distribution of genotypes of H3 subtype viruses sampled in China during 2009–2022. (A-D) Geographical distributions of genotypes of H3N2 (A), H3N3 (B), H3N6 (C), and H3N8 (D) AIVs. The color of each province represents the number of genotypes. Provinces without applicable data are in grey. Different genotypes are marked with colored symbols on the map. For H3N2 (A) and H3N8 (D) AIVs, only the major genotypes are shown on the map. Detailed information for all genotypes can be found in Appendix Table 4.

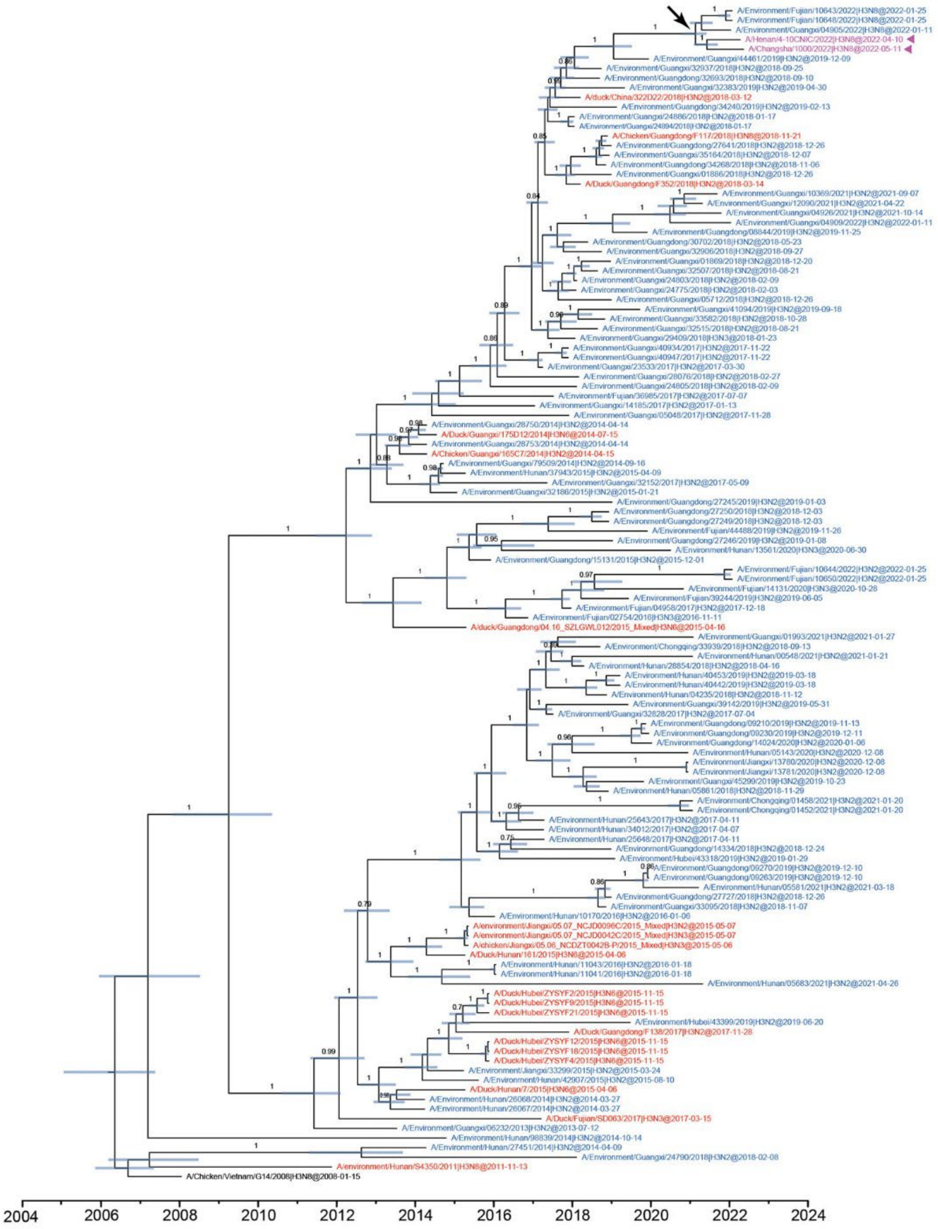


**Appendix Figure 6.** Maximum clade credibility tree of H3 hemagglutinin (HA) sequences of the China-1.1 subgroup viruses (n=122). H3 strains sequenced in this study are in steel blue. Other H3 AIVs in China are in red. Two human H3N8 viruses are indicated by violet and triangles. The timescale bar is provided at the bottom. Clade posterior probabilities and 95% highest posterior density (HPD) of age estimates of selected nodes are shown as numbers and horizontal bars respectively. The most recent common ancestor of H3N8 G25 viruses is marked with arrow.

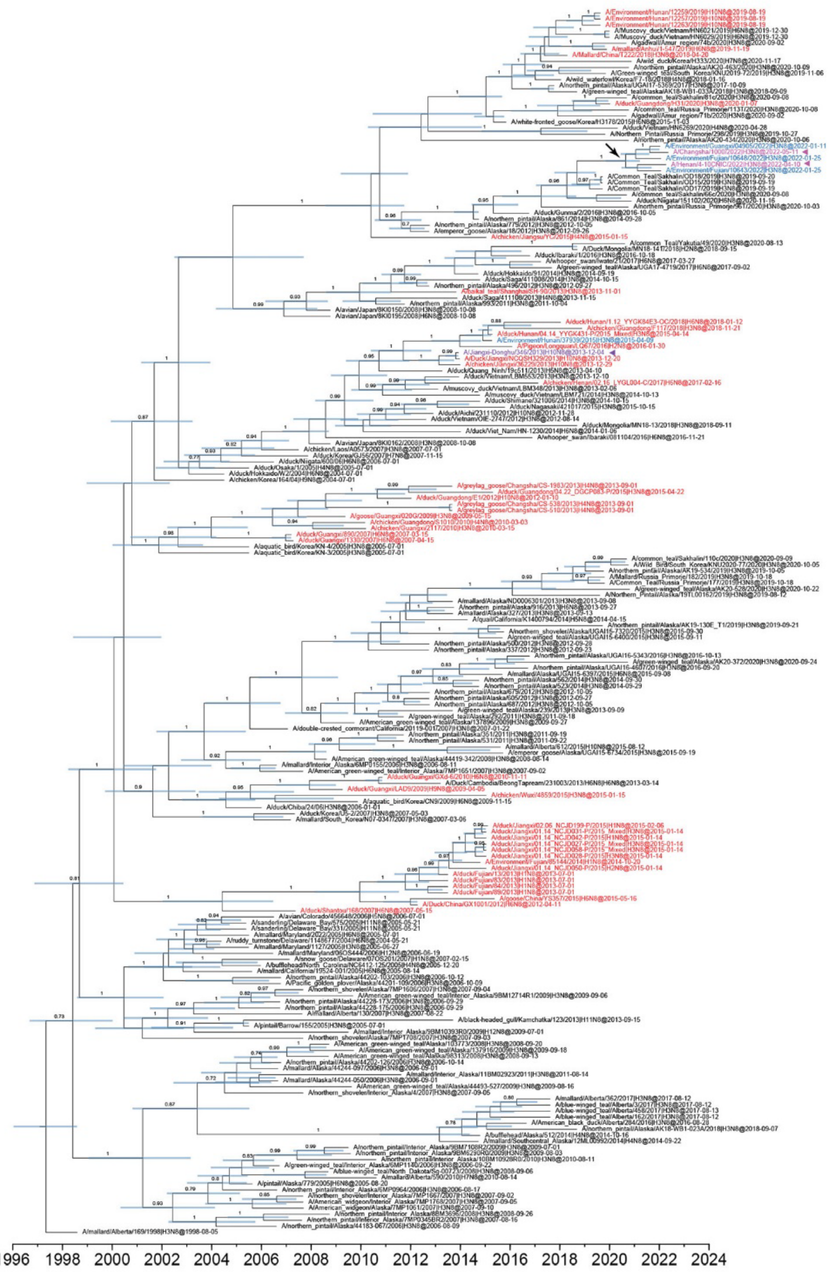


**Appendix Figure 7.** Maximum clade credibility tree of N8 neuraminidase (NA) sequences (n=202). H3 strains sequenced in this study are in steel blue. Other H3 AIVs in China are in red. Human H3N8 and H10N8 viruses are indicated by violet and purple, respectively. The timescale bar is provided at the bottom. Clade posterior probabilities and 95% HPD of age estimates of selected nodes are shown as numbers and horizontal bars respectively. The most recent common ancestor of H3N8 G25 viruses is marked with arrow.

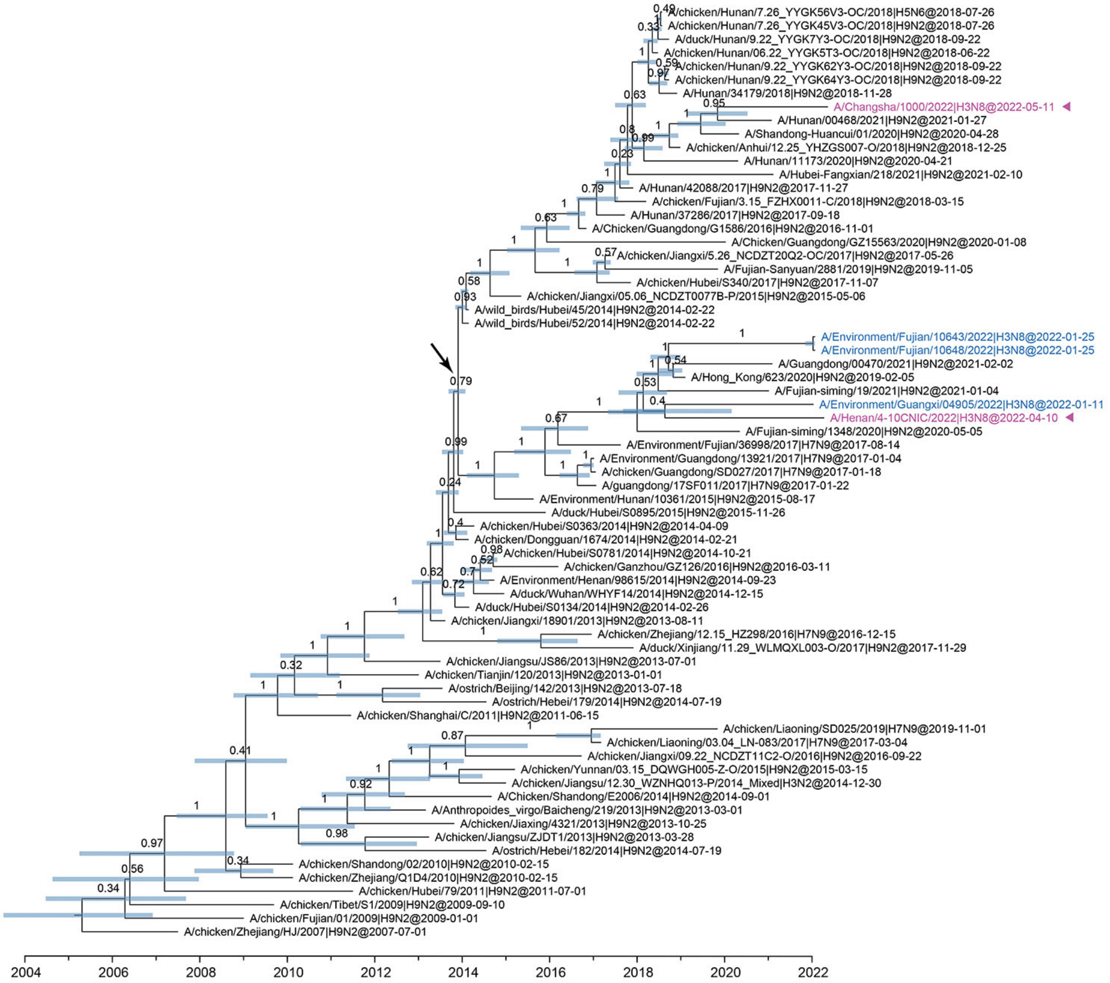


**Appendix Figure 8.** Maximum clade credibility tree of PB2 gene (n=69). H3 strains sequenced in this study are in steel blue. Two human H3N8 viruses are indicated by violet and triangles. The timescale bar is provided at the bottom. Clade posterior probabilities and 95% highest posterior density (HPD) of age estimates of selected nodes are shown as numbers and horizontal bars respectively. The most recent common ancestor of H3N8 G25 viruses is marked with arrow.

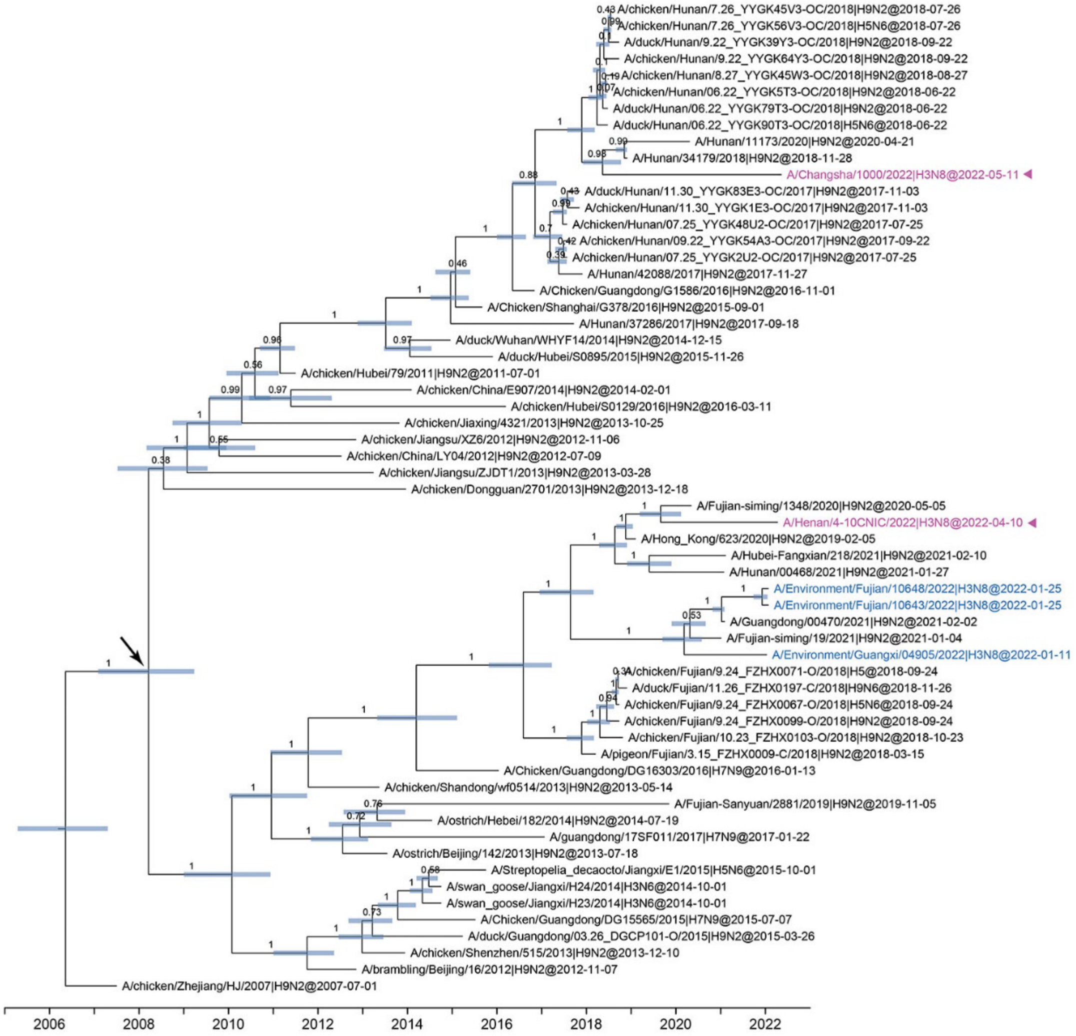


**Appendix Figure 9.** Maximum clade credibility tree of PB1 gene (n=60). Annotation as in Appendix Figure 8.

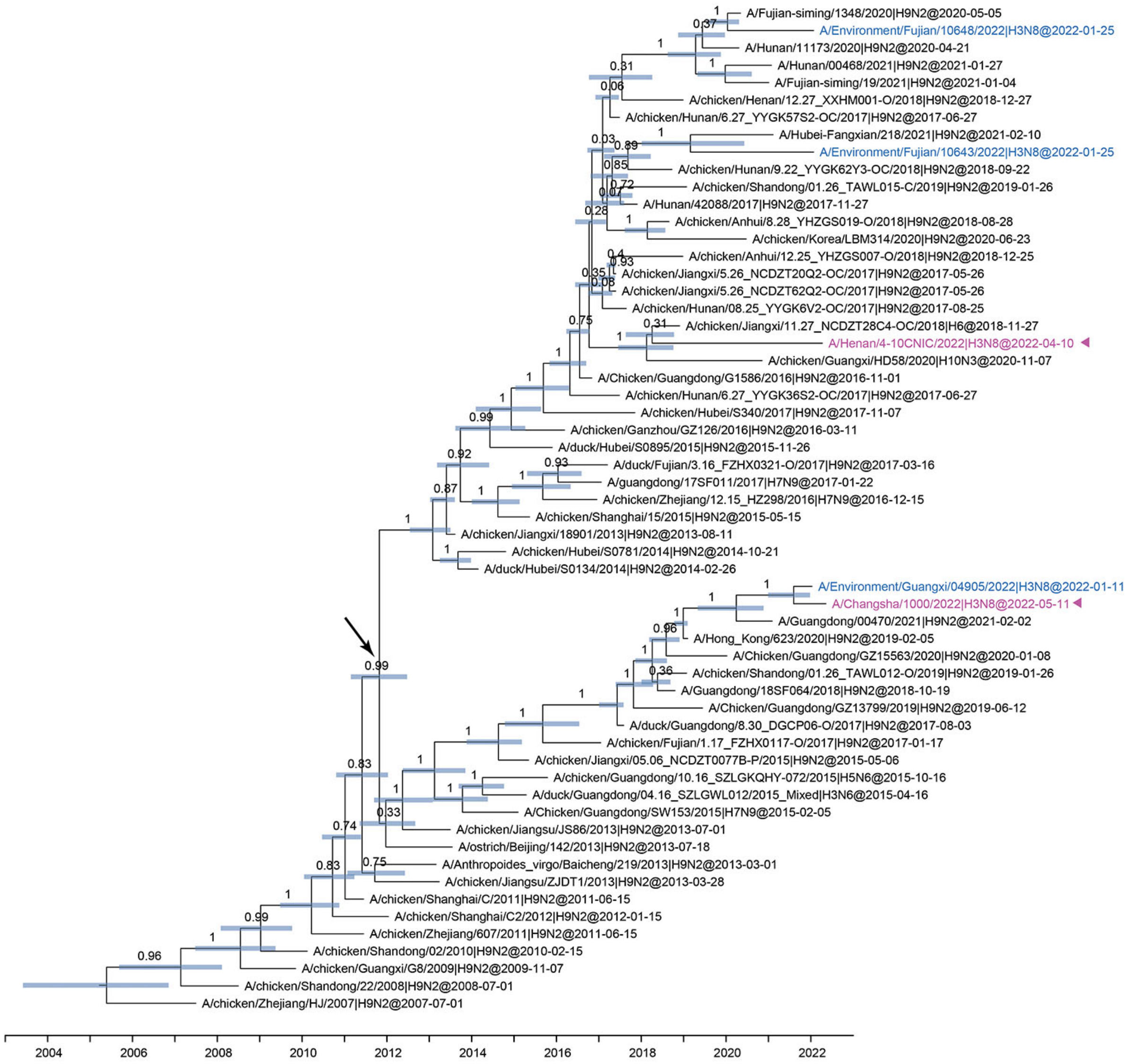


**Appendix Figure 10.** Maximum clade credibility tree of PA gene (n=58). Annotation as in Appendix Figure 8.

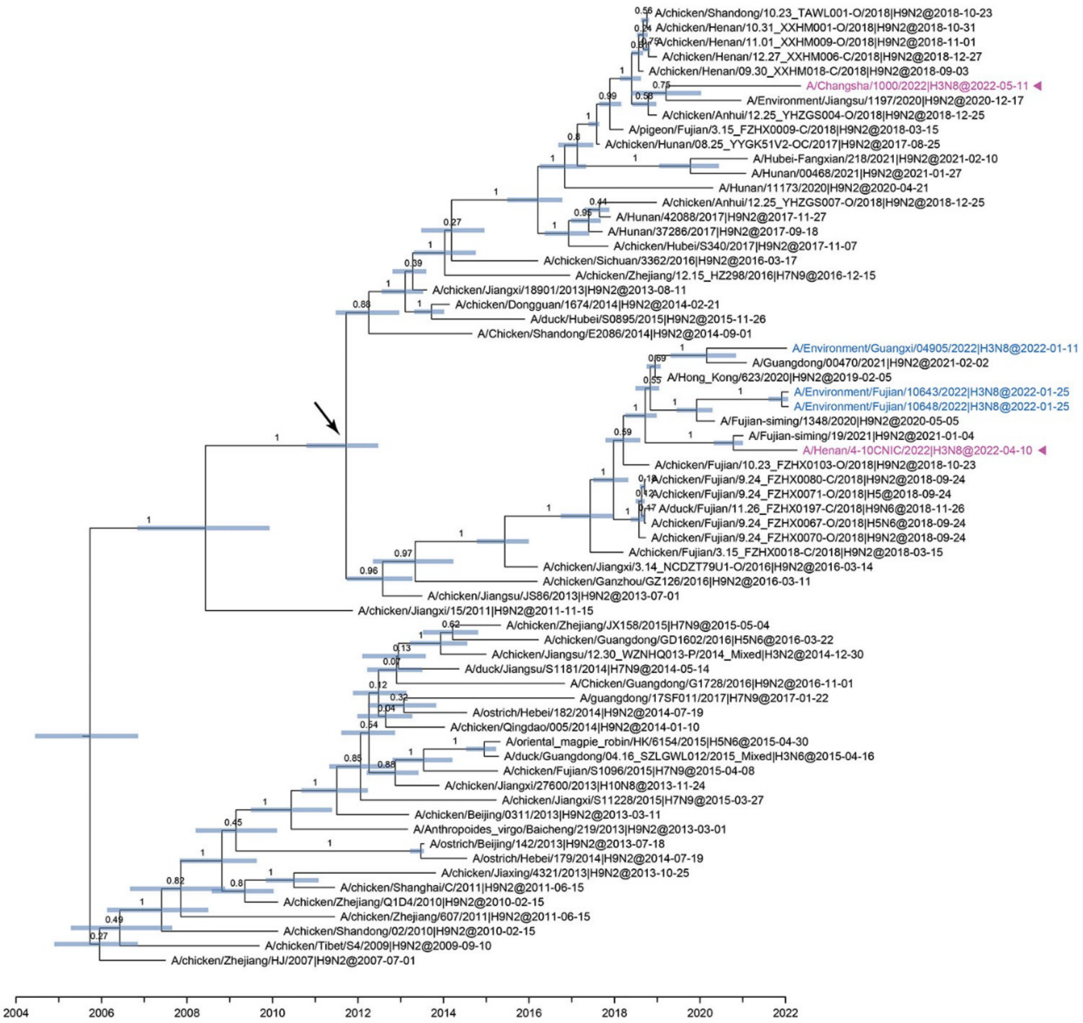

**Appendix Figure 11.** Maximum clade credibility tree of NP gene (n=66). Annotation as in Appendix Figure 8.

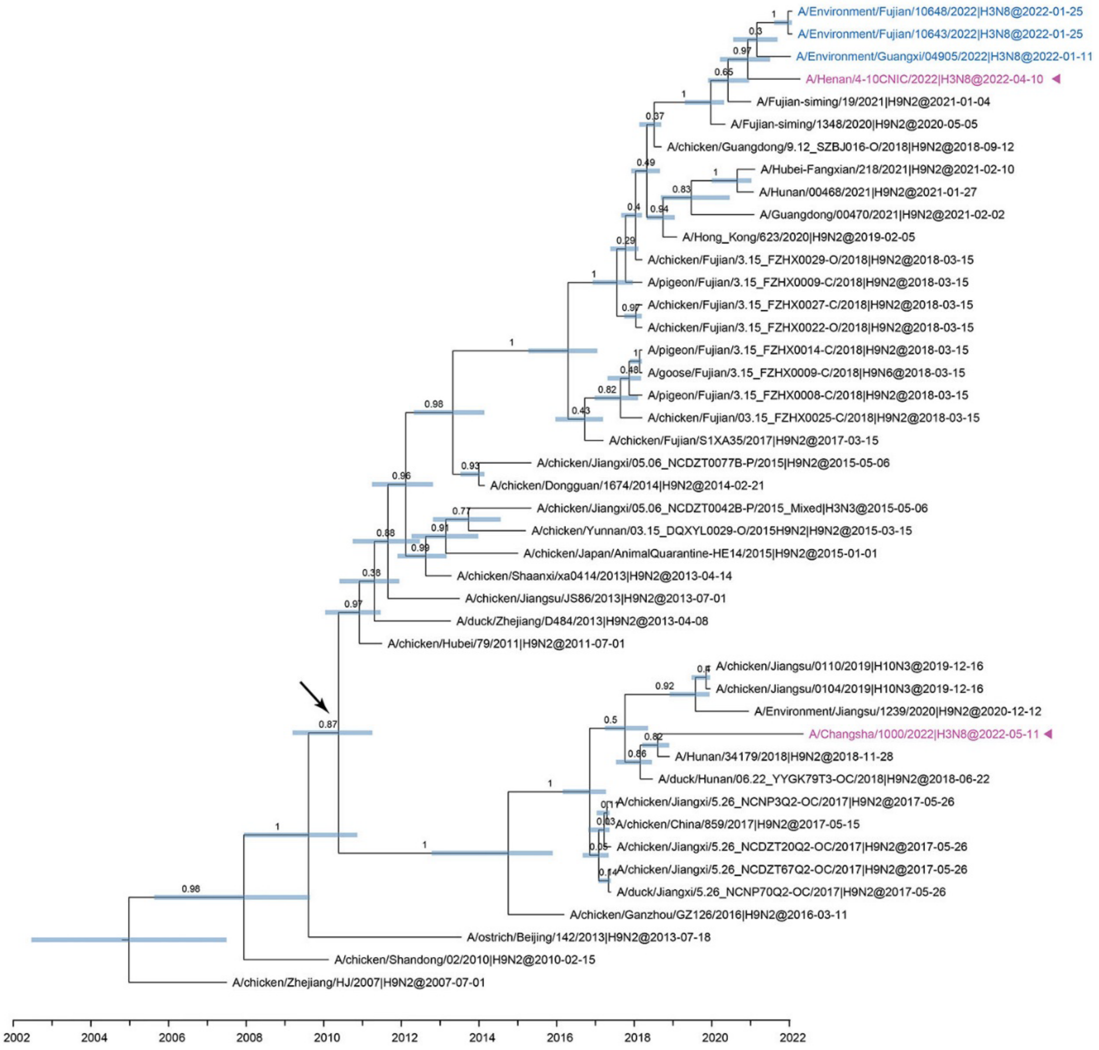


**Appendix Figure 12.** Maximum clade credibility tree of MP gene (n=44). Annotation as in Appendix Figure 8.

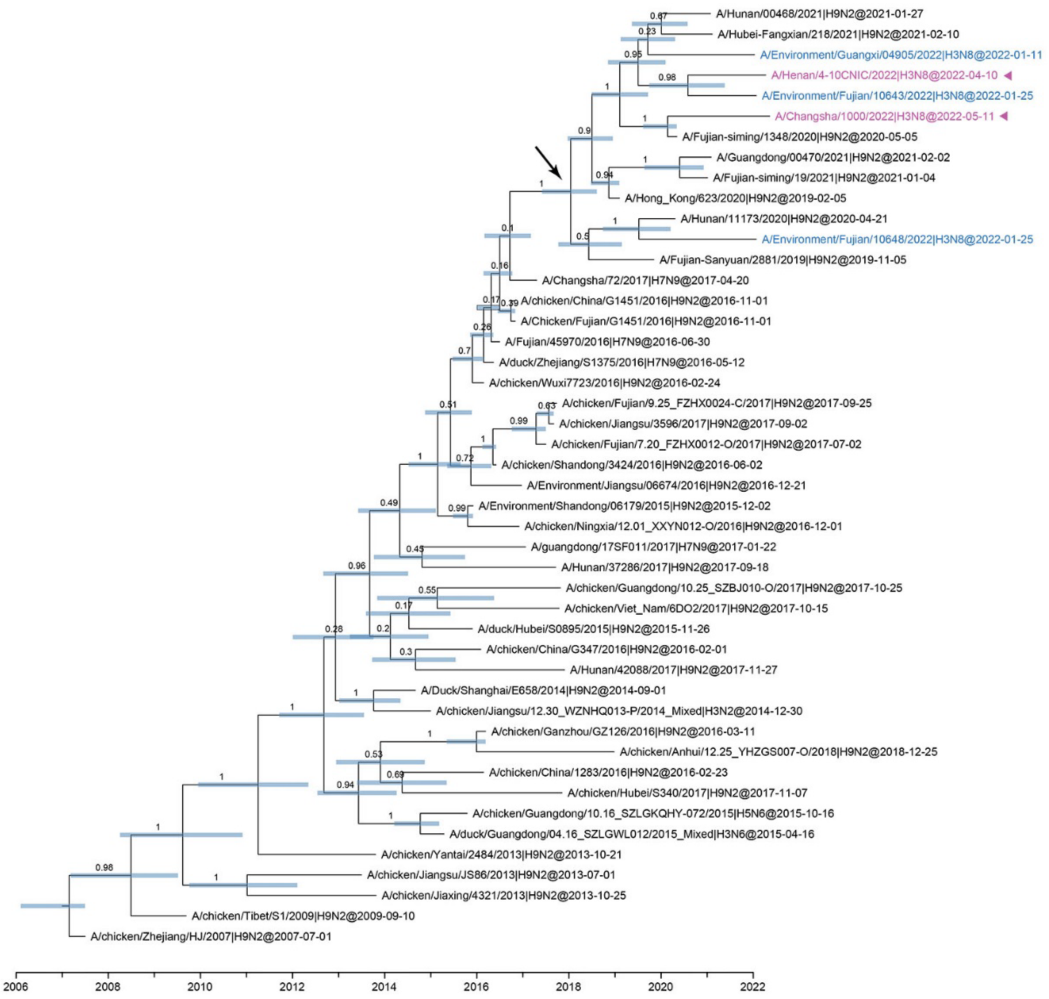


**Appendix Figure 13.** Maximum clade credibility tree of NS genes(n=46). Annotation as in Appendix Figure 8.

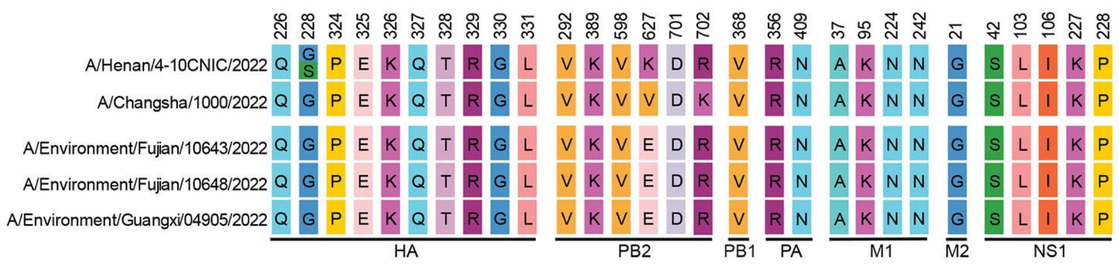


**Appendix Figure 14.** Key molecular markers of H3N8 G25 viruses. Key molecular markers associated with mammalian adaption are shown.

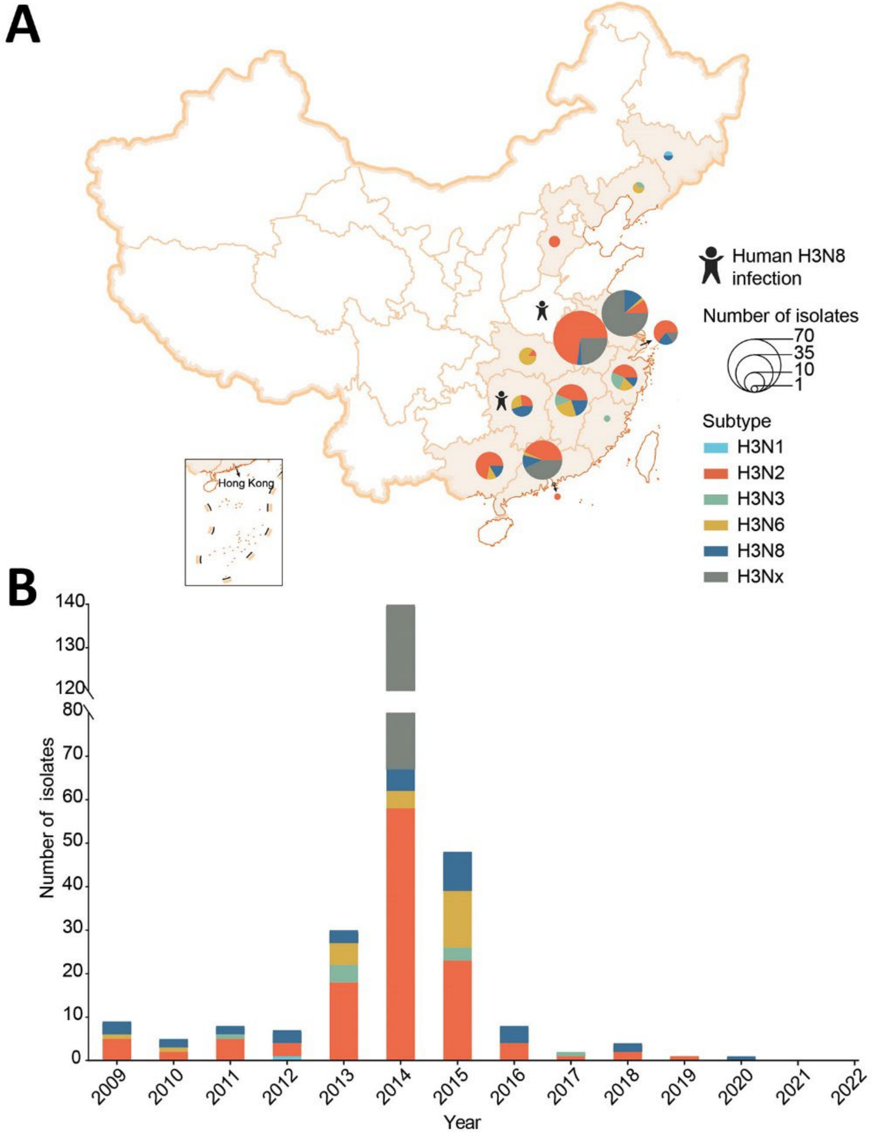


**Appendix Figure 15.** Spatiotemporal distribution of H3 hemagglutinin (HA) sequences in China during 2009-2022 available from the GISAID EpiFlu Database. (A) Spatial distribution. Annotation as in Figure 1A. (B) Temporal distribution. The colors of each column correspond to those of different NA subtypes in (A).
